# A roadmap to sustainable management of commercial medicinal and aromatic plants, fungi, and lichens in Nepal

**DOI:** 10.1111/cobi.14442

**Published:** 2025-01-17

**Authors:** Carsten Smith‐Hall, Dipesh Pyakurel, Thorsten Treue, Mariève Pouliot, Suresh Ghimire, Anastasiya Timoshyna, Henrik Meilby

**Affiliations:** ^1^ Department of Food and Resource Economics, Faculty of Science University of Copenhagen Frederiksberg Denmark; ^2^ Asia Network for Sustainable Agriculture and Bioresources Kathmandu Nepal; ^3^ Central Department of Botany, Institute of Science and Technology Tribhuvan University Kathmandu Nepal; ^4^ TRAFFIC Cambridge UK; ^5^ IUCN Medicinal Plant Specialist Group Cambridge UK

**Keywords:** conservation policies, environmental products, Himalaya, illegal wildlife trade, livelihoods, sustainability, Himalaya, mercado ilegal de plantas, políticas de conservación, productos ambientales, sustentabilidad, sustento

## Abstract

Thousands of plants, fungi, and lichen species are traded every year. Although sustainable use is critical for livelihoods and biodiversity conservation, insufficient data prevent detailed sustainability assessments for most species. How can the sustainability of trade in such data‐deficient species be enhanced? We considered a country‐level example of 300 medicinal and aromatic plant, fungus, and lichen species traded in tens of thousands of tons worth tens of millions of US dollars in and from Nepal annually. Past interventions have not ensured sustainable trade, leaving species vulnerable to commercial harvesting and threatening rural household incomes, the processing industry, and government revenues. Building on documented evidence and stakeholder involvement, we used a theory of change approach to develop a sustainable management approach. We produced a draft plan (roadmap) by combining interventions proposed at annual key stakeholder dialogue meetings with recommendations extracted from a literature review on the trade and conservation of commercial medicinal and aromatic plants, fungi, and lichens in Nepal. The draft roadmap was discussed at a national workshop with sector‐wide stakeholder representation to derive the final roadmap. The literature review showed the 41 causal assumptions and theoretical explanations for actions and outcomes. Feedback mechanisms included 6 bundles of mutually reinforcing actions, such as the relationship between increased cultivation and decreased wild harvesting. The roadmap has 5 pathways: increase cultivation, strengthen local management, support domestic businesses, improve sector governance, and increase international collaboration. Each pathway is associated with 2–5 actions (e.g., hand over high‐elevation areas to local communities) that lead to outputs (2‐5) (e.g., an increased area under local management) and outcomes (2‐6) (e.g., less overharvesting). Accordingly, the roadmap offers stakeholders a structured approach to implement future activities and investments to enhance sustainable trade. The approach can be replicated for other countries and products.

## Abstract (es)

Un plan de ruta para el manejo sustentable de plantas aromáticas y medicinales, hongos y líquenes

**Resumen:**

Miles de especies de plantas, hongos y líquenes son objeto de comercio cada año. Aunque el uso sostenible es fundamental para el sustento y para la conservación de la biodiversidad, la falta de datos impide realizar evaluaciones detalladas de la sustentabilidad de la mayoría de las especies. ¿Cómo puede mejorarse la sustentabilidad del comercio de estas especies sobre las que no se dispone de datos suficientes? Consideramos un ejemplo a escala nacional de 300 especies de plantas medicinales y aromáticas, hongos y líquenes con las que se comercian anualmente decenas de miles de toneladas con valor de decenas de millones de dólares estadounidenses en y desde Nepal. Las intervenciones anteriores no han garantizado un comercio sostenible, lo que deja a las especies vulnerables a la recolección comercial y amenaza los ingresos de los hogares rurales, la industria de transformación y los ingresos del gobierno. Con base en pruebas documentadas y en la participación de las partes interesadas, utilizamos una teoría del cambio para desarrollar un enfoque de manejo sustentable. Elaboramos el borrador de un plan de acción que combina las intervenciones propuestas en las reuniones anuales de diálogo con los actores principales con las recomendaciones extraídas de una revisión bibliográfica sobre el comercio y la conservación de plantas medicinales y aromáticas, hongos y líquenes en Nepal. El borrador del plan de acción se debatió en un taller nacional con representación de todos los actores del sector para elaborar el plan de acción definitivo. La revisión bibliográfica mostró los 41 supuestos causales y explicaciones teóricas de acciones y resultados. Los mecanismos de retroalimentación incluyeron seis paquetes de acciones que se reforzaban mutuamente, como la relación entre el aumento del cultivo y la disminución de la recolección silvestre. El plan de acción tiene cinco vías: aumentar el cultivo, reforzar la gestión local, apoyar a las empresas nacionales, mejorar la gobernanza del sector y aumentar la colaboración internacional. Cada vía está asociada a 2‐5 acciones (por ejemplo, traspasar zonas de gran altitud a las comunidades locales) que conducen a productos (2‐5) (por ejemplo, un aumento de la superficie bajo gestión local) y resultados (2‐6) (por ejemplo, menos sobreexplotación). Así, el plan de acción ofrece a los actores un enfoque estructurado para poner en marcha futuras actividades e inversiones que mejoren el comercio sostenible. El enfoque puede reproducirse para otros países y productos.

## INTRODUCTION

There is a huge international trade in plants, fungi, and lichens (Fromentin et al., 2023); approximately 7000 wild plant species (Khoury et al., 2019) and hundreds of fungus species (Alexander et al., 2011) are in trade. These species underpin global food, cosmetics, and health commodity industries and are frequently hidden and unrecognized (Chamberlain & Smith‐Hall, 2024). Many species are harvested in high volumes for domestic and international markets in locations with weak enforcement of regulations and strong drivers of collection, including poverty (Arias et al., 2024), facilitated by well‐established markets with actors providing services, such as advance payments to harvesters (Fold et al., 2023). Although markets are growing and becoming increasingly formalized, their scale and rate of change are of conservation concern (Barron et al., 2022; Cunningham, Brinckmann, Yang, et al., 2019; Maxwell et al., 2016). Hence, ensuring sustainable and legal harvesting and trade in wild‐harvested species is among the global targets for urgent action in the Kunming–Montreal Global Biodiversity Framework (CBD, 2022).

Acknowledging that sustainability is influenced by many factors, including taxonomic group and site characteristics (Leão et al., 2017), we asked: how can sustainable management of plants, fungi, and lichens be enhanced at the national level, where a large number of species are commercially harvested and many face increasing prices and demand, when few species‐specific data exist (e.g., on sustainable harvesting levels and methods)?

We applied a collaborative theory of change approach, including sector‐wide discussions and building on literature to identify assumptions and constraints, to the case of commercial medicinal and aromatic plants, fungi, and lichens in Nepal to develop a plan to enhance the sustainability of trade in these groups.

### Medicinal and aromatic plant, fungus, and lichen trade in and from Nepal

This trade encompasses 300 species, most native and destructively harvested from the wild (Pyakurel et al., 2019). Following Smith‐Hall et al. (2018), we define *commercial medicinal* and *aromatic plants*, *fungi*, and *lichens* as species traded to produce pharmaceuticals, dietary supplements, natural health products, cosmetics and other personal care, and culinary products. The annual volume of trade was 7000–27,000 t worth US$11–48 million in 1998 (2020 inflation‐adjusted values from Olsen [2005a]) and 10,770 t worth US$86 million in 2014 (in 2020, values from Ghimire et al. [2016]). Income from these species constitutes a substantial share of rural household incomes, particularly in high‐elevation communities (Olsen & Larsen, 2003; Timmerman & Smith‐Hall, 2019), and they form one of Nepal's most valuable export commodities (Olsen & Helles, 2009). Most products are air‐dried, traded through a well‐established production network of harvesters, traders, and wholesalers, and destined for secondary processing in India and China (Caporale et al., 2020; Fold et al., 2023; He et al., 2018; Pyakurel et al., 2018). Nepal's processing sector has expanded rapidly in the past 2 decades (Chapagain et al., 2024). An unspecified but substantial amount of the trade is undocumented and illegal.

The trade is dynamic, with price fluctuations and changes in species composition (Pyakurel et al., 2018), and is expected to increase due to rising consumer incomes and new infrastructure, including the Belt and Road Initiative (Hinsley et al., 2020), and e‐commerce (Cunningham et al., 2019). Despite the trade's long history, detailed species‐level sustainability assessments are rare, even if such work is emerging (Smith‐Hall et al., 2023). Comprehensive product‐level consumption surveys do not exist (Smith‐Hall et al., 2020). The large number of commercial species and their economic importance, the increasing trade (legal and illegal), and the lack of sustainability studies and knowledge of consumption drivers emphasize the need for a structured approach to enable species conservation and sustainable trade.

### Existing recommendations for moving toward sustainability

A review of plant, fungus, and lichen harvesting and trade in Nepal (Smith‐Hall et al., 2020) showed little agreement on the way forward. Recommendations varied in focus (from technical interventions to political reform), nature (from liberalization to central control), scope (from individual species and locations to sector‐wide), spatial scale (from village to national levels), and direction (e.g., emphasis on cultivation vs. wild harvesting). Further, some recommendations were contradictory, for example, that local resource management should replace centralized resource monitoring and trade control (Larsen et al., 2005; Pyakurel et al., 2019) or the opposite (Ghimire et al., 2016). Also, unsubstantiated assumptions were commonly repeated and perpetuated across recommendations (Larsen & Olsen, 2007) that lacked grounding in sector realities (Smith‐Hall et al., 2020).

The most common type of policy intervention has been supply‐side restrictions, such as species‐level harvest bans (Appendix S1). An important supply‐side measure is cultivation. Although most species continue to be sourced from the wild (Goraya & Ved, 2017; Pyakurel et al., 2019), several lower‐ and middle‐elevation species have come into cultivation during the past 2 decades (e.g., *Swertia chirayita* [Cunningham, Brinckmann, Schippmann, et al., 2019] and *Cinnamomum tamala* [Choudhary et al., 2013]). In some places, cultivation is becoming the dominant mode of production (Pyakurel, 2020). However, incentive‐based supply‐side experiences also exist in Nepal, most notably with community management of forest resources (Paudel et al., 2022), a legal practice that can include nonforested areas such as alpine meadows.

Transactional interventions focusing on postharvest production network enforcement accompany supply‐side measures. These include national‐level mechanisms to monitor trade, including permits required to transport raw materials from the district of origin (Ministry of Forests and Environment, 2019). Voluntary certification schemes, such as the FairWild Standard, are rare in Nepal. The main international trade regulation mechanism is CITES; however, it does not document actual trade levels (Olsen, 2005b; Smith‐Hall et al., 2023). As elsewhere in the world, Nepal has no experience with demand‐side interventions to limit unsustainable consumption, such as bans or consumer‐oriented conservation campaigns.

## METHODS

### Theory of change

Across stakeholders with potentially conflicting narrow interests, a theory of change approach allows a structured identification and ranking of ways to promote a commonly desired change through mutual understanding of causal links connecting interventions to outputs and outcomes (Center for Theory of Change, 2024; Stein & Valters, 2012). This includes specification of actions and explicit formulation of the assumptions underlying each link in the model (Valters, 2015), here termed a roadmap. This enables the establishment of plausible causal chains between new interventions and expected impacts, hence providing a pragmatic outline of feasible transformational change. Arensman et al. (2018) distinguished 2 theory of change approaches: ex ante, focused on strategy development and planning, and ex post, focused on monitoring and evaluation.

Theories of change have been widely applied in international development activities (James, 2011; Valters, 2015). Further, based on stakeholder consultations, Biggs et al. (2016) developed an ex ante model to partner practitioners, donors, and policy makers with local communities to decrease pressure on wildlife from illegal trade. Roheim et al. (2018) used a literature review to examine how the theory of change for sustainable seafood has evolved over the past 2 decades, illustrating the importance of learning from experiences, a point also noted by Rice et al. (2020) in their work to develop robust conservation theory of change pathways. This may not be straightforward because bureaucracies and limited resources may privilege accountability over learning (Valters, 2014). Betts et al. (2020) used expert interviews to develop an ex ante theory of change focusing on causal pathways leading to positive International Union for Conservation of Nature Red List conservation outcomes. Martius et al. (2018) used definitions, United Nations decisions, and debates to illustrate the coexistence of different theories of change for reducing emissions from deforestation and forest degradation and enhancing forest carbon stocks in developing countries (REDD+) depending on the underlying rationales. We developed an ex ante application of theory of change to achieve biodiversity conservation, long‐term support to rural harvesters and processing industries, and a steady stream of public revenues.

The multisectoral nature of medicinal and aromatic plant, fungus, and lichen trade and conservation in Nepal (Smith‐Hall et al., 2020), the frequent occurrence of unsubstantiated assumptions (Larsen & Olsen, 2007; Madsen & Smith‐Hall, 2023), and the many actors involved and their diverse views and objectives (Fold et al., 2023; Larsen et al., 2005) mean a theory of change should be simple and thus accessible across disciplines and actors; transparent, making it clear what assumptions are made; integral, composed of logically coherent parts; and feasible, supported by an assessment of evidence for each element.

### Data generation

First, to achieve a structured understanding of the past, we characterized 50 years (1972–2022) of nominal legislative interventions in commercial medicinal and aromatic plant, fungus, and lichen trade in Nepal based on 4 dimensions (Appendix S1): type of intervention (supply side, transactional, demand side), governance approach (centralized, decentralized), choice of market intervention (incentives, restrictions), and intensity of implementation (low, high). Second, the advisory board of an international research project—consisting of representatives from government agencies, nongovernmental institutions, and the private sector—generated recommendations for the development of sustainable trade in Nepal at their meetings in 2016, 2017, and 2018 (Appendix S2). Wide stakeholder consultation was adopted to cover the broadest possible range of ideas and to limit political pressure to focus on certain outcomes (van Es & Gujit, 2015). This was supplemented with a survey of recommendations from participants at the International Conference on Wild Harvests, Governance and Livelihoods in Asia conducted in Kathmandu in 2017 (Appendix S3). These discussions and recommendations were synthesized to outline possible pathways to increased sustainability.

We then conducted a review of all publications related to medicinal plant trade and conservation in Nepal, taking the studies published up to 2015 from Smith‐Hall et al. (2020) and supplementing them with international peer‐reviewed studies published in 2016–2023 identified through the Web of Science. Applied search terms were “*Nepal and trade*” and “*plant* or *fungi* or *lichen*” or “*NTFPs*” (nontimber forest products). This yielded 61 references, of which 29 were included based on their titles and abstracts. The list of all consulted documents is in Appendix S4. From the literature, we extracted recommendations for improving sustainable management in Nepal, grouping these to produce a draft of the roadmap distinguishing actions, outputs, and outcomes, which was presented at a national workshop in Kathmandu in August 2023 with sector‐wide stakeholder representation (Appendix S5). Discussions took place in multistakeholder groups (harvesters, traders and wholesalers, processors, government, civil society, and researchers) to promote agreement between stakeholders and focused on validating the draft pathways and actions, identifying missing pathways and actions, and ranking actions. The workshop report is in Appendix S6. Workshop inputs were used to derive the final roadmap (Table 1). The prioritized actions were ordered logically (e.g., the increase cultivation pathway starts with actions related to cultivation and ends with marketing), and the wording was made more precise (e.g., the workshop action 1.1 research on agrotechnology for cultivation and efficacy became develop and disseminate agrotechnology for cultivation). Also, as groups developed similar actions, these were merged. In a few instances, actions were moved between pathways (e.g., pest risk assessment from increase international collaboration to improve sector governance). The literature review also identified the causal assumptions and theoretical explanations for each action and outcome (Appendices S7 & S8) and the positive feedback mechanisms between pathways (Appendix S9).

**TABLE 1:** Five pathways to increased sustainable management of commercial medicinal plants, fungi, and lichens in Nepal.

Pathway					
	1	2	3	4	5
	Increase cultivation	Strengthen local management	Support domestic businesses	Improve sector governance	Increase international collaboration
Actions	1.1 Develop and disseminate agrotechnology for cultivation, including through government support and establishing good agricultural practices	2.1 Hand over high‐elevation areas to local institutions	3.1 Support access to improved technology	4.1 Disseminate rules and knowledge, including harvesting guidelines for vulnerable species and need for pest risk assessment	5.1 Establish a dialogue mechanism for solving cross‐border trade issues
	1.2 Conduct market surveys and promotion campaigns for high‐value cultivated plant products	2.2 Support development and renewal of local management plans, including implementation of sustainable harvesting practices	3.2 Enhance infrastructure, including laboratories for product quality testing	4.2 Establish an economic incentive‐based approach to trade and conservation	5.2 Coordinate species protection measures across borders
		2.3 Support local conflict resolution, especially during wild harvesting	3.3 Support product diversification and value addition through private sector and government investments	4.3 Promote price dissemination and transparency	5.3 Review relevant importing countries’ and international policies and share knowledge
		2.4 Increase capacity for local‐level value addition	3.4 Create an enabling environment for doing business, for example, simplified tax procedures	4.4 Develop a decentralized system to collect credible official statistics along the value chain, including training of officials	5.4 Create an enabling environment for foreign direct investment and technology transfer
			3.5 Develop the domestic market and increase consumption	4.5 Develop medicinal plants curricula	
Outputs	1.3 Area and number of cultivated species with higher per unit production increased	2.5 The area under local management increased	3.6 Use of improved technology increased	4.6 Sustainable harvest guidelines used locally	5.5 International challenges discussed and solved and resolutions published
	1.4 District‐level extension support established	2.6 Varied local management practices exercised	3.7 Business supporting infrastructure enhanced	4.7 Revised nominal legislation enacted	5.6 Conservation of species across borders enhanced as per published resolutions
	1.5 Quality of medicinal plants maintained	2.7 Flexible guidelines for high‐elevation decentralized community management published	3.8 Investments, number of consumer products, and value‐addition increased	4.8 System for running price monitoring and dissemination established	5.7 Exports increased
	1.6 Sales of cultivated products increased	2.8 District‐level procedures for resolving conflicts established	3.9 Barriers to start and operate companies decreased	4.9 System for annual publication of valid trade statistics established	5.8 Foreign investments increased
		2.9 Provincial‐level value‐addition increased	3.10 Domestic market increased	4.10 Medicinal plant curricula developed and used	
Outcome	1.7 Less pressure on wild populations	2.10 Less harvesting in the wild	3.11 Replacing raw material export with domestic processing	4.11 More sustainable wild harvesting	5.9 Better international species protection
	1.8 Stable supply of higher quantities of high‐quality materials	2.11 Less habitat destruction and conversion	3.12 More competitive secondary processing industry	4.12 Fewer options for rent‐seeking	5.10 Increased market integration
	1.9 Higher producer incomes	2.12 Better resource management, monitoring, and control	3.13 Job creation, higher public revenues	4.13 Higher harvester net margins	5.11 More competitive export‐oriented secondary processing industry
		2.13 Improved local‐level conflict resolution		4.14 More transparent production network	
		2.14 Long‐term protection of harvester incomes		4.15 Better qualified nontrade stakeholders	
		2.15 Higher provincial‐level income			

## RESULTS

### The roadmap

Five pathways were identified for the roadmap: increase cultivation, strengthen local management, support domestic businesses, improve sector governance, and increase international collaboration. Each contains specific actions (from 2 to 5 per pathway) that lead to outputs (2‐5) and outcomes (2‐6). For instance, the “strengthen local management” pathway can be pursued through handing over high‐elevation areas to local communities (action), leading to an increased area under local management (output) and less overharvesting (outcome). For an overview of pathways, assumptions, and evidence, see Appendices S7 and S8. The 5 pathways are as follows:

1. The increase cultivation pathway supports cultivation to reduce unsustainable wild harvesting, increase producer income, and ensure an increased stable supply of high‐quality raw materials. This takes place through developing and disseminating cultivation techniques, conducting market surveys to uncover demand drivers, and running promotion campaigns for high‐value cultivated products.
2. The strengthen local management pathway emphasizes increased control of high‐elevation areas by nearby stakeholder communities to achieve improved resource management and conservation outcomes (less overharvesting and habitat destruction) while maintaining or increasing harvester incomes and public revenues. This is achieved through legally handing over high‐elevation areas, support to develop local management plans and implementation of sustainable harvesting practices, support to local conflict resolution, and a focus on local‐level value‐addition.
3. The support domestic businesses pathway develops a more competitive secondary processing industry, entailing less export of raw material, more in‐country value‐addition, more jobs, and higher public revenues. The emphasis is on improved use of technology supported by allied infrastructure investments while also working toward making it easier to do business and developing the domestic market (e.g., by focusing on Ayurvedic mass‐produced products and supporting Tibetan medicine production and distribution).
4. The improve sector governance pathway focuses on mechanisms to promote sustainable wild harvesting in more efficient and transparent markets with less rent‐seeking and higher harvester incomes. This requires disseminating existing knowledge of species‐specific sustainable harvest methods and transforming the current governance approach to a system of economic incentives promoting trade and conservation. This should include a simple and transparent legal basis for the handover of production areas without many technical constraints and improved collection and sharing of production network standard data, including prices in various nodes.
5. The increase international collaboration pathway pursues cross‐border species protection, increased market integration, and the development of a more competitive secondary processing industry in Nepal. To achieve this, a high‐level government dialogue mechanism is needed with India and China, the key product destinations. Relevant rules and procedures in importing countries should be synthesized and shared to facilitate exports. Facilitating foreign direct investment in processing companies and technology transfer is also important.

Many pathway actions are connected: packages of actions are likely to be more successful than individual actions. Feedback mechanisms (Appendix S9) allow the identification of bundles of mutually reinforcing actions, e.g., the pathway support domestic businesses should be implemented alongside cultivation techniques (action 1.1), increasing the handover of local resources (2.1), and supporting their management (2.2, 2.3) to prevent increased wild harvesting and less sustainable trade.

### Characteristics of past interventions versus roadmap actions

**FIGURE 1:**
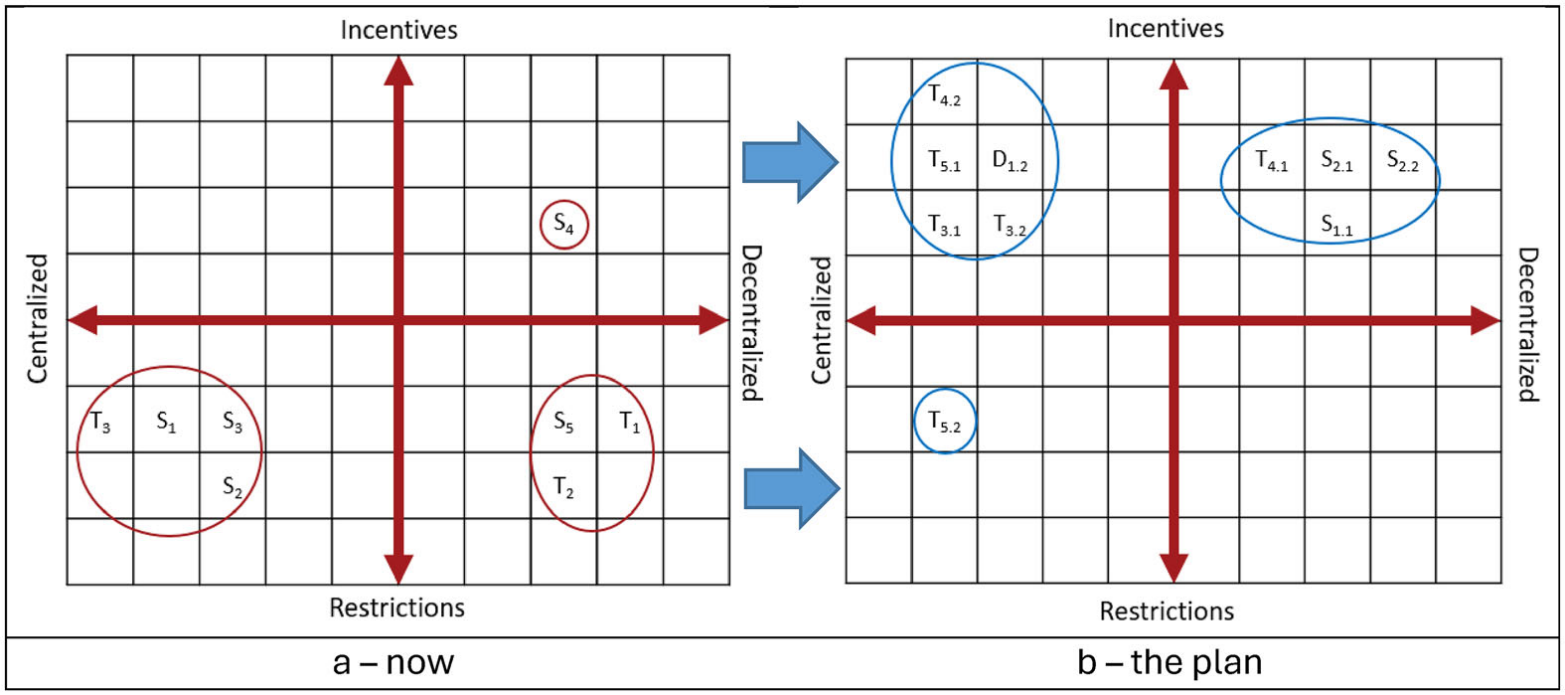
Governance (centralized, decentralized) and market intervention (incentives, restrictions) characterization of medicinal plant, fungus, and lichen harvest and trade interventions in Nepal (S, supply‐side; T, transactional; D, demand‐side): (a) example of high‐degree implementation (Appendix S1) (S_1_, nation‐wide bans on harvesting [e.g., for the orchid *Dactylorhiza hatagirea*]; S_2_, all harvests must be as per divisional forest strategic plans; S_3_, royalty payments; S_4_, transfer of resource base units [typically forests] for approved local management under community forestry legislation; S_5_, resource access restrictions in protected areas; T_1_, permits for transport out of district of origin and for export; T_2_, taxes on trade [e.g., to the District Development Committee]; T_3_, ban on export of unprocessed raw material [e.g., rhizomes of *Nardostachys jatamansi*]) and (b) examples of interventions (subscripts, action numbers in Table 1; S_1.1_, develop and disseminate agrotechnology; D_1.2_, conduct market surveys and promotion campaigns; S_2.1_, hand over high‐elevation areas; S_2.2_, support local management plans; T_3.1_, support access to improved technology; T_3.2_, enhance infrastructure; T_4.1_, disseminate rules and knowledge; T_4.2_, establish an economic incentive‐based approach to trade and conservation; T_5.1_, establish a cross‐border dialogue mechanism; S_5.2_, coordinate species protection measures across borders).

The past 50 years’ interventions in the trade of medicinal and aromatic plants, fungi, and lichens (Appendix S1) included 61 interventions—found in 37 acts, regulations, strategies, policies, and guidelines—dominated by centralized approaches based on incentives with low degrees of implementation. Many interventions were repeated, for example, proposals to develop secondary processing industries were made 13 times from 1988 to 2020. Zooming in on past interventions with a high degree of implementation and mapping these with the governance and market dimensions (Figure 1a) showed that these were characterized by centralized and decentralized restrictions (interventions clustered in the quadrants below the *x*‐axis), except for decentralized management (upper right quadrant). The actions proposed in the roadmap, in contrast, emphasize a mix of centralized and decentralized incentives (Figure 1b, interventions clustered above the *x*‐axis).

## DISCUSSION

Even in the context of hundreds of traded renewable environmental products (Smith‐Hall & Chamberlain, 2023), where prices and demand are expected to increase for many commodities and species‐level knowledge on sustainable harvesting practices is inadequate for most species, the roadmap shows that it is possible to achieve stakeholder‐wide agreement on prioritized actions. Thus, the roadmap constitutes a coherent vision and provides specific actions and pathways to move toward sustainable trade. This is relevant in many other contexts. Currently, Asian medical industries across a range of traditional medicine systems, including in China, India, Japan, South Korea, and Mongolia, are undergoing an industrial revolution—characterized by increased industrialization, commercialization, and globalization while continuing to rely on renewable environmental products as inputs (Kloos, 2022). This creates tensions addressed in the roadmap, including the problems of continued supplies of wild‐harvested raw materials, the need for increased cultivation, and the role of the state in regulating the industries (Blaikie, 2022). Indeed, the challenges of continued industrial expansion, ecological sustainability concerns, and inclusive socioeconomic development are found in many sectors and locations in the Global South. Examples include the shea (*Vitellaria paradoxa*) nut industry in West Africa (Wardell et al., 2021), argan (*Sideroxylon spinosum*) oil in Morocco (Perry, 2020), the açai palm (*Euterpe oleracea*) fruit in the Amazon (Diniz & Cialdella, 2023), the agarwood (*Aquilaria* spp.) oil from Southeast Asia (López‐Sampson & Page, 2018), and bushmeat trade (Ingram et al., 2021). Despite the widespread and increasing economic importance, studies on these products usually ignore enterprises and secondary processing. This includes the recent Intergovernmental Science–Policy Platform on Biodiversity and Ecosystem Services global review of the sustainable use of wild species (IPBES, 2022). The roadmap exemplifies how tension around commercially traded biodiversity‐derived products can be approached in the pursuit of increased sustainability.

No structured review of pathway implementation outcomes exists outside the region (Appendices S7 & S8). However, regarding increased cultivation (Pathway 1), understanding of the underlying dynamics is good, allowing for design of species‐level interventions (Madsen & Smith‐Hall, 2023). On local management (Pathway 2), a strong body of evidence supports that it enhances positive environmental and income‐related outcomes; however, attention must be paid to ensuring access and rights to resources (Hajjar et al., 2021). There are many pitfalls (Cunningham, 2011) when supporting domestic businesses (Pathway 3) but also examples of success, such as for Tibetan medicine in China (Saxer, 2013). On improving sector governance (Pathway 4), a recent global synthesis warns against a tendency to focus on short‐term economic gain at the cost of sustainability (Kleinschmit et al., 2024), emphasizing the importance of implementing mutually enforcing pathways (Appendix S9). Successes and failures of cross‐border (Pathway 5) conservation collaboration are plentiful (Kark et al., 2015), although experiences with promoting sustainable trade appear limited.

For Nepal, in a wider sense, the roadmap suggests a green, resilient, and inclusive development path for environmental product trade that can help the country escape its current low‐growth trap (World Bank, 2022) through a shift from producing lower value domestic consumer products (Caporale et al., 2020) to a sustainable export of higher value commodities. It also identifies the major risks (Appendices S7–S9). As such, the roadmap may help garner extrasectoral support for needed investments. However, the roadmap is not a silver bullet. The pathways identify directions but need further specification by stakeholders. For instance, action 1.1 to develop cultivation techniques needs species selection criteria, geographical focus, and decisions on how technology should be developed (e.g., through centralized trials or farmer experimentation). Using the roadmap thus requires further sector stakeholder collaboration to design implementation plans for individual and bundled actions. This is further discussed for each pathway below. Also, the difficulty of implementation will vary across actions. Creating medicinal plant curricula (action 4.5), and integrating these into university teaching at institutes of forestry and botany in Nepal, is straightforward compared with establishing an economic incentive‐based approach to trade and conservation (action 4.2).

Past centralized and decentralized restrictions (Figure 1a) have not succeeded (Pyakurel et al., 2018, 2019). They merely modified the production network, such as the location of traders in response to the expanding rural road network (Fold et al., 2023), rather than influencing the degree of sustainable harvesting and trade. A new approach is required that emphasizes a mix of centralized and decentralized incentives (Figure 1b). This raised the question: why should the roadmap succeed where previous attempts have failed? Four factors support this. First, the roadmap is more integrated and comprehensive than previous interventions. It builds on multisectoral (rural development, forestry, infrastructure, protected areas, and others) experiences (e.g., the action to hand over high‐elevation production areas to local institutions can build on the positive experiences from community forestry) (Paudel et al., 2022); includes national and regional challenges (e.g., legislative interventions in India impact the medicinal plant production network in Nepal [Fold et al., 2023]); and enhances the inclusion of main stakeholder groups (private sector, civil society, local communities, and government agencies) to garner wide and sustained support (Appendices S2, S3, S5, & S6). Second, the roadmap includes new intervention types, not only the restrictions of the past (Figure 1), thus countering the criticism of ignoring other types of public policy recommendations (Smith‐Hall et al., 2020), which could strengthen stakeholder support. Third, the roadmap does not require new but refers to existing institutions, such as the divisional forest offices with their fine‐grained local presence, and builds on scientific evidence (Appendices S7 & S8). Fourth, the decentralized natural resource management paradigm is widely accepted in Nepal (Ojha & Hall, 2023) and supports actions such as the handover of high‐elevation pasture areas.

The main challenges to roadmap success are 3‐fold. First, although the roadmap acknowledges recent advances in understanding how environmental product conservation and trade are unofficially regulated and practiced in Nepal (Adhikari, 2017; Baral et al., 2018; Basnyat et al., 2019, 2018) and the importance of feeding this into the implementation plans for actions to promote feasible interventions (see next paragraph), the existing political ecology of the production network constitutes a challenge. Arguably, the rent extraction related to commercial medicinal plants, fungi, and lichens may lessen as decentralization increases provincial budgets (World Bank, 2023a), making other sectors (e.g., infrastructure development) more financially attractive. Second, funding for roadmap actions is limited and unlikely to be substantially increased in the near future (e.g., continuously low budgets for renewing community forestry operation plans [Baral et al., 2018]). This reinforces the importance of keeping required management plans simple and low cost and making decentralized management economically attractive to local communities and emphasizes the need for private sector (domestic and international) investments in developing the secondary processing industry. Third, Nepal's history of central decision‐making (Ojha et al., 2007; Whelpton, 2005) and the preference for restrictions rather than incentives (Figure 1a) mean there is limited tradition for inclusive incentive‐based participatory development of supralocal initiatives to enhance sustainable trade, including building on science inputs, though there are steps taken in this direction (Ojha et al., 2020).

We considered how to operationalize the pathways (Appendices S1 & S7–S9). For Pathway 1, increase cultivation, wild harvested supplies may decrease due to a lack of labor. In 2021, 7.5% of the entire population lived abroad (NSO, 2021), and remittances constituted 29.1% of the country's GDP in 2022 (World Bank, 2023b). High‐elevation areas experience depopulation due to international and domestic migration (Childs et al., 2014). This supports the urgency of engaging more widely with cultivation, focusing on native species. The outmigration causes deagrarianization in the hills and mountains (Ojha et al., 2022; World Bank, 2023b), freeing land for cultivation. Likewise, the length of the national road network tripled between 1998 and 2018 (DoR, 2018), decreasing transport costs. Four approaches are important: uncovering and obtaining existing cultivation technology, making government investments in high‐value species, gearing district‐level extension services toward supporting farmer‐initiated cultivation experiments, and focusing on areas with less outmigration and more labor, notably the important sourcing areas in western Nepal (Pyakurel et al., 2018).

For Pathway 2, strengthen local management, most vulnerable and high‐value species are found at high elevations (Pyakurel et al., 2019), where medicinal plant income is also most important to rural households (Olsen & Larsen, 2003; Pouliot et al., 2018; Timmermann & Smith‐Hall, 2019), emphasizing the importance of handing over management authority. Experiences from community forestry predict positive conservation outcomes (Paudel et al., 2022). However, overly technical and bureaucratically enforced management plans may enforce de facto underharvesting to the detriment of collecting households (Baral et al., 2018; Meilby et al., 2014). Handover can be done to democratically elected rural municipalities or community forestry groups, paying particular attention to avoiding excluding legitimate users such as low‐caste households. Handover procedures should be simple, as should management plan requirements. Experiences from community forestry show that technical requirements have facilitated rent extraction and recentralization of management authority (Baral et al., 2018; Basnyat et al., 2018, 2019). Instead, management can be designed using species‐level harvesting guidelines, translated into the amount that can be harvested in a specific location at a specific time at specified intervals (Ghimire et al., 2008; Larsen, 2005). New harvesting guidelines should focus on vulnerable species (Pyakurel et al., 2019). Other species can be left unregulated unless prices increase substantially, such as more than 50% (Smith‐Hall et al., 2023).

For Pathway 3, support domestic businesses, there is an opportunity to transition from raw material export to secondary processing. The need for access to improved technology and enhanced infrastructure is well documented (Caporale et al., 2020). The government should prioritize technology transfer from neighboring countries and support the establishment of accredited laboratories. Access to these could increase the competitiveness of secondary processing industries, allowing them to invest in product development and diversification with knock‐on effects on domestic consumption. This would be supported by sector‐level initiatives to cut costs, including feedback from other pathways, such as more transparent prices and cheaper cultivated products. General constraints on doing business should be addressed as part of a wider reform of the agribusiness sector (World Bank, 2018).

For Pathway 4, improve sector governance, much can be gained by collecting and sharing what is already available, including prices and sustainable harvesting methods, such as the regenerative cultivating‐while‐collecting technique for *Nardostachys jatamansi* (Larsen, 2005). The main challenge is designing and implementing an economic incentive‐based approach to trade and conservation at both national and provincial levels. This requires sustained involvement of key stakeholders willing to collaborate on developing new approaches to regulate harvest and trade. Examples include the development of subsidies for sustainability control (e.g., grants, low‐interest loans, or favorable tax treatment rewarding actors establishing sustainable harvesting), a sustainability credit trading system (e.g., cap‐and‐trade that sets a maximum allowable harvest for vulnerable species and allows selling of harvesting rights), or a voluntary sustainability action program for the industry (e.g., participating companies documenting sustainable sourcing of vulnerable species gain subsidized access to technology).

For Pathway 5, increase international collaboration, the focus is on establishing a dialogue mechanism to address trade and conservation issues with India and China (Larsen & Olsen, 2007). Examples include cross‐border trade barriers, such as the Plant Quarantine (Regulation of Import into India) Order (Ministry of Agriculture, 2003) in India that drives trade underground (Fold et al., 2023), and coordinating species protection to avoid leakage (e.g., India has banned commercial collection of *N. jatamansi* rhizomes [Chauhan, 2021], but it remains the major market for the harvest from Nepal [Olsen, 2005b; Smith‐Hall et al., 2023]). The mechanism could also facilitate access to existing technology (e.g., the cultivation of *Paris polyphylla* is almost nonexistent in Nepal but widespread in China [Cunningham et al., 2018]).

The roadmap shows it is possible to achieve stakeholder‐wide agreement on prioritized actions to enhance the sustainability of environmental product trade at the national level, even in a context of hundreds of traded products and species‐level data deficiency. Although the identified pathways are familiar, the strength is in their prioritized selection and awareness of assumptions and how pathways can reinforce each other. The roadmap is an example of a refocused, better integrated, and regionally coordinated approach to promote sustainable harvest and trade. The approach emphasizes economic incentives, decentralization of resource management, and more simplicity and greater transparency while establishing a better foundation for generating valid and reliable trade data and predicting future demand. This should lead to increased sustainability of commercial harvesting while maintaining or increasing biodiversity and rural household and government incomes. The roadmap is designed to inform policy makers and decision makers at all levels and includes specifications of challenges and suggestions to operationalize the proposed actions. We hope this work and the roadmap will provide the impetus for stakeholders to coordinate actions to the benefit of species, actors, the industry, and the country and inspire similar efforts to increase sustainability in the huge array of environmental products traded in and between countries in other parts of the world.

## Supporting information



Supporting Information
