# Supplementary material for: A roadmap to sustainable management of commercial medicinal and aromatic plants, fungi, and lichens in Nepal
Source: Conserv Biol. 2025 Jan 17;39(4):e14442. doi: 10.1111/cobi.14442 (PMC12309637; doi:10.1111/cobi.14442)
Supplement: Supplementary file 1 — Supporting Information [file COBI-39-e14442-s001.docx]

**Supporting Information**

A roadmap to sustainable management of commercial medicinal and aromatic plants, fungi, and lichens in Nepal

*Carsten Smith-Hall, Dipesh Pyakurel, Thorsten Treue, Mariève Pouliot, Suresh Ghimire, Anastasiya Timoshyna, and Henrik Meilby*

An overview and characterization of nominal legislative interventions in commercial medicinal plants traded in and from Nepal are presented in Appendix S1 (forming the basis for Fig. 1 in the main text of the paper). The list of stakeholders, more than 50 people from more than 20 institutions, who provided inputs to the draft roadmap at the annual meetings in the Advisory Board to the Transiting to Green Growth: Natural Resources in Nepal (TGG-N) research project is provided in Appendix S2. An overview of the recommendations for interventions to enhance sustainable medicinal plant management, harvesting, trade, and enterprises in Nepal given anonymously by participants in the International Conference on Wild Harvests, Governance and Livelihoods in Asia, in 2017 in Kathmandu is presented in Appendix S3. The list of stakeholder representatives (n=29) who provided inputs to the draft roadmap at the “Building a roadmap for sustainable management of commercial medicinal plants in Nepal” workshop, Kathmandu, 24 August 2023, constitutes Appendix S4. The report from this workshop is provided in Appendix S5. The list of assumptions for each roadmap pathway action is found in Appendix S6, for each outcome in Appendix S7, while Appendix S8 provides an overview of positive feedback loops between pathways.

**Table of content**

**Appendix S1** A 50-year chronological overview (1972 to 2022) and characterization of nominal legislative interventions in commercial medicinal plants trade in and from Nepal 3

**Appendix S2** List of stakeholders who provided roadmap inputs at the 2016, 2017, and 2018 annual meetings in the Advisory Board to the Transiting to Green Growth: Natural Resources in Nepal (TGG-N) research project 13

**Appendix S3** An overview of recommendations for interventions to enhance sustainable medicinal plants management, harvesting, trade and enterprises in Nepal given anonymously by participants in the International Conference on Wild Harvests, Governance and Livelihoods in Asia, 30 November to 2 December 2017, Kathmandu 14

**Appendix S4** List of consulted documents for the development of the roadmap 16

**Appendix S5** List of stakeholder representatives (n=29) who provided inputs to the draft roadmap at the “Building a roadmap for sustainable management of commercial medicinal plants in Nepal” workshop, Kathmandu, 24 August 2023 37

**Appendix S6** Abridged workshop report: Building a roadmap for sustainable management of commercial medicinal plants in Nepal 38

**Appendix S7** List of pathway action assumptions for the roadmap to sustainable management of commercial medicinal and aromatic plants, fungi, and lichens in Nepal 51

**Appendix S8** List of pathway outcome assumptions for the roadmap to sustainable management of commercial medicinal and aromatic plants, fungi, and lichens in Nepal 57

**Appendix S9** Positive feedback loops (reinforcing mechanisms) between roadmap pathways 62

**References** 63

Appendix S1 A 50-year chronological overview (1972 to 2022) and characterization of nominal legislative^1^ interventions in commercial medicinal plants trade in and from Nepal according to: type^2^ (supply-side, transactional, demand-side), governance approach (centralized vs decentralized), choice of institutional arrangement (pull vs push: incentives for changed behavior vs punishments for breaking restrictions)^3^, and degree of implementation (low, high)^4^

The analysis in the table below is focused on commercial medicinal plants, not other environmental products or wider natural resource management issues or experiences.

| **Intervention** | **Intervention type** | **Governance approach** | **Institutional arrangement** | **Degree of implementation** | **References**^5^ |
| --- | --- | --- | --- | --- | --- |
| Amendments in national, community, public, and private forest management plans to increase medicinal plant production | Supply-side | Centralized | Pull | Low | Fifteenth Plan: 2019/20-2023/24 (2020) |
| Pocket production area identification, development of cultivation techniques, and establishment of processing centers | Supply-side | Centralized | Pull | Low |  |
| Commercialization and export of medicinal plant extracts and processed products | Transactional | Centralized | Pull | Low |  |
| Establishment of at least one medicinal plants-based medium or large enterprise in each province | Transactional | Centralized | Pull | Low |  |
| Research on native medicinal plants | Supply-side | Centralized | Pull | Low |  |
| Adding commercial medicinal plant species, amending the royalty list for some medicinal plant species | Supply-side | Centralized | Pull | High | Forest Regulation 2079 (2022) |
| Amending the process of quota allocation (enabling bids for collection permits) | Supply-side | Centralized | Pull | High |  |
| Support the "Made in Nepal" campaign through processing, value addition, and high-value medicinal plant commercialization | Transactional | Centralized | Pull | Low | National Forest Policy 2075 (2019) |
| Promote and expand medicinal plants-based green enterprises | Transactional | Centralized | Pull | Low |  |
| Promote medicinal plant cultivation, collection, conservation, processing, certification, commercialization, and export to contribute to national prosperity | Supply-side,  Transactional | Centralized | Pull | Low |  |
| Integrate traditional medicinal plant knowledge with science to develop new knowledge | Transactional | Centralized | Pull | Low |  |
| Encourages private sector engagement in cultivation, value addition, and export | Supply-side, Transactional | Centralized | Pull | Low |  |
| Allocate grants (up to NPR^6^ 1.5 million) for commercial cultivation and enterprise development of medicinal plants through the Department of Plant Resources to reduce overharvesting in the wild and create rural employment and income | Supply-side, Transactional | Centralized | Pull | Low | Grant allocation procedure for development of medicinal plants 2075 (2019) |
| Allocate grants (up to NPR 2 million per enterprise) to forest-based enterprises for development and expansion, e.g. to purchase equipment and technology | Transactional | Centralized | Pull | Low | Grant allocation procedure for promotion and development of forest based enterprises 2075 (2019) |
| Forest-based Cottage and Small-Scale Enterprises do not have to inform the Division Forest Office if they use forest products as raw materials | Transactional | Centralized | Pull | High | Forest Act 2076 (2019) |
| Adding medicinal plant species to the royalty list, some rates increased | Supply-side | Centralized | Pull (more species can be legally traded)  Push (higher royalties for some species) | High | Nepal Gazette (68-34-3) 2075 (2018) |
| Relaxing rules for 13 NTFP species regarding harvest and transport | Supply-side | Centralized | Pull | High | Forest Investment Program 2017 |
| Promote medicinal plant-based enterprises for livelihood and economic development with a strong role of the private sector | Transactional | Centralized | Pull | Low |  |
| Prohibition on trade or transaction of threatened or vulnerable wild flora | Transactional | Centralized | Push | High | Act to Regulate and Control International Trade in Endangered Wild Fauna and Flora, 2017 |
| Double the annual export value of non-timber forest products / medicinal plants from NRs 6 billion to NRs 12 billion by 2025 through   - Expansion of cultivation inside and outside forests - Sustainable management of high-value medicinal plants - Supporting quality assurance and certification systems - Enhancing export markets - Encourage private sector investment in cultivation and enterprise development | Supply-side,  Transactional | Centralized | Pull | Low | Forestry Sector Strategy 2016-2025 (2016) |
| Amendment of royalty payments (adding 29 species, changing royalties for 28 species) | Supply-side | Centralized | Pull, Push | High | Nepal Gazette (65-26-3) 2072 (2015) |
| Identifies opportunities in the forestry sector to produce processed products for domestic and export markets | Transactional | Centralized | Pull | Low | Nepal REDD+ Strategy Part 1: Operational Strategy 2015 |
| Prioritize the development and implementation of medicinal plant management plans by all DFOs and CFUGs, targeting conservation and over-harvesting of threatened species | Supply-side | Centralized | Pull | Low | Nepal National Biodiversity Strategy and Action Plan 2014-2020 |
| Promote medicinal plant-based micro-enterprises to enhance local livelihoods and income | Transactional | Centralized | Pull | Low |  |
| Promote resource inventories for sustainable medicinal plant harvesting | Supply-side | Centralized | Pull | Low | NTFPs resource inventory guideline 2069 (2012) |
| Ban on collection, transport, and trade of all lichens | Supply-side | Centralized | Push | High | Nepal Gazette (60-38-5) 2067 (2011) |
| Compulsory to obtain a phytosanitary certificate from a Plant Quarantine Office before export | Transactional | Centralized | Pull (facilitates export) | High | Plant Protection Rules 2010 |
| Establish commercial cultivation and value-addition for trade and export at a mass scale for economic development | Supply-side, Transactional | Centralized | Pull | Low | Industrial Policy 2067 (2011) |
| Prevention of the introduction, establishment, prevalence, and spread of pests while importing or exporting medicinal plants | Transactional | Centralized | Pull (facilitates export)  Push (hinders trade) | High | Plant Protection Act 2007 |
| Domesticate medicinal plants and promote value-added processing (2006-2010), including through biotechnology and bio-prospecting | Supply-side, Transactional | Centralized | Pull | Low | Nepal Biodiversity Strategy Implementation Plan 2006 |
| Promote district-level medicinal plant-based enterprises based on local forest and land management systems, farmers’ socio-economic conditions, and technically viable production systems | Transactional | Centralized | Pull | Low | Guidelines for NTFP Based Enterprise 2005 |
| Prioritize medicinal plants and focus on enterprises with high economic potential | Transactional | Centralized | Pull | Low |  |
| Promote inventories of commercial medicinal plants and conduct studies on sustained yield and marketing | Supply-side | Centralized | Pull | Low | Nepal National Action Programme on Land Degradation and Desertification 2004 |
| Encourage plantations of medicinal and aromatic plants | Supply-side | Centralized | Pull | Low |  |
| Identify low- and high-value products, develop cultivation, harvesting, and processing technologies | Supply-side | Centralized | Pull | Low |  |
| Promote medicinal plant cultivation in private and public lands, their sustainable utilization in community and leasehold forests, and operation of processing facilities | Supply-side | Centralized | Pull | Low |  |
| Establishing Nepal as a storehouse of medicinal plants by 2020 through holistic sector development and utilization and conservation of high-value medicinal plants | Supply-side, transactional | Centralized | Pull | Low | Herbs and NTFPs development policy 2061 (2004) |
| Promote (i) production and conservation, (ii) private sector processing, (iii) business development services, (iv) inclusion of Disadvantaged Groups, and (v) export | Supply-side, transactional | Centralized | Pull | Low |  |
| Undertake a baseline survey of medicinal plants for better understanding and management | Supply-side | Centralized | Pull | Low | Nepal Biodiversity Strategy 2002 |
| Provides strategies for medicinal plant promotion, equitable benefit sharing, and technical knowledge for sustainable harvesting | Supply-side | Centralized | Pull | Low |  |
| Bans on collection, trade, and transport of panchaaunle^7^ and bark of okhar, and permits for collection and trade of kutki | Supply-side | Centralized | Push | High | Nepal Gazette (51-36-3) 2058 (2001) |
| Recognize the role of the private sector in the development of medicinal plant resources | Supply-side, transactional | Centralized | Pull | Low | Forestry Sector Policy 2000 |
| Provide livelihood opportunities to poor and landless people in medicinal plant harvesting for industries | Supply-side,  transactional | Centralized | Pull | Low |  |
| Eliminate restrictions on internal trade and transport of medicinal plant products, and conduct resource and market surveys | Transactional | Centralized | Pull | Low |  |
| Develop production and processing of medicinal plants, discourage export of unprocessed products, encourage export of high value-added products, and promote commercialization | Transactional | Centralized | Pull | Low |  |
| District Development Committees (DDC) can tax medicinal plants produced within the district (other DDCs cannot) | Transactional | Centralized | Push | High | Local self-governance act 1999 and Local self-governance regulation 2000 |
| Requires Initial Environmental Examination (IEE) of an area if collection of more than 5 tons of medicinal plants, and Environmental Impact Assessment (EIA) if more than 50 tons | Supply-side | Centralized | Push | High | Environment Protection Act, 1996 |
| Conservation Area Management Committees can issue medicinal plant collection permits against fees | Supply-side | Centralized | Pull (easier to obtain than from DFOs) | High | Conservation Area Management Rules 2053, 1996 |
| Utilization of lesser-known species (1993). The action plan (1998) emphasized (i) solving collection and marketing problems, (ii) cultivation, and (iii) establishing a medicinal plant-based industry | Supply-side,  transactional | Centralized | Pull | Low | Nepal Environmental Policy and Action Plan (NEPAP) 1993 & 1998 |
| Legitimatize the sustainable management and trade of the selected medicinal plant products | Supply-side | Centralized | Pull | Low | Forest Act 1993 and Forest Regulations 1995 (amendment 2001) |
| DFO and CFUG will issue collection permits for government and community forests, respectively. Transit and export permits will be issued from DFO | Supply-side, transactional | Centralized | Push | High |  |
| Legal protection to 17 plant species, lichen, and shilajit; ban on panchaaunle and kutki; ban on export in crude form for eight species | Supply-side | Centralized | Push | High |  |
| Made provision for IEE for collecting and processing orchids, lichens, and other medicinal plants | Supply-side | Centralized | Push | Low | National Environmental Impact Assessment Guidelines 1993 |
| Preferential access (duty-free and quota-free) of medicinal plants from Nepal to India | Transactional | Centralized | Pull | Low | Trade and Transit Agreement, 1992 |
| NTFPs constitute one of the six forestry programs. Commercial medicinal and aromatic plant selection for promotion | Supply-side,  transactional | Centralized | Pull | Low | Master Plan for Forestry Sector 1988 |
| Increase the supply and processing of medicinal plants | Supply-side,  transactional | Centralized | Pull | Low |  |
| Establish commercial cultivation, agroforestry, and cultivation in community forests | Supply-side | Centralized | Pull | Low |  |
| Establish appropriate policies, regulations, and management approaches to ensure sustainable extraction of medicinal plants | Supply-side | Centralized | Pull | Low | National Conservation Strategy (1988) |
| Recognize the need for improved management of medicinal plants | Supply-side | Centralized | Pull | Low | National Forest Policy 1976 |
| Conservation of medicinal plants through limiting harvesting in certain types of protected areas | Supply-side | Centralized | Push | High | National Parks and Wildlife Conservation Act, 1973 |
| A phytosanitary certificate is required for import and export of all medicinal plant products | Transactional | Centralized | Pull  Push | High | Plant Protection Act 1972 |

^1^ Nominal legislation refers to laws and regulations specifically including aspects of medicinal plant management, harvest, and/or trade.
^2^ We define intervention types following Sas-Rolfes et al. (2019): supply-side measures directly address onsite production and harvesting conditions and activities, transactional measures target production network activities between harvesting and final consumption, and demand-side measures aim at consumers.
^3^ We characterize centralized governance as decisions imposed on actors from the outside (e.g. requirement for collection permits) and decentralized as decisions imposed by actors on themselves (e.g. a user group banning the collection of a product in their communal area). We characterize *pull* as a positive incentive factor (e.g. subsidies) and *push* as a negative factor (e.g. fines and jail sentences).

^4^ We distinguish a low degree of implementation, such as the lack of implementation of the Herbs and Non-Timber Forest Product Development Policy from 2004, and a high, indicating influence on the day-to-day trade of medicinal plants. Note that high does not equal effective, e.g. the ban on the export of unprocessed *N. jatamansi* rhizomes has not hindered a boom in such export in past decades (Smith-Hall et al., 2023); in this case, high means implementation of the intervention, e.g. through physical inspection of trucks during transport, without achieving the official goal of stopping the export. These qualitative low/high assessments are derived from Smith-Hall et al. (2020) and the actor interviews specified in Smith-Hall et al. (2018).

^5^ References refer to the sources of the intervention. For bibliographic details of each intervention, see the relevant entry in the list of GoN and HMGN publications in the list of references at the end of these Supporting Information files.

^6^ Nepal rupees. The exchange rate was 133 NPR/USD in October 2023.

^7^ Panchaaunle is *Dactylorhiza hatagirea*, okhar is *Juglans regia*, and kutki is *Neopicrorhiza scrophulariiflora* (Pyakurel et al., 2019).

# Appendix S2 List of stakeholders who provided roadmap inputs at the 2016, 2017, and 2018 annual meetings in the Advisory Board to the Transiting to Green Growth: Natural Resources in Nepal (TGG-N) research project

| *3rd TGG-N Advisory Board meeting in Kathmandu on 16th December 2016* |
| --- |
| Pradip Maharjan (Agro Enterprise Centre/Federation of Nepalese Chamber of Commerce and Industry); Rishi Ram Tripathi (Nepal Foresters’ Association); Rose Shrestha (Department of Plant Resources); Jay Prakash Dutta, Shiv Chandra Dhakal, Dipesh Pyakurel, and Shakuntala Thapa (Agriculture and Forestry University); Sahas Man Shrestha (Department of Forest Research and Survey); Suresh Ghimire, Chitra Baniya, Deep Jyoti Chapagain, Mukti Ram Poudel, and Bharat Babu Shrestha (Central Department of Botany, Tribhuvan University); Ganesh Karki, Bhim Prakash Khadka, Thakur Bhandari, Krishna Ojha, Prabata Gautam, Brikha Bahadur Shahi, Suvash Devkota, Ram Poudel, Omi Gurung, Rama Aryal, Krishna Chhetri, Surya Tamang, and Chandra Tamang (Federation of Community Forestry Users, Nepal (FECOFUN)); Abhoy Kumar Das, Arjun Chapagain, and Carsten Smith-Hall (Transiting to Green Growth: Natural Resources in Nepal (TGG-N)); Debendra Prasad Dahal (Herbal Entrepreneurs Association of Nepal); Santosh Rayamajhi (Institute of Forestry, Tribhuvan University); Anuj Raj Sharma (Community Forest Division, Department of Forests); Rajan Prasad Paudel (National Trust for Nature Conservation); Laxmi Datt Pant (Herbs Production and Processing Company Limited); Pashupati Koirala (Adaptation for Smallholders in Hilly Areas (ASHA) Project); and Naya Sharma Paudel (ForestAction Nepal) |
| *4th TGG-N Advisory Board meeting in Kathmandu on 4th December 2017* |
| Indira Bhattarai, Shiva Chandra Dhakal, Dipesh Pyakurel, and Gandhiv Kafle (Agriculture and Forestry University); Sahas Man Shrestha (Department of Forest Research and Survey); Suresh Ghimire, Deep Jyoti Chapagain, Mukti Poudel, Chitra Baniya, and Bharat Babu Shrestha (Central Department of Botany, Tribhuvan University); Ganesh Karki (Federation of Community Forestry Users, Nepal (FECOFUN)); Abhoy Kumar Das, Arjun Chapagain, and Carsten Smith-Hall (Transiting to Green Growth: Natural Resources in Nepal (TGG-N)); Devendra Dhakal (Herbal Entrepreneurs Association of Nepal); Prem Prasad Sharma (Department of Forests); Laxmi Dutt Pant (Herbs Production and Processing Company Limited); Pashupati Koirala and Swoyambhu Man Amatya (Adaptation for Smallholders in Hilly Areas (ASHA) Project); Pradip Maharjan and Utsab Thapa (Agro Enterprise Centre/Federation of Nepalese Chamber of Commerce and Industry); Govinda Ghimire (Nepal Herbs and Herbal Products Association); Krishna Bhujel (Green Solution Nepal); Rajendra Basnet (Ayurvedic Medicine Producers Association, Nepal (AMPAN)); and Pushpa Ghimire (Asia Network for Sustainable Bioresources (ANSAB)) |
| *5th TGG-N Advisory Board meeting in Kathmandu on 3rd November 2018* |
| Pradip Maharjan (Agro Enterprise Centre/Federation of Nepalese Chamber of Commerce and Industry); Anuj Sharma (Department of Forests); Sahas Man Shrestha and Buddhi Poudel (Department of Forest Research and Survey/ Forest Research and Training Centre); Naba Raj Devkota, Shiva Chandra Dhakal, Dipesh Pyakurel, and Gandhiv Kafle (Agriculture and Forestry University); Suresh Ghimire, Deep Jyoti Chapagain, and Mukti Poudel (Central Department of Botany, Tribhuvan University); Thakur Bhandari, Parbata Gautam, and Birkha Shahi (Federation of Community Forestry Users, Nepal (FECOFUN)); Abhoy Kumar Das, Carsten Smith Hall, and Thorsten Treue (Transiting to Green Growth: Natural Resources in Nepal (TGG-N)); Jagadish Chandra Ghimire (Herbal Entrepreneurs Association of Nepal); Pashupati Koirala and Swoyambhu Man Amatya (Adaptation for Smallholders in Hilly Areas (ASHA) Project); Govinda Ghimire and Prem Raj Tiwari (Nepal Herbs and Herbal Products Association); Keshav Prasad Khanal (The Mountain Institute); and Arun Sharma Poudel (Kathmandu Forestry College) |

Appendix S3 An overview of recommendations for interventions to enhance sustainable medicinal plants management, harvesting, trade and enterprises in Nepal given anonymously by participants in the International Conference on Wild Harvests, Governance and Livelihoods in Asia, 30 November to 2 December 2017, Kathmandu (for list of participants, see Das et al., 2018)

These recommendations were received anonymously (n=22 individual submissions) through a Survey Monkey online questionnaire. Recommendations are here reproduced as they were received (but writing out acronyms, minor edits for clarification and leaving out duplicates and recommendations that did not relate to commercial medicinal plants). Here, we group them under four headings (management, harvesting, trade and enterprises, and others). These are not prioritized, nor are the bullet points under each heading. Note that medicinal plants are a subset of non-timber forest products.

*3.1 Related to medicinal plant resource management*

- Conservation of traditional ethnobotanical knowledge
- Conservation through cultivation through people's participation
- Private sector involvement in cultivation
- Develop simple, low-cost biotechnological tools and interventions to conserve our wild resources (such as DNA barcoding and production of synthetic alternatives to wild harvested products)
- The issues of all stakeholders, ranging from harvesters of wild resources to consumers, should be addressed within a common policy framework to manage wild natural resources
- To conduct national-level non-timber forest product study and make detailed management plans
- Develop nurseries for medicinal plants and high-value wild species by government institutions and distribute for plantations in community forests, fallow lands, and buffer zones
- Effective implementation of conservation and sustainable use of non-timber forest products
- In community forestry, there is a need to make simple and understandable Operational Plans by user groups and incorporate scientific forest management

*3.2 Related to medicinal plant harvesting*

- Sustainable wild harvests
- Sustainable harvesting and marketing arrangements with friendly policy development
- Livelihood improvement through plant resources
- The government should develop site-specific directives for specific non-timber forest product collection (not just common at the national level)
- Distribution of wild products must be directed to lower the Gini coefficient, and the Lorenz curve must be shifted towards the nearness of the line of perfect distribution
- Research in the wild harvests, medicinal plants with potential of harvest should be increased

*3.3. Related to medicinal plant trade and enterprises*

- Abrupt ban or regulation order on certain species and products from wild harvest does not benefit the trade and/or livelihoods of the collectors; and, hence, should be amended
- Support and develop a market network for high-value species
- Small-scale rural enterprise development in rural areas
- Develop infrastructure for processing and value addition to non-timber forest products to prevent the export of plant resources in unprocessed forms
- The government should take immediate action for non-timber forest product market access as entailed in The Herbs and Non-Timber Forest Products Development Policy, 2061 (2004)
- Enterprise development is needed
- Systematic harvest, taxation, legalizing the procedures to reduce illegal trade and strict implementation of the rules formulated are essential
- Experts from every sector, such as a taxonomist for proper identification of plants traded across borders

*3.4 Other recommendations (e.g. demand-side interventions)*

- Create awareness among students (younger generation), villagers, and farmers on the conservation and sustainable use of wild plants
- Scientific inputs on validation of biological activities
- Participation of relevant organizations should be compulsory while making forest policy and revenue decisions
- Institutional and incentive mechanism development for private sector engagement in forest and forest-based industry development in line with the Industrial Act 2073 (2017) and the Forest Act 2076 (2019)
- Action research on ethnobotany in rural areas
- Each green sector office should undertake evident base research activities
- Policy preparation, field-level assessment and implementation should address the private sector
- The flow of money for the development and promotion of non-timber forest products should be analyzed
- Interlink the scientific research into the development program to collaborate hand in hand
- Mainstreaming of non-timber forest products in policy is needed
- Collaboration between students, researchers, funding agencies, and policy makers is vital for sound results and implementation in local sectors, sharing of ideas
- Timely sharing of knowledge between researchers of different nations and policy makers is vital
- Regional collaboration should be enhanced
- Enabling environment for proper forest resource inventory
- Trial plots should be developed for valuable forest products

# Appendix S4 List of consulted documents for the development of the roadmap

All documents related to the roadmap development are listed below. As per the description of the methods in the main text, the list is derived from Smith-Hall et al. (2020: Trade and conservation of medicinal and aromatic plants – an annotated bibliography for Nepal. Sopan Press, Kathmandu) and the additional Web of Science search.

Acharya, T.P. 2000. Conservation of non-timber forest products (NTFPs) in Humla, Nepal: a case study of Rudikot (Margor) region for biodiversity conservation. In: Proceedings of the Third Regional Workshop on Community Based NTFP Management, Kathmandu. South and East Asian Countries NTFP Network (SEANN), pp. 264-271.

Acharya, S.P. 2005. Developing Medicinal and Aromatic Plant based Livelihood Options for Traditional Gathering Communities: A case study of Western part of Nepal - producing key recommendations for policy interventions and collaborative and comparative study across Maharashtra and Tamil Nadu India. Ministry of Local Development, Government of Nepal, Western Uplands Poverty Alleviation Project, Nepalgunj.

Acharya, K.R. 2013. What Makes The Value Chain Development Intervention Success? Particular Emphasis on Medicinal and Aromatic Plant Sub-Sector in Mid-Western, Nepal. In: Balla, M.K., Rayamanjhi, S. and Singh, A. (Eds.), Proceedings of International Conference on Forests, People and Climate: Changing Paradigms, 28-30 August, 2013, pp. 297-312.

Acharya, G.R., Koirala, P.N., Neupane, L. and Devkota, S.C. 2009. Livelihood option from minor forest produce: context of non timber forest product and poverty reduction in mid hills of Nepal. Journal of Wetlands Ecology 2(1&2): 57-66.

Adhikary, P.M. 1993. Medicinal and aromatic plants in Nepal. In: Chomchalow, N. and Henle, H.V. (Eds.), Medicinal and aromatic plants in Asia. FAO/RAPA Publication 1993/19, pp. 138-144.

Adhikari, M.K. 2000. Morels and their production in natural environment of Jumla district, Nepal. Banko Janakari 10(1): 11-14.

Adhikari, I.P. and Das, B. 1999. Study of five commercially threatened medicinal herbs. In: Proc. III National Conference on Science and Technology, RONAST, Kathmandu, pp. 1530-1534.

Adhikari, S.R., Manandhar, N. P. and Vaidya, L. K. 1986. Aromatic plants of Nepal part V. A note on the availability, trade practices and quality assessment of the fruits of Zanthoxylum alatum of western Nepal. J. Nepal Pharm. Assoc. 13(1-2): 21-28.

Adhikary, P. 1996. Study on non-timber forest products, their management and impacts on socio-economy with emphasis on medicinal herbs and nigalo (Arundinaria sp.). Asia Network for Small-scale Agricultural Biotechnologies, Kathmandu, 68 pp.

AEC, 2006. A Report on Compilation and Prioritization of Ten Important NTFPs of Nepal for Commercial Promotion through Private Sector Investment. Federation of Nepalese Chambers of Commerce and Industry, Agro Enterprise Center (AEC/FNCCI), Kathmandu.

Amatya, G. 1995. A preliminary study of medicinal plants in the Bhotekhola and Tamku regions of the Sankhuwasabha District for commercial scale cultivation. The Mountain Institute and Department of National Parks and Wildlife Conservation. Kathmandu, 53pp.

Amatya, K.R. 2003. MAPs trade and promotion. Plant Resources 22: 104-117. Department of Plant Resources, Kathmandu.

Amatya, G. 2006. Trade and Socio-Economic Attribution of Cordyceps sinensis (Berk) Saccardo in Darchula District, Nepal. In: Jha, P.K., Karmacharya, S.B., Chhetri, M.K., Thapa, C.B. and Shrestha, B.B. (Eds.), Medicinal Plants in Nepal: an Anthology of Contemporary Research. Ecological Society (ECOS), Kathmandu, pp. 194-203.

Amatya, K.R. and Amatya, G. 1995. Medicinal plants in Gorkha District. In: Yadav, S.K. and Stoian, D. (Eds.), Proceedings of the seminar on medicinal and aromatic plants in Gorkha District: how to promote their utilisation and marketing. District Forest Office, Gorkha bazaar, pp. 17-24.

Amatya, S.M. and Stoian, D. (Eds.) 1995. Proceedings of the seminar on medicinal and aromatic plants in Gorkha District: how to promote their utilisation and marketing. German Development Service, District Forest Office, Gorkha bazaar, 28 pp.

ANSAB, 1997a. Environment and forest enterprise activity: forest products market/enterprise options study. Asia Network for Small-scale Agricultural Bio-resources, Kathmandu, 107 pp.

ANSAB. 1997b. Non-timber forest products: final technical report. Asia Network for Small-scale Agriculture Bioresources, Kathmandu, 18 pp.

ANSAB, 2002. Community forest wise raise the royalty of yarsa-Gumba. Lahara 1(3).

ANSAB, 2003a. Commercially Important Non Timber Forest Products (NTFPs) of Nepal. Asia Network for Sustainable Agriculture and Bioresources and Netherlands Development Organization (SNV/Nepal), Kathmandu.

ANSAB, 2003b. Non-Timber Forest Products (NTFPs) in Newspaper. Asia Network for Sustainable Agriculture and Bioresources, Kathmandu.

ANSAB and EWW. 1999. Enterprise development for natural products: a focus on non-timber forest products. A draft manual. Asia Network for Small-Scale Bioresources and Enterprises Works Worldwide, Kathmandu, 41 pp.

Aryal, M. 1993. Diverted wealth: the trade in Himalayan herbs. Himal 1: 9–18.

Aryal, K.P., Berg, Å. and Ogle, B. 2009. Uncultivated plants and livelihood support – a case study from the Chepang people of Nepal. Ethnobotany Research & Applications 7: 409–422.

Aryal, A. and Pelz, D.R. 2008. Non-Timber Forest Products Resource Assessment & Plan for Effective Management (A Case Study from Matribhumi Community Forestry at Dovan VDC of Palpa District, Nepal). Faculty of Forest and Environment Science, Albert Ludwig University of Freiburg, Freiburg.

Aumeeruddy-Thomas, Y. and Karki, M. 2005. Himalayan medicinal and aromatic plants: perspectives for balancing use and conservation. In: Y. Aumeeruddy-Thomas, M. Karki, K. Gurung and D. Parajuli (Eds.) Himalayan Medicinal and Aromatic Plants, Balancing Use and Conservation. Proceedings of the Regional Workshop on Wise Practices and Experiential Learning in Conservation and Management of Himalayan Medicinal Plants, December 15–20, 2002. His Majesty’s Government of Nepal, Ministry of Forests and Soil Conservation, Kathmandu, pp. 2-37.

Awasthi, H.P. 2009. NTFPs Marketing System in the Far-west Hills of Nepal with Reference to NTFPs Marketing System of Baitadi District in Value Chain Perspective. MSc thesis, Pokhara University, Pokhara.

Banjade, M.R. and Paudel, N.S. 2008. Economic Potential of Non-timber Forest Products in Nepal: Myth or Reality? Journal of Forest and Livelihood 7(1): 36-48.

Baral, K. 2013. Collection and utilization of Non-Timber forest products (NTFPs) from the high hill of Nepal. MSc Dissertation, Institute of Forestry, Tribhuvan University, Pokhara, Nepal.

Baral, S.R. and Kurmi, P.P. 2006. A Compendium of Medicinal Plants in Nepal. Rachana Sharma, Maujubahal, Kathmandu.

Bashyal, B.P., Bhattarai, N.K. and Pradhan, J. 1994. Role of research and development in commercial utilization of non-timber forest products: medicinal and aromatic plants. In J. Pradhan and P. Maharjan (Eds.), Proceedings of the National Seminar on NTFPs: Medicinal and Aromatic Plants. September 11-12. Ministry of Forest and Soil Conservation and Herbs Production and Processing Company Limited, Kathmandu, pp. 27-34.

Bashyal, R., Paudel, K., Hinsley, A. and Phelps, J. 2023. Making sense of domestic wildlife and CITES legislation: The example of Nepal's orchids. Biological Conservation 280: 109951.

Bashyal, R. and Roberts, D.L. 2023. Assessing the identification uncertainty in plant products traded as traditional Asian medicines. Journal for Nature Conservation 73: 126410.

BDS‐MaPS. 2007.  Project Completion Report. BDS–MaPS Bakhundole, Lalitpur, USAID-Nepal.

Bhandari, N.B. 1997. The possibility and potentiality of cultivation for high mountainous medicinal plants – a case study from Humla District. Institute of Forestry, Pokhara, 52pp.

Bhatta, B.K. and Das, C.L. 1996. The status of non-timber forest products (NTFPs) in Daman area. Final report. Asia Network for Small Scale Bioresources. Kathmandu, 41pp.

Bhatta, D.B. and Rawal, R.B. 2001. Policy and Regulatory Environment in relation to Jaributi Production in Nepal. In: Proceedings of the National Workshop on Non-Timber Forest Products: Jaributi. February 22, 2001. Kathmandu. Pp 10-16.

Bhattarai, N.K. 1995. Prospects of community based NTFP enterprise development for promoting biodiversity conservation and economic development in Gorkha district, Nepal. In S. M. Amatya and D. Stoian (Eds.), Proceedings of the Seminar on Medicinal and Aromatic Plants in Gorkha district: How to promote their utilization and marketing. District Forest Office, Gorkha/German Development Service, Kathmandu, pp. 14-18.

Bhattarai, N.K. 1997. Medicinal and aromatic plants of Nepal. In: M. Karki, A.N. Rao, R. Rao and J.T. Williams (Eds.) The Role of Bamboo, Rattan and Medicinal Plants in Mountain Development. International Network for Bamboo and Rattan, IDRC, New Delhi, pp. 162-173.

Bhattarai, D.R. 2000. Utilization, Industrialization and Commercialization of Himalayan Medicinal Resouces. Proceedings of Nepal-Japan Symposium on Conservation and Utilization of Himalayan Medicinal Resources, Nov 6-11, 2000, Department of Plant Resources and Society for the Conservation and Development of Medicinal Plant Resources, Kathmandu, pp. 355-358.

Bhattarai, K.R. and Acharya, N. 1998. Swertia species (chiraito) in commerce in Nepal. Plant Research 1(1): 48-55.

Bhattarai, S. and Balla, B.K. 2007. Status, Distribution and Market Analysis of Nagbeli (Lycopodium clavatum) in Banglung District. In: Abstracts: National Seminar on Sustainable Use on Biological Resources with the special theme: Medicinal and Aromatic Plants. April 22-23, 2007, Pokhara.

Bhattarai, N.K. and Croucher, J. 1996. The viability of local commercialization of non-timber forest products as a strategy for promoting biodiversity conservation. In: P.K. Jha, G.P.S. Ghimire, S.B. Karmacharya, S.R. Baral, and P. Lacoul (Eds.) Environment and Biodiversity in the Context of South Asia. Ecological Society, Kathmandu, pp. 346-353.

Bhattarai, K.R. and Ghimire, M.D. 2006. Commercially important medicinal and aromatic plants of Nepal and their distribution pattern and conservation measure along the elevation gradient of the Himalayas. Banko Janakari 16(1): 3-13.

Bhattarai, N.K. and Olsen, C.S. 2000. Towards a generic framework for investigating national importance of medicinal plant trade. In: Proceedings of the Third Regional Workshop on Community Based NTFP Management, Kathmandu. South and East Asian Countries NTFP Network (SEANN), pp. 336-346.

Bhattarai, N.K. and Shukla, R.N. 2000. Potentials of NTFP processing and marketing assistance in the resource conservation and economic development in the Mid-western Development Region of Nepal. In: Proceedings of the Third Regional Workshop on Community Based NTFP Management, Kathmandu. South and East Asian Countries NTFP Network (SEANN), pp. 206-215.

Bista, S. and Webb, E.L. 2006. Collection and marketing of non-timber forest products in the far western hills of Nepal. Environmental Conservation 33(3): 244-255.

Burbage, M.B. 1981. Report on a visit to Nepal: the medicinal plant trade in the Khardep area: a study of the development potential. Tropical Products Institute, Overseas Development Administration, London, UK.

Cameron, M.M. 1996. Biodiversity and Medicinal Plants in Nepal: Involving Untouchables in Conservation and Management. Human Organization 55(1): 84-92.

Caporale, F., Mateo-Martín, J., Usman, F. and Smith-Hall, C. 2020. Plant-based sustainable development – the expansion and anatomy of the medicinal plant secondary processing sector in Nepal. Sustainability 12(14): 5575.

CBED. 1998. Training on management, marketing and cultivation of non-timber forest products in Jumla. Community Based Economic Development Project (CBED). 28 pp.

CECI. 1999. Subsector Analysis of High Altitude NTFPs in the Karnali Zone. Vol. 1: Main Report. Canadian Centre for International studies and Cooperation (CECI), Kathmandu, 47 pp.

CECI. 2002. Information dissemination on management and marketing of endangered and high altitude medicinal and aromatic plants in Jumla district of Nepal. Canadian Centre for International studies and Cooperation (CECI), Kathmandu.

CEDA. 2004a. A Synthesis Report on the current status of Non-Timber Forest Products in the Terai region of Nepal. ITTO Project No. PPD 6/99 Rev. 3 (M. F.1). Centre for Economic Development and Administration, Kathmandu.

CEDA. 2004b. Completion Report on Preparation of Project Proposal for the Promotion of Non-Timber Forest Products in the Terai Region of Nepal. ITTO Project No. PPD 6/99 Rev. 3 (M. F.1). Centre for Economic Development and Administration, Kathmandu.

Chapagain, D.R. 1983. Rudrakshaya cultivation, production and marketing in Bhojpur district. MA Thesis, 157pp + annexes.

Chapagain, N. 2000. Rudraksha (Elaeocarpus sphericus) an Important Medicinal and Religious Plant. Poster Paper. Proceedings of Nepal-Japan Joint Symposium 2000. Pp 313-316.

Chapagain, A. 2020. Formal versus informal practices: Trade of medicinal and aromatic plants via Trans-Himalayan Silk Road. In Ngo, T.W and Hung, E.P.W. (Eds.) Shadow exchanges along the new silk roads, Amsterdam University Press, Amsterdam, pp. 145-161.

Chapagain, D.J., Meilby, H. and Ghimire, S.K. 2019. Plant density and life history traits of Aconitum spicatum in North-central Nepal: effects of elevation and anthropogenic disturbances. PeerJ 7: e7574.

Chapagain, S.P., Rai, J.K., Pathak, A., Swanson, T.V., Cruz, A., Mueller, K., Diemer, A.H. and Dahal, K. 2014. Value Chain Analysis of Forest Products in Koshi Hill Districts of Nepal: Challenges and Opportunities for Economic Growth. ForestAction Nepal and Rural Reconstruction Nepal (RRN), Kathmandu.

Charmakar, S. Kunwar, R.M. Sharma, H.P., Rimal, B., Baral, S., Joshi, N., Gauli, K., Acharya, R.P. and Oli, B.N. 2021. Production, distribution, use and trade of Valeriana jatamansi Jones in Nepal. Global Ecology and Conservation 30: e01792.

Chaudhary, R.P. 1998. Biodiversity in Nepal – Status and Conservation. Know Nepal Series No. 17. S. Devi, Saharanpur (U.P.), India. 324 pp.

Chaudhary, R.P., Shrestha, K.K., Jha, P.K. and Bhatta, K.P. 2010. Kailash Sacred Landscape Conservation Initiative Feasibility Assessment Report – Nepal. Central Department of Botany, Tribhuvan University, Kirtipur, Nepal.

Chhetri, D.B. 1999. Diversity of Medicinal and Aromatic Plants in Manang, Central Nepal with Emphasis on Ecology and Essential Oil Variation of Jatamansi (Nardostachys grandiflora DC.). MSc Dissertation, Central Department of Botany, Tribhuvan University, Kirtipur, Kathmandu, Nepal.

Chhetri, R.B. 2006a. Report on conservation and cultivation expansion of Kaulo exported from Mid-Western Development Region under Nepal Trade Integrate Strategy (Forest Program). Department of Plant Resources, Ministry of Forest and Soil Conservation, Government of Nepal, Kathmandu.

Chhetri, R.B. 2006b. From protection to poverty reduction: a review of forestry policies and practices in Nepal. [Journal of Forest and Livelihood](https://www.nepjol.info/index.php/JFL/index) 5: 66-77.

Chhetri, H.B. and Gupta, V.N.P. 2006. NTFP potential of Upper Mustang - a Trans-Himalayan region in Western Nepal. Scientific World 4(4): 38–43.

Chhetri, H.B. and Gupta, V.N.P. 2007. A survey of non-timber forest products (NTFPs) in upper Mustang. Scientific World 5(5): 89-94.

Chhetri, R. and Lodhiyal, L.S. 2006. Collection of Cordyceps sinensis (Berk.) Sacc. (Yarsagumba) and its Implications to Rural Livelihood and Biodiversity Conservation: A case of Darchula District, Nepal. In: Jha, P.K., Karmacharya, S.B., Chhetri, M.K., Thapa, C.B. & Shrestha, B.B. (Eds.) 2006. Medicinal Plants in Nepal: an Anthology of Contemporary Research. Ecological Society (ECOS), Nepal. Pp 214-222.

Chhetri, R.B., Kharel. S. and Khanal, P. 2003. Non-Timber Forest Products Management for Income Generation and Biodiversity Conservation: A case Study from Kavre District, Nepal. In: Neupane F.P., Bajracharya, K.M. and Bhuju, D.R. (Eds.) 2003. Proceedings of International Seminar on Mountains, Kathmandu, March 6-8, 2002, Royal Nepal Academy of Science and Technology, Kathmandu, pp. 473-477.

Childs, G. and Choedup, N. 2014. Indigenous Management Strategies and Socioeconomic Impacts of Yartsa Gunbu (Ophiocordyceps sinensis) Harvesting in Nubri and Tsum, Nepal. Himalaya 34(1): 8-22.

Choudhary, D., Pandit, B.H., Kala, S.P., Todaria, N.P., Dasgupta, S. and Kollmair, M. 2014. Upgrading bay leaf farmers in value chains—strategies for improving livelihoods and poverty reduction from Udayapur district of Nepal. Society & Natural Resources 27: 1057–1073.

Choudhary, D., Pandit, B.H., Khinal, G. and Kollmair, M. 2011. Pro-poor value chain development for high value products in mountain regions: Indian Bay Leaf. Kathmandu: ICIMOD.

Choudhary, D., Kala, S., Todaria, N., Dasgupta, S. and Kollmair, M. 2013. Marketing of Bay Leaf in Nepal and Northern India: Lessons for Improving Terms of Participation of Small Farmers in Markets. Small-scale Forestry 12(2): 289-305.

Christensen, M., Bhattarai, S., Devkota, S. and Larsen, H.O. 2008. Collection and Use of Wild Edible Fungi in Nepal. Economic Botany 62(1): 12-23.

Christensen, M. and Larsen, H.O. 2005. How can collection of wild edible fungi contribute to livelihoods in rural areas of Nepal? Journal of Forest and Livelihood 4(2): 50-55.

Cunningham, A.B., Brinckmann, J.A., Bi, Y.F., Pei, S.J., Schippmann, U. and Luo, P. 2018. Paris in the spring: A review of the trade, conservation and opportunities in the shift from wild harvest to cultivation of Paris polyphylla (Trilliaceae). Journal of Ethnopharmacology 222: 208-216.

Cunningham, A.B., Brinckmann, J.A., Pei, S.J., Luo, P., Schippmann, U., Long, X. and Bi, Y.F. 2018. High altitude species, high profits: Can the trade in wild harvested Fritillaria cirrhosa (Liliaceae) be sustained? Journal of Ethnopharmacology 223: 142-151.

Cunningham, A.B., Brinckmann, J.A., Schippmann, U. and Pyakurel, D. 2019. Production from both wild harvest and cultivation: The cross-border Swertia chirayita (Gentianaceae) trade. Journal of Ethnopharmacology 225: 42-52.

Dangal, S. 2000. Non-timber forest products: an alternative source of rural income. Journal of Forest and Livelihood 2(1): 56-58.

Dangi, R.B. 2008. Impact of NTFP Harvesting in Forest Conservation. The Initiation 2(1): 165 – 171.

Daniggelis, E.K. 1996. Swertia chirayita can supplement rural income in Nepal. Banko Janakari 6(2): 68-73.

Das, B. 2004. Cinnamomum species: Potentialities for Better Income Generation through Improved Management Practices. In: N.K. Bhattarai and M. Karki (Eds.) Local Experience based National Strategy for Organic Production and Management of MAPs/NTFPs in Nepal. IDRC/MAPPA, CCO and Government of Nepal. Pp 224-227.

DeCoursey, M.A. 1993. Research into the collection and trade of jaributi from the middle hills of Nepal: The Annapurna Conservation Area. In: Edwards, D.M. and Bowen, M. R. (Eds.), Focus on jaributi. Forest Research and Survey Centre. Occasional Paper 2/93, Kathmandu Nepal, p. 9.

DeCoursey, M.A. 1994. Local-level marketing of non-timber forest products from Nepal’s Middle Hills: Three case studies. Yale School of Forestry and Environmental Studies, New Haven. 22p.

Devkota, S. 2006a. Yarsagumba [Cordyceps sinensis (Berk) Sacc]; traditional utilization in Dolpa District, Western Nepal. Our Nature 5: 48-52.

Devkota, S. 2006b. Approach towards the Harvesting of Cordyceps sinensis (Berk.) Sacc. in Pastures of Dolpa, Nepal. In: Jha, P.K., Karmacharya, S.B., Chetri, M.K., Thapa, C.B. and Shrestha, B.B. (Eds.), Medicinal Plants in Nepal: An Anthology of Contemporary Research, (Ecological Society (ECOS), Kathmandu. Pp. 90-96.

Devkota, S., Chaudhary, R.P., Werth, S. and Scheidegger, C. 2017. Trade and legislation: consequences for the conservation of lichens in the Nepal Himalaya. Biodiversity and Conservation 26(10): 2491-2505.

Devkota, S. and Shrestha, A. 2006. A Study on Ethno-ecology, Regeneration Pattern, Collection Techniques and Trade of Yarsagumba (Cordyceps sinensis): Research in Kangchenjunga Conservation Area and Dolpa Region of Nepal for Policy Recommendations [Report]. SAFE Concern and WWF Nepal Program, Kathmandu.

Dhital, P.P. 2006. Non-Timber Forest Products: The Diversity and Contribution to the Livelihood of People. MSc Dissertation, Institute of Forestry, Tribhuvan University, Pokhara.

Dhungana, S.P. and Bhattarai, R.C. 2008. Exploring Economic and Market Dimensions of Forestry Sector in Nepal. Journal of Forest and Livelihood 7(1): 58-69.

Dhungana, H.P. and Dahal, S.P. 2004. Strengthening Local Capacity for Non-Timber Forest Products Management and Marketing: The Need for Policy Reforms in Community Forestry in Nepal. In: Proceedings of the Fourth National Workshop on Community Forestry. Community Forestry Division, Department of Forest. 4-6 August, 2004. Kathmandu. Pp 142-149.

Dobremez, J.F. 1982. Exploitation and prospects of medicinal plants in eastern Nepal. In Mountain Environment and Development, A collection of papers published on the occasion of the 20th anniversary of the Swiss Association for Technical Assistance in Nepal (SATA), Sahayogi Press, Kathmandu, pp. 97-107.

DPR. 2006. Prioritized Medicinal Plants for Economic Development in Nepal (Nepal ko Aarthik Bikaska lagi Prathamikata Prapta Jadibutiharu). Department of Plant Resources, Ministry of Forest and Soil Conservation, Government of Nepal, Kathmandu.

Dürbeck, K. and Wildner, A. 1993a.Trade promotion of extracts from medicinal and aromatic plants and of phytopharmaceuticals. In Business Symposium on Herbs extracts/Essential oils, Nepal-German Chamber of Commerce and Industry, Kathmandu, no paging (2pp).

Dürbeck, K. and Wildner, A., 1993b. Trade promotion of essential oils from developing countries to the Federal Republic of Germany.In Business Symposium on Herbs extracts/Essential oils, Nepal-German Chamber of Commerce and Industry, Kathmandu, no paging (3pp).

Edwards, D.M. 1993a. Research into the collection and trade of jaributi from the middle hills of Nepal: The Koshi hills. In: Edwards, D.M. and Bowen, M.R. (Eds.), Focus on jaributi. Forest Research and Survey Centre. Occasional Paper 2/93, Kathmandu, p. 9.

Edwards, D.M. 1993b. The marketing of non-timber forest products from the Himalayas: the trade between east Nepal and India. Rural Development Forestry Network Paper 15b. Overseas Development Institute, London. 24 pp.

Edwards, D.M. 1994. Non-timber forest products and community forestry. Nepal-UK Community Forestry Project, Kathmandu, 36 pp.

Edwards, D.M. 1996a. Non-timber forest products from Nepal: aspects of the trade in medicinal and aromatic plants. FORESC Monograph no. 1/96, Forest Research and Survey Centre, Ministry of Forest and Soil Conservation, Kathmandu. 134 pp.

Edwards, D.M. 1996b. The trade in non-timber forest products from Nepal. Mountain Research and Development 16(4): 383-394.

Edwards, D.M. and Bowen, M.R., 1993. Focus on jaributi. FRSC Occasional Paper 2/93, Forest Research and Survey Centre, Ministry of Forest and Soil Conservation, Kathmandu.26 pp.

Fold, N., Pyakurel, D., Pouliot, M. and Smith-Hall, C. 2023. Global production networks and medicinal plants: upstream actors in Nepal. Geographical Journal, 189, 455–468.

Gahire, S. 2003. Ecology, Distribution and Trade of Kutki [Neopicrorhiza scrophulariiflora (Pennell) Hong] in Manang District, Nepal (Case study of Chame and TachiBagarchhap VDCs). MSc Dissertation, Central Department of Botany, Tribhuvan University, Kirtipur, Kathmandu.

Gaire, D. 2005. An Assessment of Non-Timber Forest Products Marketing in Community Forests: A case study From Lamjung District. BSc Thesis. Tribhuvan University, Institute of Forestry, Pokhara.

Gaire, D., Jiang, L., Adhikari, B., Bhattarai, S. and Panthi, S. 2022. Predicting the potential distribution, trade, and conservation of Rauvolfia serpentina in Nepal. Applied Ecology and Environmental Research 20(6): 4999-5022.

Gauli, K. 2011. Commercialization of Non-timber Forest Products: Contribution to poverty reduction in Dolakha district, Nepal. Doctoral Thesis. Centre for Development Research & Department of Sustainable Agricultural Systems, University of Natural Resources and Life Sciences, Vienna.

Gauli, K. and Hauser, M. 2009. Pro-poor commercial management of non-timber forest products in Nepal's community forest user groups: factors for success. Mountain Research and Development 29(4): 298-307.

Gauli, K. and Hauser, M. 2011. Commercial management of non-timber forest products in Nepal’s community forest users groups: who benefits? International Forestry Review 13(1): 35-45.

Gautam, K.H., and Devoe, N.N. 2002. Conflicts between policy and local people in valuing non-timber forest products: perspectives from Nepal. Journal of Forests and Livelihoods 2(1): 43-52.

Ghimire, O. 1995. Medicinal plants in Gorkha district: Opportunities and limitations for their promotion. In: Yadav, S.K. and Stoian, D. (Eds.), Proceedings of the seminar on medicinal and aromatic plants in Gorkha district: How to promote their utilization and marketing. District Forest Office, Gorkha. pp. 25-27.

Ghimire, S.K. 2006. Sustainable Harvesting and Management of Medicinal Plants in the Nepal Himalaya: Current Issues, Knowledge Gaps and Research Priorities. In: Jha, P.K., Karmacharya, S.B., Chhetri, M.K., Thapa, C.B. and Shrestha, B.B. (Eds.), Medicinal Plants in Nepal: an Anthology of Contemporary Research. Ecological Society (ECOS), Nepal. Pp 25-42.

Ghimire, S.K. 2009. Management Plan for Sustainable Collection of Neopicrorhiza scrophulariiflora: Tiptala-Bhanjyang and Kumbhakarna Conservation Community Forests, Kangchenjunga Conservation Area (KCA), Taplejung, Nepal. Report submitted to WWF Nepal, Kathmandu.

Ghimire, S.K. and Aumeeruddy-Thomas, Y. 2005. Approach to in situ conservation of threatened Himalayan medicinal plants: a case study from Shey-Phoksundo National Park, Dolpo. In: Aumeeruddy-Thomas, Y., Karki, M., Gurung, K. and Parajuli, D. (Eds.), Himalayan Medicinal and Aromatic Plants, Balancing Use and Conservation, Ministry of Forests and Soil Conservation, Kathmandu, pp. 209-234.

Ghimire, S.K., Awasthi, B., Rana S., Rana, H. and Bhattarai, R. 2015. Status of Exportable, Rare and Endangered Medicinal and Aromatic Plants (MAPs) of Nepal. Ministry of Forest and Soil Conservation (MoFSC), Department of Plant Resources (DPR), Thapathali, Kathmandu.

Ghimire, S.K., Gimenez, O., Pradel, R., McKey, D. and Aumeeruddy-Thomas, Y. 2008b. Demographic variation and population viability in a threatened medicinal and aromatic herb (Nardostachys grandiflora): effects of harvesting in two contrasting habitats. Journal of Applied Ecology 45: 41-51.

Ghimire, S.K., Lama, Y.C., Tripathi G.R., Schmitt S. and Aumeeruddy-Thomas, Y. 2001. Conservation of Plant Resources, Community Development and Training in Applied Ethnobotany at Shey-Phoksundo National Park and its Bufferzone, Dolpa. Third year. WWF Nepal Program Report Series No. 41, WWF Nepal Program, Kathmandu, Nepal.

Ghimire, S.K., McKey D. and Aumeeruddy-Thomas, Y. 2005. Conservation of Himalayan medicinal plants: harvesting patterns and ecology of two threatened species, Nardostachys grandiflora DC. and Neopicrorhiza scrophulariiflora (Pennell) D.Y. Dong. Biological Conservation 124: 463-475.

Ghimire S.K., McKey D. and Aumeeruddy-Thomas, Y. 2006. Himalayan medicinal plant diversity in an ecologically complex high-altitude anthropogenic landscape, Dolpo, Nepal. Environmental Conservation 33: 128-140.

Ghimire, S.K. and Nepal, B.K. 2007. Developing a Community-Based Monitoring System and Sustainable Harvesting Guidelines for Non-Timber Forest Products (NTFP) in Kangchenjunga Conservation Area (KCA), East Nepal. WWF Nepal program, Kathmandu.

Ghimire, S.K. and Pyakurel D. 2009. Management Plan for Sustainable Wild Collection of Swertia chirayita in Syaubari Buffer Zone Community Forest, Langtang National Park Buffer Zone Area, Rasuwa, Nepal. Report submitted to WWF Nepal, Kathmandu.

Ghimire, S.K, Pyakurel, D., Nepal, B.K., Sapkota, I.B., Parajuli, R.R. and Oli, V.R. 2008. A Manual of NTFPs of Nepal Himalaya. WWF Nepal program, Kathmandu, 206 pp.

Ghimire, S.K., Sah, J.P., Shrestha, K.K. and Bajracharya, D. 1999. Ecological Study of Some High Altitude Medicinal and Aromatic Plants in the Gyasumdo Valley, Manang, Nepal. Ecoprint 6(1):17-25.

Ghimire, S.K., Sapkota, I.B., Oli, B.R. and Parajuli-Rai, R. 2008. Non-Timber Forest Products of Nepal Himalaya: Database of Some Important Species Found in the Mountain Protected Areas and Surrounding Regions. WWF Nepal, Kathmandu.

GoN. 1970. Forest Products (Sales and Distribution) Rules. Government of Nepal.

GoN. 1972. Plant Protection Act. Government of Nepal.

GoN. 1973. National Park and Wildlife Conservation Act. Government of Nepal.

GoN. 1976. National Forest Policy. Government of Nepal.

GoN. 1985. Seventh Five Year Plan. Government of Nepal.

GoN. 1988. National Conservation Strategy. Government of Nepal.

GoN. 1992. Trade and Transit Agreement. Government of Nepal.

GoN. 1992 – 1997. Eighth Five Year Plan. Government of Nepal.

GoN. 1993. Environmental Impact Assessment Guidelines for Forestry Sector. Government of Nepal.

GoN. 1993. Forest Act and 1995. Forest Regulation. Government of Nepal.

GoN. 1993 and 1998. Nepal Environmental Policy and Action Plan (NEPAP). Government of Nepal.

GoN. 1996. Environment Protection Act. Government of Nepal.

GoN. 1997 – 2002. Ninth Five Year Plan. Government of Nepal.

GoN. 1999a. EIA Guidelines for the Forestry Sector. Government of Nepal, National Planning Commission, Ministry of Forest and Soil Conservation and IUCN.

GoN. 1999b. Local Self-Governance Act (LSGA). Government of Nepal.

GoN. 2000a. Forestry Sector Policy. Government of Nepal.

GoN. 2000b. Local Self Governance Regulation. Government of Nepal.

GoN. 2001. Genetic Resource Bill Draft. Government of Nepal.

GoN. 2002a. Nepal Biodiversity Strategy. Ministry of Forest and Soil Conservation & Global Environmental Facility and UNDP, Kathmandu.

GoN. 2002b. Nepal Biodiversity Strategy. Government of Nepal.

GoN. 2002 – 2007. 10th Five‐year Plan. Government of Nepal.

GoN. 2004a. Herbs and NTFPs policy. Government of Nepal.

GoN. 2004b. National Action Programme on Land Degradation and Desertification. Government of Nepal, Ministry of Population and Environment.

GoN. 2005. Guideline for NTFP Based Enterprise. Government of Nepal.

GoN. 2006. Nepal Biodiversity Strategy Implementation. Government of Nepal.

GoN. 2007. Plant Protection Act. Government of Nepal, Nepal Law Commission.

GoN. 2007-2010. 11^th^ Three Year Interim Plan. Government of Nepal, National Planning Commission.

GoN. 2009. Community Forest Development Guidelines. Government of Nepal, Ministry of Forest and Soil Conservation.

GoN. 2010a. Industrial Policy 2067. Government of Nepal.

GoN. 2010b. Plant Protection Rules. Government of Nepal, Nepal Law Commission.

GoN. Ministry of Agricultural Development, 2011. A Report on Value Chain Analysis of Timur. High Value Agriculture Project in Hill and Mountain Areas (HVAP). Project Management Unit, Birendranagar, Surkhet.

GoN. Ministry of Forests and Soil Conservation, 2013a. Quality Standards, Good Agricultural and Collection Practice (GACP) of Rauvolfia serpentina (L.) Benth. Ex. Kurz. Department of Plant Resources, Thapathali, Kathmandu.

GoN. Ministry of Forests and Soil Conservation. 2013b. Value Chain designing of kurilo of Panchase Protected Forest Area. Department of Forest.

GoN. 2014. Forest Sector Strategy. Draft.

GoN. 2014 – (2020). Nepal National Biodiversity Strategy and Action Plan (NBSAP). Government of Nepal, Ministry of Forests and Soil Conservation, Kathmandu.

GoN. 2015a. Nepal REDD+ Strategy Part 1: Operational Summary. Government of Nepal, Ministry of Forest and Soil Conservation, REDD Implementation Centre, Katmandu.

GoN. 2015b. Community Forest Products Sales Guidelines. Government of Nepal, Ministry of Forest and Soil Conservation, Kathmandu.

Gurung, K. 2010. Essential Oils Sector Study in Nepal: A Detailed Study of Anthopogon, Juniper and Wintergreen Essential Oils. A report submitted to GTZ INCLUDE, Narayani Complex, Lalitpur.

Gurung, K. 2013. Study on Quality Issues of Medicinal and Aromatic Plants (MAPs) Sector in Nepal. A report submitted to The Physikalisch-Technische Bundesanstalt (PTB), Braunschweig, Germany.

Gurung, K. and Pyakurel, D. 2010. Assessment and profile preparation of high valued non-timber forest products (NTFPs) of Jajarkot district (Report). Western Uplands Poverty Alleviation Project (WUPAP), Nepalgunj, Banke.

Hamilton, F.B. 1819. An Account of the Kingdom of Nepal: And of the Territories Annexed to this Dominion by the House of Gorkha. Archibald Constable and Company, Edinburgh.

He, J., Yang, B., Dong, M. and Wang, Y.S. 2018. Crossing the roof of the world: Trade in medicinal plants from Nepal to China. Journal of Ethnopharmacology 224: 100-110.

[Heinen, J.T.](http://www.tandfonline.com/author/Heinen%2C+Joel+T) and  [Shrestha-Acharya](http://www.tandfonline.com/author/Shrestha-Acharya%2C+Ranju), R. 2011. The Non-Timber Forest Products Sector in Nepal: Emerging Policy Issues in Plant Conservation and Utilization for Sustainable Development. [Journal of Sustainable Forestry](http://www.tandfonline.com/toc/wjsf20/current)30(6): 543-563.

Hertog, W.D. 1993. Non-timber Forest Products in Dolpa District. Project report. Karnali Local Development Program, SNV, Kathmandu.

Hertog, W.D. 1997. Access makes the difference? Harvest and trade of non-timber forest product on communal and private land. Department of Sociology/Department of Forestry Wageningen Agricultural University, Wageningen. MSc thesis, 144pp + annexes.

[Hertog, W.D.](http://www.forestrynepal.org/publications/biblio?f%5bauthor%5d=518) and [Wiersum, K.F.](http://www.forestrynepal.org/publications/biblio?f%5bauthor%5d=519) 2000. Timur (Zanthoxylum armatum) production in Nepal. Dynamics in nontimber forest resource management. Mountain Research and Development 20(2):136-145.

HMG. 1988. Forest-based industries development plan, part II: medicinal and aromatic plants and other minor forest products. Master Plan for the Forestry Sector Nepal, Ministry of Forest and Soil Conservation, Kathmandu, pp. 82-146.

HMGN/ADB/FINNIDA. 1988. Medicinal and aromatic plants and minor forest products development. Master Plan for the Forestry Sector Nepal, Forest-Based industries Development plan Vol. IV Part II. HMGN/ADB/FINNIDA. Master Plan Document, Kathmandu, pp. 87-90.

Humagain, K. and Shrestha, K.K. 2006. Medicinal plants in Rasuwa district, Central Nepal: Trade and Livelihood. Botanica Orientalis 6: 39-46.

ICIMOD. 1996. Biodiversity bibliography. International Centre for Integrated Mountain Development, Kathmandu, 226 pp.

ICIMOD. 2015. Promotion of the Rittha (Soap Nut) Value Chain in Nepal: Sustainable Livelihoods in the Kailash Sacred Landscape. International Centre for Integrated Mountain Development, Kathmandu.

INCLUDE. 2014. Value Chain Development, Lessons Learnt from the Medicinal and Aromatic Plants (MAPs) Sub-sectors, Nepal. Inclusive Development of the Economy Programme (INCLUDE), Narayani Complex, Pulchowk, Lalitpur.

International Service Providers. 2011. Identification and Market Survey of Potentially Used Medicinal and Aromatic Plants (MAPs) in Eastern Nepal. Ministry of Forest and Soil Conservation, Department of Plant Resources, Thapathali, Kathmandu.

International Trade Centre (ITC). 2007. Export Potential Assessment in Nepal. Trade and Export Promotion Centre, Kathmandu.

IRG. 2006. Role of natural products in resource management, poverty alleviation, and good governance: A case study of jatamansi and wintergreen value chain in Nepal. International Resource Groups. Washington DC.

JABAN. 2014. Medicinal and Medicinal Plants Stakeholders Directory. Jadibuti Association of Nepal (JABAN), Nepalgunj.

Jenisch, T. and Thomas, P. 2011. Medicinal and Aromatic Plants Poverty Impact Assessment (PIA) of proposed Trade Support Measures in Nepal’s Medicinal and Aromatic Plants Sector. Deutsche Gesellschaft für Internationale Zusammenarbeit (GIZ) GmbH, Kathmandu.

Jha, P.K., Karmacharya, S.B., Chhetri, M.K., Thapa, C.B. and Shrestha, B.B. 2008. Medicinal Plants in Nepal: an Anthology of Contemporary Research. Ecological Society (ECOS), Kathmandu.

Kafle, G., Bhattarai, I., Shrestha, A.K. and Siwakoti, M. 2018. Consumers Caring Health with End Products (Ayurvedic Medicines) Containing Neopicrorhiza scrophulariiflora in Nepal. International Journal of Ayurvedic Medicine 9(2): 123-132.

Kafle, G., Bhattarai, I., Shrestha, A.K. and Siwakoti, M. 20+18. Why do patients choose to consume Ayurvedic Medicines in Nepal? An exploratory study. International Journal of Ayurvedic Medicine 9(4): 250-257.

Kala, C.P. 2003. Commercial exploitation and conservation status of high value medicinal plants across the borderline of India and Nepal in Pithoragarh. The Indian Forester 129(1): 80–84.

Kanel, K.R. 2000. Analysing policy for poverty alleviation: an example from non-timber forest product subsector. Banko Janakari 10(2): 3-8.

Karki, M.B. 2000. Commercialization of natural resources for sustainable livelihoods: the case study of forest products. In: M. Banskota, T.S. Papola, and J. Richter (Eds.), Growth, Poverty Alleviation and Sustainable Resource Management in the Mountain Areas of South Asia. Proceedings of the International Conference held from 31 January – 4 February 2000 in Kathmandu, Nepal. Deutsche Stiftung für internationale Entwicklung Zentralstelle für Ernährung und Landwirtschaft, pp. 293–320.

Karki, M. 2005. The organic production of medicinal and aromatic plants: A strategy for improved value-addition and marketing of products from the Himalayas. In: Y. Aumeeruddy-Thomas, M. Karki, K. Gurung and D. Parajuli (Eds.) Himalayan Medicinal and Aromatic Plants, Balancing Use and Conservation. Proceedings of the Regional Workshop on Wise Practices and Experiential Learning in Conservation and Management of Himalayan Medicinal Plants, December 15–20, 2002. His Majesty’s Government of Nepal, Ministry of Forests and Soil Conservation, Kathmandu, pp. 56–69.

Karki, M., Tiwari, B.K., Badoni, A. and Bhattarai, N., 2005. Creating Livelihoods and Enhancing Biodiversity-rich Production Systems Based on Medicinal and Aromatic Plants: Preliminary Lessons from South Asia. Acta Horticulturae 678: 37-43.

Karki, M. and Williams J.T. 1999. Priority Species of Medicinal Plants in South Asia. Report of an Expert Consultation on Medicinal Plants Species Prioritization for South Asia held on 22-23 September 1997, New Delhi, India. MAPPA/IDRC, New Delhi.

K.C., S. 2003. Study of Use and Marketing Potential of Medicinal Plants in Chitrasen Community Forest, Chitwan, Nepal. MSc Thesis. Natural Resource Management. Pokhara University, Pokhara.

K.C., A. 2004. Extraction of NTFPs from the community forest: analysis of benefit distribution pattern in household level. Journal of Forest and Livelihood 4(1): 38-43.

K.C., R. 2014. Prominent Non-Wood Forest Products of Terai and Siwalik Regions in Nepal. Food and Agriculture Organization, Kathmandu.

Kesari, V.J. undated. Promotion and Trade of Tropical NTFPs in Central Terai of Nepal.

Khanal, S., Shakya, N., Nepal, N. and Pant, D. 2014. Swertia chirayita: The Himalayan Herb. International Journal of Applied Sciences and Biotechnology 2(4): 389-392.

Khanal, S.C., Poudel, A. and Subedi, B.P. undated. FAO Forests and Beauty Case Studies Nepal: Spikenard. Regional assessment of NTFPs related to the cosmetics and fragrances sector. Non-Timber Forest Products Exchange Programme (NTFP-EP), and Food and Agriculture Organization of the United Nations (FAO), Kathmandu.

Kirkpatrick, Col. 1811. Account of the Kingdom of Nepaul, being the substance of observations made during a mission to that country in the year 1793. Asian Educational Services, New Delhi. Reprint 1986. 388 pp.

Koirala, R.D. and Khaniya, B.N. 2009. Present Status of Traditional Medicines and Medicinal & Aromatic Plants Related Resources & Organizations in Nepal. Nepal Health Research Council, Ramshah Path, Kathmandu.

Koirala, P.N., Pyakurel, D. and Gurung K. 2013. Orchids in Rolpa district of Western Nepal: Documentation, stock, trade and conservation. Banko Jankari 20 (2): 1-13.

Kunwar, R.M. 2002. Some threatened medicinal and aromatic plants: Status, trade and management practice in Dolpa, Nepal. Journal of Natural History Museum 21: 173-186.

Kunwar, S.C., Ansari, A.S. and Luintel, H. 2009. Non-timber forest products enterprise development: regulatory challenges in the Koshi Hills of Nepal. Journal of Forest and Livelihood 8(2): 39-50.

Kunwar, R.M., Burlakoti C. and Lakhey, P.B. 2008. Status, Use and Trade of Sapindus mukorossi, Swertia chirayita, Valeriana jatamansi and Zanthoxulum armatum Medicinal Plants in Far-west Nepal. In: The Fifth National Conference on Science and Technology, Kathmandu, Nov. 10-12, 2008, Nepal Academy of Science and Technology, Kathmandu, pp. 133-134.

Kunwar, R.M. and Duwadee, N.P.S. 2003. Ecology and economy of NTFPs in Nepal: a case study from Dolpo and Jumla districts, Nepal. Botanica Orientalis 3: 89—97.

Kunwar, R.M., Mahat, M., Acharya, R.P. and Bussmann, R.W. 2013. Medicinal plants, traditional medicine, markets and management in far-west Nepal. Journal of Ethnobiology and Ethnomedicine 9: 24.

Kunwar, R.M., Rimal, B., Sharma, H.P., Poudel, R.C., Pyakurel, D., Tiwari, A., Magar, S.T., Karki, G., Bhandari, G.S., Pandey, P. and Bussmann, R.W. 2021. Distribution and habitat modeling of Dactylorhiza hatagirea (D. Don) Soo, Paris polyphylla Sm. and Taxus species in Nepal Himalaya. Journal of Applied Research on Medicinal and Aromatic Plants 20: 100274.

Kurumbang, N.P. 2003. Ecological study, harvesting and trade of some medicinal plants in Shey-Phoksundo National Park and its buffer zone, Dolpa, Nepal. In: B. Pant and S.K. Ghimire (Eds.) 2006. Abstracts: MSc Dissertation and PhD thesis, Central Department of Botany, Tribhuvan University, Kathmandu.

Kurumbang, N.P. and Ghimire, G.P.S. 2004. Harvesting and Trade of some medicinal plants in the Buffer Zone of Shey-Phoksundo National Park, Dolpa, Nepal. In: Proc. IV National Conference on Science and Technology, RONAST, Kathmandu pp. 723-731.

Lafranchi, S. 1998. Non-timber forest products and income generating opportunities in the Achham and Dailekh Districts: market potentialities and resource management aspects. HELVETAS, Kathmandu. 49pp.

Lama, Y.C., Ghimire, S.K. and Aumeeruddy-Thomas, Y. 2001. Medicinal Plants of Dolpo: Amchis Knowledge and Conservation. People and Plants Initiative. WWF Nepal Program, Kathmandu. 150 pp.

Lamichhane, D. and Karna, N.K. 2009. Harvesting methods of Cinnamomum tamala leaves in private land: a case study from Udayapur district, Nepal. Banko Janakari 19(2): 20-24.

Larsen, H.O. 1999. Commercial collection of Nardostachys grandiflora D.C. in Chaudabise Valley, Jumla District, Nepal: local management systems and sustainability. MSc thesis, Royal Veterinary and Agricultural University, Copenhagen. 135 pp.

Larsen, H.O. 2000. Considerations on medicinal and aromatic plant collection in Nepal: myths and facts. In: Proceedings of the Third Regional Workshop on Community Based NTFP Management, Kathmandu. South and East Asian Countries NTFP Network (SEANN). pp. 133-149.

Larsen, H.O. 2002. Commercial Medicinal Plant Extraction in the Hills of Nepal: Local Management System and Ecological Sustainability. Environmental Management 29(1): 88–101.

Larsen, H.O. and Olsen, C.S. 2007. Unsustainable collection and unfair trade? Uncovering and assessing assumptions regarding Central Himalayan medicinal plant conservation. Biodiversity and Conservation 16: 1679–1697.

Larsen H.O. and Olsen C.S. 2008. Towards Valid Non-Detrimental Findings for Nardostachys grandiflora. Paper presented at the International Expert workshop on CITIES non-detriment Findings, November, Cancun Centre, Cancun, Mexico.

Larsen, H.O., Olsen, C.S., and Boon, T.E. 2000. The non-timber forest policy process in Nepal: actors, objectives and power. Forest Policy and Economics 1(3): 267-281.

Larsen, H.O. and Smith, P.D. 2004. Stakeholder perspectives on commercial medicinal plant collection in Nepal: poverty and resource degradation. Mountain Research and Development 24(2): 141–148.

Larsen, H.O., Smith, P.D. and Olsen, C.S. 2005. Nepal's conservation policy options for commercial medicinal plant harvesting: stakeholder views. Oryx 39(4): 435–441.

Luitel, L.R. and Pathak, M., 2013. Documentation of Medicinal and Aromatic Plants of Dhorpatan Hunting Reserve, Western Nepal. Journal of the Department of Plant Resources 35: 36-43.

Maharjan, P. 2000. Community Based Natural Resource Management: Medicinal and Aromatic Plants in Nepal. In: Proceedings of Nepal-Japan Joint Symposium 2000.

Maharjan, P. 2007. Commercialization of NTFPs: MAPs in Mid-Western Development Zone of Nepal for Rural Income Generation - an Experience of BDS-MAPs Project. In: Abstracts: National Seminar on Sustainable Use on Biological Resources with the special theme: Medicinal and Aromatic Plants. April 22-23, 2007, Pokhara, pp. 19-20.

Malla, S.B., 1994. Medicinal herbs in the Bagmati Zone. ADPI Series No. 8, International Centre for Integrated Mountain Development, Kathmandu. 85 pp.

Malla, S.B., Shakya, P.R., Karki, B.R., Mortensen, T.F. and Subedi, N.R. 1999. A study on non-timber forest products in Bajura District. CARE Nepal, Kathmandu. 34 pp.

Malla, S.B., Shakya, P.R., Rajbhandari, K.R., Bhattarai, N.K. and Subedi, M.N. 1995. Minor forest products (NTFPs) of Nepal: General status and trade. Forest Resource Information System Project Paper No. 4. HMGN/FINNIDA, Kathmandu. 27pp.

Manandhar, N.P. 2002. Plants and People of Nepal. Timber Press, Oregon.

Maraseni, T.N., Shivakoti, G.P., Cockfield, G. and Apan, A. 2006. Nepalese Non-timber: An Analysis of the Equitability of Profit Distribution across a Supply Chain to India. Small-scale Forest Economics, Management and Policy 5(2): 191–206.

Messerschmidt, D.A. and Hammett, A.L. 1997. Local knowledge of alternative forest resources: Its relevance for resource management and economic development. Journal of Sustainable Forestry 7: 21–55.

Mulliken, T.A. 2000. Implementing CITES for Himalayan Medicinal Plants Nardostachys grandiflora and Picrorhiza kurrooa. TRAFFIC Bulletin 18(2): 63-72.

Mulliken, T. and Crofton, P. 2008. Review of the Status, Harvest, Trade and Management of Seven Asian CITES-listed Medicinal and Aromatic Plant Species. Bundesamt für Naturschutz (BfN) Federal Agency for Nature Conservation, Bonn, Germany.

Nepal Environment Watch Initiative (NEWI) 2011. Identification and Market Study of Tradable and Economically Important Medicinal and Aromatic Plants of Western Nepal. A report submitted to Department of Plant Resources, Thapathali, Kathmandu.

Nepali, N., Pyakurel, D. and Regmi, K. 2009. Subsector Analysis for High Value Crops, Livestock and Non-Timber Forest Products (NTFPs) and Sustainable Intervention Strategy in Dudh Koshi Sub Basin Area, Solukhumbu. Unpublished report. Kathmandu: IDE Nepal and WWF Nepal.

New Era. 1992. Non-timber forest products commercialisation feasibility study: country report Nepal. New Era, Kathmandu. 111 pp.

Ojha, H.R. 2000. Current policy issues in NTFP development in Nepal. Asia Network for Small-scale Bio-resources (ANSAB), Kathmandu.

Ojha, H.R. 2001. Commercial use of non-timber forest products: Can the poor really get benefits? Journal of Forestry and Livelihood 1(1): 19-21.

Ojha, H. and Bhattarai, B. 2003. Learning to manage a complex resource: a case of NTFP assessment in Nepal. International Forestry Review 5: 118–127.

Ojha, H.R., Binayee, S.B. and Acharya, R.P. 2000. NTFP based enterprise options for forest user groups in Bungadovan and Khunkani VDCs, Baglung. A synthesis of field trip observations and future plan for NTFP enterprise development. Asia Network for Small-scale Bioresources, Kathmandu. 16pp.

Ojha, H.R., Subedi, B.P. and Dangal, S.P. 2000. Management of Non-Timber Forest Products: Recent Innovations in Resource Assessment and Sustainable Harvesting. A paper prepared for the International Seminar on Harvesting of Non-Timber Forest Products, October 2-8, Izmir, Turkey.

Ojha, H.R., Subedi, B.P. and Dangal, S.P. 2002. Management of non-timber forest products: recent innovations in resource assessment and sustainable harvesting. Discussion Paper, ForestAction Nepal, Kathmandu.

Olsen, C.S. 1995. Medicinal plants in Gorkha District: a brief introduction to resources, constraints and possibilities. In Yadav, S.K. and Stoian, D. (Eds.), Medicinal and aromatic plants in Gorkha District: how to promote their utilisation and marketing, District Forest Office, Gorkha, pp. 11-16.

Olsen, C.S. 1997a. Commercial non-timber forestry in central Nepal: emerging themes and priorities. Royal Veterinary and Agricultural University, Department of Economics and Natural Resources, Copenhagen. PhD dissertation, 187 pp.

Olsen, C.S. 1997b. Proposed listing of Nardostachys grandiflora DC. and Picrorhiza kurrooa Royle ex Benth. on CITES Appendix II: An evaluation. Background paper to the Danish CITES Authority in preparation for the 10th Conference of the Parties. Royal Veterinary and Agricultural University, Unit of Forestry, Copenhagen. 17 pp.

Olsen, C.S. 1997c. A qualitative assessment of the sustainability of commercial non-timber forest product collection in Nepal. Forestry Discussion Paper 12, Royal Veterinary and Agricultural University, Copenhagen. 30pp.

Olsen, C.S. 1998. The trade in medicinal and aromatic plants from central Nepal to northern India. Economic Botany 52(3): 279-292.

Olsen, C.S. 2002. The trade in medicinal plants from Nepal: status and possible improvements. In: Anon. (Ed.) Medicinal plants: a global heritage. International Development Research Centre, Delhi, pp. 131-141.

Olsen, C.S. 2005a. Quantification of the trade in medicinal and aromatic plants in and form Nepal. Acta Horticulture, 678: 29-35.

Olsen, C.S. 2005b. Trade and conservation of Himalayan medicinal plants: Nardostachys grandiflora DC. and Neopicrorhiza scrophulariiflora (Pennell) Hong. Biological Conservation 125: 505–14.

Olsen, C.S. 2005c. Valuation of commercial central Himalayan medicinal plants. Ambio 34: 607-610.

Olsen, C.S. and Bhattarai, N. 2005. A Typology of Economic Agents in the Himalayan Plant Trade. Mountain Research and Development 25(1): 37-43.

Olsen, C.S. and Helles, F. 1997a. Making the poorest poorer: policies, laws and trade in medicinal plants in Nepal. Journal of World Forest Resource Management 8(2): 137-158.

Olsen, C.S. and Helles, F. 1997b. Medicinal plants, markets and margins in the Nepal Himalaya: Trouble in Paradise. Mountain Research and Development 17(4): 363-374.

Olsen, C.S. and Helles, F. 2009. Market efficiency and benefit distribution in medicinal plant markets: empirical evidence from South Asia. International Journal of Biodiversity Science & Management 5(2): 53-62.

Olsen, C.S. and Larsen, H.O. 2003. Alpine medicinal plant trade and Himalayan mountain livelihood strategies. The Geographical Journal 169: 243-254.

Olsen, C.S. and Treue, T. 2003. Analysis of trade in non-timber forest products. In: Helles, F., Strange, N. and Wichmann, L. (Eds.) Recent accomplishments in applied forest economics research, Forestry Sciences Series no. 74, Kluwer Academic Publishers, Dordrecht, pp. 227-239.

Pande, R.K. 2006. Potential Non-Timber Forest products (NTFPs) and Their Contribution to the Livelihood of Local People and Hal Khoriya Collaborative Forest of Bara Districts. MSc Dissertation, Institute of Forestry, Tribhuvan University, Pokhara.

Pandey, N. 1994. Study of Nepalese Swertia L. In: Proc. II National Conference on Science and Technology, RONAST, Kathmandu, pp. 576.

Pandey, S.S., Subedi, B.P. and Dhungana, H. 2010. Economic potential of forest resources of Nepal. Banko Janakari 20(2): 48–52.

Pandit, B.H. 2008. Economics of Non-Timber Forest Production Promotion and Marketing: A case study from Malekhukhola Watershed of Dhading District, Nepal. The Initiation 2(1): 145-156.

Pandit, B.H., Albano, A. and Kumar, C. 2009. Community-based forest enterprises in Nepal: An analysis of their role in increasing income benefits to the poor. Small-scale Forestry 8: 447–462.

Pandit, B.H. and Kumar, C. 2010. Factors influencing the integration of non-timber forest products into field crop cultivation: a case study from eastern Nepal. Journal of Sustainable Forestry 29: 671–695.

Pandit, B.H., Subedi, R. and Kumar, C. 2007. Profitability Analysis of Non-Timber Forest Products Marketing in Eastern Hills of Nepal: Looking for an Alternative Marketing Approach to Benefit the Poor. The Initiation 1: 16-28.

Pandit, B.H. and Thapa, G.B. 2004. Poverty and resource degradation under different common forest resource management systems in the mountains of Nepal. Society & Natural Resources 17: 1–16.

Pandit, B.H. and Thapa, G.B. 2003. A tragedy of non-timber forest resources in the mountain commons of Nepal. Environmental Conservation 30(3): 283-292.

Pandit, B.H., Thapa, G.B. and Zoebisch, M. 2005. Promoting and Marketing of Cinnamon Tree Products in Palpa District of Nepal. In Marketing and agroforestry development in Vietnam's uplands, T.D. Vien et al. (Eds). Agriculture Publishing House, Hanoi, Vietnam, pp. 256-280.

Pant, B., Pradhan, S., Paudel, M.R., Shah, S., Pandey, S., Joshi, P.R., Kim, J., Kim, Y.J. and Park, S.J. 2019. Various culture techniques for the mass propagation of medicinal orchids from Nepal. Acta Horticulturae 1262: 109-124.

Parajuli, D.P. 1998. Cultivation of Cinnamomum tamala in marginal lands for greater income at Palpa district. Banko Janakari 8(1): 24-32.

Parajuli, D.P., Gyanwali, A.R. and Shrestha, B.M. 1998. Manual of Important NTFPs in Nepal. Institute of Forestry, Pokhara. 74pp.

Paudel, M. 2007. Non-timber forest products from community forestry practices, problems and prospects for livelihood strategy in Jumla. Banko Janakari 17(2): 45-54.

Paudel, S. and Smith-Hall, C. 2022. Empirically derived typologies of environmental product periodic markets and retailers. Environment, Sustainability and Development 24: 13111–13136.

Paudel, A., Subedi, B.P., Gyawali, S., Thapa, G.K. and Sharma, M.B. 2009. Value chain analysis of non-timber forest products in Baglung district, Nepal. Banko Janakari 19(2): 33-41.

Phoboo, S., Devkota, A. and Jha, P.K. 2006. Medicinal Plants in the Nepal – An Overview. In: Jha, P.K., Karmacharya, S.B., Chhetri, M.K., Thapa, C.B. and Shrestha, B.B. (Eds.) Medicinal Plants in Nepal: an Anthology of Contemporary Research. Ecological Society (ECOS), Kathmandu, pp 1-24.

Phoboo, S. and Jha, P.K. 2010. Trade and Sustainable Conservation of Swertia chirayita (Roxb. ex Fleming) H. Karst in Nepal. Nepal Journal of Science and Technology 11: 125-132.

Phuyal, N., Jha, P.K., Raturi, P.P. and Rajbhandary, S. 2019. Zanthoxylum armatum DC.: Current knowledge, gaps and opportunities in Nepal. Journal of Ethnopharmacology 229: 326-341.

Piya, L., Maharjan, K.L., Joshi, N.P. and Dangol, D.R. 2011. Collection and Marketing of Non-Timber Forest Products by Chepang Community in Nepal. The Journal of Agriculture and Environment 12: 10-21.

Pokharel, N. 1999. Herbs production, processing and their sales in Nepal: a case study of Herbs Production and Processing Company Limited with special reference to the products of Chirpine. Central Department of Economics, Kathmandu. MA Thesis, 69pp + annexes.

Pokharel, S.B. 2013. Chepang and Chiuri: Opportunity of Paradigm Shift from Indigenous Knowledge and Practice to Trade, and Climate Change Consequences in Livelihood. In: Balla M.K., Rayamajhi, S. and Singh, A. (Eds.) Proceedings of the International Conference on Forests, People, and Climate: Changing Paradigms, Institute of Forestry, Pokhara, pp. 128-138.

Pokharel, B., Paudel, D., Branney, P., Khatri, D.B. and Nurse, M. 2006. Reconstructing the concept of forest-based enterprise development in Nepal: towards a pro-poor approach. Journal of Forest and Livelihood 5: 53-65.

Poudel, K.L. 2007. [Trade Potentility and Ecological Analysis of NTFPs in Himalayan Kingdom of Nepal](https://repository.unm.edu/dspace/bitstream/1928/3300/1/KrishnaPoudel_EcolAnalysisNTFP.pdf). Himalayan Research Papers Archive, Nepal Study Center, University of New Mexico.

Poudel, D.D. 2008. Management of Eight ‘Ja’ for Economic Development of Nepal. [Journal of Comparative International Management](https://journals.lib.unb.ca/index.php/JCIM/index) 11(1): 15-27.

Poudeyal, M.R., Meilby, H., Shrestha, B.B. and Ghimire, S.K. 2019. Harvest effects on density and biomass of Neopicrorhiza scrophulariiflora vary along environmental gradients in the Nepalese Himalayas. Ecology and Evolution 9(13): 7726-7740.

Poudeyal, M.R., Pyakurel, D., Rana, S.K., Meilby, H., Paneru, Y.R. and Ghimire, S.K. 2021. Does resource availability coincide with exploitation patterns? Inference from distribution and trade of Neopicrorhiza scrophulariiflora (Pennell) D.Y. Hong in the Nepalese Himalayas. Journal of Applied Research on Medicinal and Aromatic Plants 22: 100292.

Practical Solution Consultancy Nepal. 2014a. Status Mapping and Trade Analysis of NTFPS/MAPS in Trans-Himalayan Region of Nepal. Final report submitted to Government of Nepal, Ministry of Forest and Soil Conservation, Department of Plant Resources, Thapathali, Kathmandu.

Practical Solution Consultancy Nepal. 2014b. Report of Value Chain Analysis of Ritha (Soapnut) in Darchula and Baitadi Districts. Government of Nepal, Ministry of Forest and Soil Conservation, Department of Plant Resources, Thapathali, Kathmandu.

Practical Solution Consultancy Nepal. 2014c. Status mapping and Feasibility Study for Cultivation Promotion of Wild MAPs/NTFPs in the Chure and Adjacent Areas of Makwanpur, Bara, Parsa, Rautahat, Sarlahi, Sunsari, Morang and Jhapa Districts. Final report submitted to Government of Nepal, Ministry of Forest and Soil Conservation, Department of Plant Resources, Thapathali, Kathmandu.

Pradhan, N. and Manandhar, V. 2000. Conservation and Trade Pattern of Medicinal Plants of Tehrathum District, Nepal. Proceedings of Nepal-Japan Joint Symposium 2000, pp 317-321.

Putterman D. and Koontz A. undated. Biotechnology, Biodiversity and Natural Resources Management: Notes from the Field on the Role of Value-Adding Technology. EnterpriseWorks Worldwide (formerly Appropriate Technology International), Washington DC.

Pyakurel, D. 2009. Assessment and profile preparation of traded NTFPs of Jumla district (Report). Western Uplands Poverty Alleviation Project (WUPAP), Nepalgunj, Banke.

Pyakurel, B. 2011. Prospects of Promoting NTFPs for Livelihood Improvement. The Initiation 4: 46-55.

Pyakurel, D. and Baniya, A. 2011. NTFPs: Impetus for Conservation and Livelihood support in Nepal. A Reference Book on Ecology, Conservation, Product Development, and Economic Analysis of Selected NTFPs of Langtang area in the Sacred Himalayan Landscape. WWF Nepal, Kathmandu.

Pyakurel, D. and Chapagain, S. 2015. Diagnosing Barriers for Value Chain development of Chireeta and Pig. Unpublished report. Kathmandu: White Lotus Centre and Asian Development Bank.

Pyakurel, D. and Oli, B.R. 2012. NTFPs / MAPs Business Promotion Strategy (2012-2016): From Private Sector Perspective. Unpublished report, Kathmandu: FNCCI-AEC/NEHHPA.

Pyakurel, D. and Oli, B.R. 2013. Market Study of Tradable and Economically Important Medicinal and Aromatic Plants of Eastern Nepal. A report submitted to Department of Plant Resources, Kathmandu.

Pyakurel, D., Oli, B.R. and Thapa, M.S. 2014. Feasibility Study of Commercially Valuable Medicinal and Aromatic Plants of Far Western Development Region, Nepal. A report submitted to Department of Plant Resources, Kathmandu.

Pyakurel, D. and Panthi, S. 2015. Trade of High Valued MAPs from Nepal to China. A report submitted to Department of Plant Resources, Kathmandu.

Pyakurel, D., Poudel, S. and Dahal, M. 2013. Identification and Market Study of Tradable and Economically Important Medicinal and Aromatic Plants of Mid-Western Nepal. A report submitted to Department of Plant Resources, Kathmandu.

Pyakurel, D. Sharma, I.B. and Smith-Hall, C. 2018. Patterns of change: The dynamics of medicinal plant trade in far-western Nepal. Journal of Ethnopharmacology 224: 323-334.

Pyakurel, D., Smith-Hall, C., Bhattarai-Sharma, I. and Ghimire, S.K. 2019. Trade and conservation of Nepalese medicinal plants, fungi, and lichens. Economic Botany 73(4): 505-521.

Rajbhadari, K.R. 1994. A Bibliography of the Plant Science of Nepal. Nepal Press, Kathmandu. 247pp.

Rajbhandary, T.K. and Bajracharya, J.M. 1994. National status paper on NTFPs: Medicinal and aromatic plants. In: Pradhan, J. and Maharjan, P. (Eds.), Proceedings of the national seminar on non-timber forest products: Medicinal and aromatic plants. Ministry of Forest and Soil Conservation and Herbs Production & Processing Co. Ltd, Kathmandu, pp. 8-15.

Rana, S.K., Rana, H.K., Ranjitkar, S., Ghimire, S.K., Gurmachhan, C.M., O'Neill, A.R. and Sun, H. 2020. Climate-change threats to distribution, habitats, sustainability and conservation of highly traded medicinal and aromatic plants in Nepal. Ecological Indictors 115: 106435.

Rasaily, N.K. 2003. Production, Processing and Marketing of Potential Non-Timber Forest Products (NTFPs) in Parbat, Nuwakot and Pyuthan Districts. Micro-Enterprise Development Programme (MEDEP), Lalitpur.

Rasul, G., Choudhary, D., Pandit, B.H. and Kollmair, M. 2012. Poverty and Livelihood Impacts of a Medicinal and Aromatic Plants Project in India and Nepal: An Assessment. Mountain Research and Development 32(2): 137-148.

Rawal, R.B. 1994. Present Status of Medicinal Plants in Nepal. In: Proc. II National Conference on Science and Technology, RONAST, Kathmandu, pp. 834.

Rawal, J.R. 2004a. Medicinal Plants Trade between Nepal and India: Regulatory Framework, Implementation Problems and Solutions. In: N.K. Bhattarai and M. Karki (Eds). Local Experience based National Strategy for Organic Production and Management of MAPs/NTFPs in Nepal. IDRC/MAPPA, Kathmandu, pp. 176–183.

Rawal, R.B. 2004b. Marketing Nepal’s Non-Timber Forest Products: Challenges and Opportunities. In: N.K. Bhattarai and M Karki (Eds). Local Experience based National Strategy for Organic Production and Management of MAPs/NTFPs in Nepal. IDRC/MAPPA, Kathmandu, pp. 150–164.

Rawal, R.B., Acharya, B. and Subedi, B.P., 2001. Jaributi marketing and policy issues in Nepal. In: Proceedings of the National Workshop on Non-Timber Forest Products: Jaributi. February 22, 2001. Kathmandu, pp. 30-38.

Rawal, R.B., Pradhan, J. and Bajracharya, J.M. 1996. Commercial utilization of medicinal and aromatic plants. In: Jha, P.K. et al. (Eds.) Environment and Biodiversity in the context of South Asia. Proceedings of the Regional Conference on Environment and Biodiversity, 7-9 March 1994. Ecological Society, Kathmandu, pp 256-259.

Rawal, R.B., Prasad, R.R. and Adhikary, S.R. 1994. A review and analysis of national policy in the development of NTFPs: medicinal and aromatic plants. In: Pradhan J. and Maharjan P. (Eds.), Proceedings of the national seminar on non-timber forest products: medicinal and aromatic plants, Kathmandu, September 11-12, 1994. Ministry of Forests and Soil Conservation, and Herbs Production and Processing Co. Ltd., Kathmandu, pp. 49-54.

Rawal, D.S., Sijapati, J., Rana, N., Rana, P., Giri, A. and Shrestha, S. 2009. Some high value medicinal plants of Khumbu region Nepal. Nepal Journal of Science and Technology 10: 73–82.

Rijal, A., Smith-Hall, C. and Helles, F. 2011. Non-timber forest product dependency in the Central Himalayan foot hills. Environment, Development and Sustainability 13: 121–140.

Rokaya, M.B., Münzbergová, Z., Shrestha, M.R. and Timsina, B. 2012. Distribution patterns of medicinal plants along an elevational gradient in central Himalaya, Nepal. Journal of Mountain Science 9: 201–213.

Roy, R. 2010. Contribution of NTFPs [Non-Timber Forest Products] to Livelihood in Upper Humla, Nepal. A dissertation submitted in partial fulfilment of the requirements for the degree of Doctor of Philosophy in Natural Resources Management. Asian Institute of Technology, School of Environment, Resources and Development, Bangkok.

Rupantaran Nepal. 2014. Value chain analysis of selected forest based products of rapti area. Rupantaran Nepal and Multi Stakeholder Forestry Programme, Kathmandu.

Saxer, M. 2009. Herbs and Traders in Transit: Border Regimes and the Contemporary Trans-Himalayan Trade in Tibetan Medicinal Plants. Asian Medicine, 5 (2): 317-339.

Shahi, D.P. 2002. Trade systems and feasibility of cultivation/domestication of MAPs in the southern buffer zone of Shey Phoksundo National park, Dolpa district, Nepal. In: Thomas, Y., Karki, M., Gurung, K. and Parajuli, D. (Eds.) Himalayan Medicinal and Aromatic Plants, Balancing Use and Conservation. Proceedings of the Regional Workshop on Wise Practices and Experimental Learning in Conservation and Management of Himalayan Medicinal Plants, Kathmandu, pp. 506-510.

Sharma, P. 1995. Non-wood forest products and integrated mountain development: observations from Nepal. Non-Wood Forest Products 3: 157-166.

Sharma, U.R. 2007. Medicinal and Aromatic Plants: A Growing Commercial Sector of Nepal. **The Initiation** 1: 4-8.

Sharma, P.P. 2009. Zanthoxylum armatum (Timur) and its contribution to household income: a case study from Myagdi district. MSc Dissertation, Institute of Forestry, Tribhuvan University, Pokhara.

Sharma, U.R. 2014. A Review and Analysis of Policies on Non-Wood Forest Products of Nepal. Report TCP/NEP/3403), FAO, Kathmandu, The Project on Sustainable Management and Development of NWFPs in Tarai and Siwalik Regions of Nepal.

Sharma, U.R., Malla, K.J. and Uprety, R.K. 2004. Conservation and management efforts of medicinal and aromatic plants in Nepal. Banko Janakari 14: 3–11.

Sharma, P. and Shrestha, N. 2011. Promoting Exports of Medicinal and Aromatic Plants (MAPs) and Essential Oils from Nepal. South Asia Watch on Trade, Economics and Environment (SAWTEE). Submitted to WTO/EIF Support Programme Deutsche Gesellschaft für Internationale Zusammenarbeit (GIZ), Kathmandu.

Sherchan, R., Chapagain, N.R. and Chhetri, M. 2005. Distribution, Conservation Practices and Trade of Yarsagumba in Manang District of Annapurna Conservation Area, Nepal. Forestry (Journal of Institute of Forestry, Nepal) 13: 99-107.

Sherpa S. 2001. The High Altitude Ethnobotany of the Walung People of Walangchung Gola, Kanchenjunga Conservation Area, East Nepal. MSc Dissertation, Central Department of Botany, Tribhuvan University, Kirtipur, Kathmandu.

Shrestha, U.B. and Bawa, K.S. 2013. Trade, harvest and conservation of caterpillar fungus (Ophiocordyceps sinensis) in the Himalayas. Biological Conservation 159: 514-520.

Shrestha, U.B. and Bawa, K.S. 2014a. Economic contribution of Chinese caterpillar fungus to the livelihoods of mountain communities in Nepal. Biological Conservation 177: 194–202.

Shrestha, U.B. and Bawa, K.S. 2014b. Impact of climate change on potential distribution of Chinese caterpillar fungus (Ophiocordyceps sinensis) in Nepal Himalaya. PLoS One 9(9).

Shrestha, U.B. and Bawa, K.S. 2015. Harvesters’ perceptions of population status and conservation of Chinese caterpillar fungus in the Dolpa region of Nepal. Regional Environmental Change 15: 1731–1741.

Shrestha, K.K. and Ghimire, S.K. 1996. Diversity, Ethnobotany and Conservation Strategy of Some Potential Medicinal and Aromatic Plants of Taplejung in Tamur Valley. Asia Network for Small-scale Agricultural Biotechnologies, Kathmandu, 32pp.

Shrestha, K.K., Ghimire, S.K., Gurung, T.N., and Lama, Y.C. 1997. Conservation of Plant Resources, Community Development and Training in Applied Ethnobotany at Shey-Phoksundo National Park and its Buffer-zone, Dolpa. First year. WWF Nepal Program, Kathmandu.

Shrestha, B.B. and Jha, P.K. 2010. Life History and Population Status of the Endemic Himalayan Aconitum naviculare. Mountain Research and Development 30(4): 353-364.

Shrestha, U.B., Lamsal, P., Ghimire, S.K., Shrestha, B.B. Dhakal, S., Shrestha, S. and Atreya, K. 2022. Climate change-induced distributional change of medicinal and aromatic plants in the Nepal Himalaya. Ecology and Evolution 12(8): e9204.

Shrestha, K.K., Poudel, R.C., Ghimire, S.K., Shrestha, S., Rijal, B. and Basnet, R. 2015. Priority Plant Species of Nepal for DNA Barcoding (with Focus on Highly Traded and Protected Species). Barcode of Wildlife Project (BWP), Nepal, National Trust for Nature Conservation, Khumaltar, Lalitpur.

Shrestha, K.K., Sah, J.P. and Ghimire, S.K. 1996. Diversity and conservation strategy of potential medicinal plants in Manang (Gyasumdo valley). King Mahendra Trust for Nature Conservation, Kathmandu, 35 pp.

Shrestha, N. and Shrestha, K.K. 2012. Vulnerability assessment of high-valued medicinal plants in Langtang National Park, Central Nepal. Biodiversity 13(1): 24–36.

Shrestha, U.B. and Shrestha, B.B. 2019. Climate change amplifies plant invasion hotspots in Nepal. Diversity and Distributions 25(10): 1599-1612.

Shrestha, U.B., Shrestha, S., Ghimire, S., Nepali, K. and Shrestha, B.B. 2014. Chasing Chinese Caterpillar Fungus (Ophiocordyceps sinensis) Harvesters in the Himalayas: Harvesting Practice and Its Conservation Implications in Western Nepal. Society & Natural Resources 27(12): 1242-1256.

Shresta, K.N. and Stoian, D. 1995. The forest rules 1995 and their relevance to the utilisation of medicinal and aromatic plants. In: Amatya, S.M. and Stoian, D. (Eds.) Proceedings of the seminar on medicinal and aromatic plants in Gorkha District: how to promote their utilisation and marketing. German Development Service, District Forest Office, Gorkha bazaar, pp. 1-6.

Shrestha, K.K., Tiwari, N.N., Rajbhandari, S., Shrestha, S., Uprety, Y. and Poudel, R.C. 2003. Non-timber Forest Products (NTFPs) in the Critical Bottlenecks and Corridors of Terai Arc- Landscape Nepal: Documentation, Utilization, Trade and People’s Livelihood. WWF-Nepal, Kathmandu.

Shrestha, B., Tsiftsis, S., Chapagain, D.J., Khadka, C., Bhattarai, P., Shrestha, N.K., Kolanowska, M.A. and Kindlmann, P. 2021. Suitability of Habitats in Nepal for Dactylorhiza hatagirea: Now and under Predicted Future Changes in Climate. Plants 10(3): 467.

Shrestha-Acharya, R. 2007. The Non-Timber Forest Products Sector in Nepal: Policy Issues in Plant Conservation and Utilization. MS thesis, Florida International University, Miami.

Shrestha-Acharya, R. and Heinen, J. 2006. Emerging policy issues on non-timber forest products in Nepal. Himalaya 26(1): 51-53.

Sigdel, S.R. 2008. Economy and Ecology of Cordyceps sinensis (Berk.) Sacc. (Yarchagumbu). A case study from Dolpa. In: ABSTRACTS: The Fifth National Conference on Science and Technology, Kathmandu, Nov. 10-12, 2008, Nepal Academy of Science and Technology, Kathmandu, pp. 310-311.

Singh, M.P., Malla, S.B., Rajbhandari, S.B. and Manandhar, A. 1979. Medicinal plants of Nepal: Retrospects and prospects. Economic Botany 33(2): 185-198.

Stoian, D. and Yadav, S.K. 1995. Medicinal plants and community forestry in Gorkha District: present status and future perspectives. In: Yadav, S.K. and Stoian, D. (Eds.) Medicinal and aromatic plants in Gorkha District - how to promote their utilisation and marketing. District Forest Office, Gorkha, pp. 2-10.

Subedi, B.P. 1997. Utilization of Non-Timber Forest Products: Issues and strategies for Environmental Conservation and Economic Development. The workshop on utilization of NTFPs for Environmental Conservation and Economic Development in Nepal. ANSAB, Kathmandu.

Subedi, B.P. 1998. Participatory Utilization and Conservation of Medicinal and Aromatic Plants: A Case from Western Nepal Himalaya. A paper prepared for the International Conference in Medicinal Plants, February 16-19, 1998, Bangalore, India.

Subedi, B.P. 1999. Non-Timber Forest Products Sub-Sector in Nepal: Opportunities and Challenges for Linking the Business with Biodiversity Conservation. A paper prepared for the workshop on Natural Resources Management for Enterprise Development in Himalayas, August 19-21, 1999, Nainital, India.

Subedi, B.P. 2000a. Policy and Regulatory Environment for the conservation and Utilization of Himalayan Medicinal Resources in Nepal. A paper prepared for the workshop “Nepal-Japan Joint Symposium on conservation and utilization of the Himalayan Medicinal Resources” organized by Department of Plants Resources, November 8-11, 2000, Kathmandu, pp. 19-26.

Subedi, B.P. 2000b. Strategies for Sustainable Supply of Forest Products for Community-Based Enterprises.

Subedi, B.P. 2002. Economic incentives for Medicinal plants conservation in Nepal: Issues and Options. Proceedings of the regional workshop on Wise practices and Experiential learning in conservation and management of Himalayan medicinal plants, December 15-20, 2002, Kathmandu, pp. 460 – 473.

Subedi, B.P. 2006. Linking Plant-Based Enterprises and Local Communities to Biodiversity Conservation in Nepal Himalaya. Adroit Publishers, New Delhi.

Subedi, M. 2009. Aromatic Plant Trade and Livelihood Strategies in Rural Nepal: A Case of Wintergreen in Dolakha District. Occasional Papers in Sociology and Anthropology 11: 84–103.

Subedi, B.P. 2011. Marketing of Medicinal and Aromatic Plant Products of Nepal in Domestic and International Markets. Lecture notes for the Officers' Training at the Ministry of Forests and Soil Conservation, Kathmandu. MoFSC, Kathmandu.

Subedi, B.P. and Bhattarai, N.K. 2002. Community managed enterprise: Participation of rural people in medicinal and aromatic plants conservation and use. In: M. Karki and R. Johari (Eds.) The Role of Medicinal Plants Industry in Fostering Biodiversity Conservation and Rural Development, International Development Research Centre, pp 89 –95.

Subedi, B.P. and Binayee, S.B. 2000. Linking Conservation to Business and Local Communities: An Approach to Sustainable Management of in situ Biodiversity in Nepal. A paper presented in the 3rd SEANN Workshop on Community-Based Non-timber Forest Products Management, April 7-8, 2000, Kathmandu.

Subedi, B.P., Ghimire, P.L., Koontz, A., Khanal, S.C., Katwal, P., Sthapit, K.R. and Mishra, S.K. 2014. Private Sector Involvement and Investment in Nepal’s Forestry: Status, Prospects and Ways Forward. Study Report, Multi Stakeholder Forestry Programme - Services Support Unit, Babarmahal, Kathmandu.

Subedi, B.P. and Khanal, S.C. 2014. NTFP-based enterprises: learning from Nepal for green and fair value-chain development. Sustainable Forest Management for Multiple Values: A Paradigm Shift. Forest Research Institute, Dehradun, pp. 783-803.

Subedi, A., Kunwar, B., Choi, Y., Dai, Y., Andel, T.V., Chaudhary, R.P., de Boer, H.J. and Gravendeel, B. 2013. Collection and trade of wild-harvested orchids in Nepal. Journal of Ethnobiology and Ethnomedicine 9: 64.

Subedi, B.P. and Ojha, H.M. 2001. Commercial Use of Biodiversity and Equity: Are they compatible? Proceedings of “Sharing local and national experience in conservation of medicinal and aromatic plants in South Asia’’ workshop, Pokhara, 21-23 January 2001, pp 145-162.

Subedi, B.P. and Pandey, S.S. 2011. Cross-border NTFP value chains: Nepal–India. In: R. Kwaschik (Ed.) Cross-Border Value Chains for Non-Timber Forest Products in Four Different Asian Countries, International Network for Bamboo and Rattan (INBAR), Beijing, pp. 8-19.

Tandon, V., Bhattarai, N. K. and Karki, M. 2001. Conservation Assessment and Management Prioritization Report. International Development Research Centre (IDRD), Canada and Ministry of Forest and Soil Conservation, Kathmandu, 197pp.

Thapa, S., Kunwar, R.M., Adhikari, B., Paudel, H.R. and Subedi, S. 2021. Trillium govanianum (Himalayan Trillium): production, distribution, use and conservation in Nepal. Nordic Journal of Botany 39(12): e03356.

Thapa, B.B., Panthi. S., Rai, R.K., Shrestha, U.B., Aryal, A., Shrestha, S. and Shrestha, B. 2014. An assessment of Yarsagumba (Ophiocordyceps sinensis) collection in Dhorpatan hunting reserve, Nepal. Journal of Mountain Science 11(2): 555-562.

Tiwari, N.N., Poudel, R.C. and Uprety, Y. 2004. Study on domestic market of medicinal and aromatic plants (MAPs) in Kathmandu Valley. Kathmandu: Business Development Services-Marketing, Production and Services (BDS-MaPS). Winrock International, Kathmandu.

Tiwari, S., Robinson, J. and Amatya, G. 2004. Promoting Sustainable Livelihoods of Poor and Dalits through Community Based Approaches to Conservation and Sustainable Management of Medicinal and Aromatic Plants in Doti District: Experience of IUCN Nepal. Paper presented in the National Workshop on Local Experience-Based National Strategy for Organic Production and Management of MAPs/NTFPs in Nepal. The Ministry of Forest and Soil Conservation, Kathmandu.

TRAFFIC International. 1999. Implementation of the CITES Appendix II Listing of Jatamansi Nardostachys grandiflora and Kutki Picrorhiza kurrooa. Convention on International Trade in Endangered Species of Wild Fauna and Flora, Tenth meeting of the Plants Committee, Shepherdstown, 11–15 December 2000.

UNEP. 2012, Green Economy Sectoral Study: BioTrade – Harnessing the potential for transitioning to a green economy – The Case of Medicinal and Aromatic Plants in Nepal. United Nations Environment Programme, Kathmandu.

Upadhya, A.V. and Joshi, D.P. 1994. Nepalese Medicinal Plants and Their Role in World Market. In: Proc. II National Conference on Science and Technology, RONAST, Kathmandu, p. 817.

Uprety, Y., Boon, E.K., Poudel, R.C., Shrestha, K.K., Rajbhandari, S., Ahenkan, A. and Tiwari, N.N. 2010. Nontimber forest products in Bardiya district of Nepal: Indigenous Use, Trade, and Conservation. Journal of Human Ecology 30(3): 143-158.

Uprety, Y., Poudel, R.C., Asselin, H., Boon, E.K. and Shrestha, K.K. 2011. Stakeholder Perspectives on Use, Trade, and Conservation of Medicinal Plants in the Rasuwa District of Central Nepal. Journal of Mountain Science 8: 75–86.

Uprety, Y., Poudel, R.C., Gurung, J., Chettri, N. and Chaudhary, R.P. 2016. Traditional use and management of NTFPs in Kangchenjunga Landscape: implications for conservation and livelihoods. Journal of Ethnobiology and Ethnomedicine 12: 19.

Vaidya, B. 2002. Livelihoods and sustainability aspects of non-timber forest products in Gorkha district, Nepal. MSc Dissertation, Agricultural University of Norway, Oslo.

Wagner, A., Kriechbaum, M. and Koch, M.A. 2008. Applied vulnerability assessment of useful plants: a case study of Tibetan medicinal plants from Nepal. Botanische Jahrbücher 127(3): 1–29.

Yadav, Y. 2006. The Role of Non-Timber Forest Product in Livelihood of Local People in Gautam Buddha Community Forest in Kapikvastu District. MSc Dissertation, Institute of Forestry, Tribhuvan University, Pokhara.

Yadav, B.K.V. 2008a. Cultivation and Marketing of Asparagus (Asparagus racemosus) in Sarlahi District. MSc Dissertation, Institute of Forestry, Tribhuvan University, Pokhara.

Yadav, B.K.V. 2008b. Problem and Prospectus of Medicinal and Aromatic Plants' cultivation and marketing (A case study from Sarlahi District). A Special Study Submitted in the Partial Fulfilment of the Requirements for Degree of Masters of Science of Forestry. Tribhuvan University, Institute of Forestry, Pokhara.

Yadav, S. 2008c. Contribution of Non-Timber Forest Products to Local Livelihoods: A Case Study of Navajyoti Community Forest User Group, Udayapur, Nepal. MSc Dissertation, Institute of Forestry, Tribhuvan University, Pokhara.

Yadav, K.R. 2011. Status, Market, Opportunities and Challenges: Yarshagumba in Darchula. The Initiation 4: 138 – 151.

Yadav, S.K. and Stoian, D. 1995. Medicinal and aromatic plants in Gorkha District: how to promote their utilisation and marketing. District Forest Office, Gorkha. 42 pp.

Yonzon, P. 1993a. Raiders of the Park. Himal Jan/Feb: 22-23.

Yonzon, P. 1993b. Jaributi exploration from Langtang National Park. In: Edwards, D.M. and Bowen, M.R. (Eds.) Focus on jaributi. Forest Research and Survey Centre. Occasional Paper 2/93, Kathmandu, pp. 10-11.

# Appendix S5 List of stakeholder representatives (n=29) who provided inputs to the draft roadmap at the “Building a roadmap for sustainable management of commercial medicinal plants in Nepal” workshop, Kathmandu, 24 August 2023

| ***Name*** | ***Institution*** |
| --- | --- |
| Rajendra KC, Sangita Swar, Jwala Shrestha, Ramesh Basnet | Department of Plant Resources |
| Rabindra Maharjan | Department of Forest and Soil Conservation |
| Ajaya Karki | Department of National Parks and Wildlife Conservation |
| Nandalal Raya Yadav | Forest Research and Training Centre |
| Baburaja Amatya | Singhadurbar Vaidhyakhana |
| R. P. Choudhary | Research Centre for Applied Science and Technology |
| Arun Poudyal | Kathmandu Forestry College |
| Thakur Bhandari, Parbata Gautam, Birkha Shahi, Gokarna Chaulagain, Arjun Chapagain | Federation of community Forestry Users Nepal (FECOFUN) |
| Bijendra Basnyat | The Biodiversity Finance Initiative |
| Govinda Ghimire | Federation Of Export Entrepreneurs Nepal (FEEN) |
| Bishnu Bhandari, Debendra Dhakal | Herbal Entrepreneurs Association of Nepal |
| Laiku Lama | Himalayan Herbs Traders Pvt. Ltd. |
| Prem Raj Tiwari | Bio Herbal Products Pvt. Ltd. |
| Sanjay Kumar Jain, Swapnil Jain | Bahubali Herbal Essence & Extracts Pvt. Ltd. |
| Khilendra Gurung | Himalayan Biotrade |
| Prabin Kumar Bastola | Panchatatwa Intl. Pvt. Ltd |
| Sanjib Giri | Dabur Nepal |
| Saurabh Pahari | Himalayan Essence |
| Ramila Bohara | Local Trader - Jumla |
| Reshu Bashyal | Greenhood Nepal |

# Appendix S6 Abridged workshop report: Building a roadmap for sustainable management of commercial medicinal plants in Nepal, Department of Food and Resource Economics, University of Copenhagen

# Introduction

The trade of Himalayan medicinal plant products dates back millennia to export to the Middle East and the Roman Empire. Since then, the international trade in Himalayan medicinal plant products has grown. In Nepal, as many as 300 species are traded annually in tens of thousands of tons worth millions of USD. Evidence shows that past and current attempts at promoting sustainable management in Nepal have not sufficiently ensured sustainable harvesting, leaving at least some species vulnerable to commercial harvesting, thereby threatening rural household incomes, processor supplies and jobs, and government revenues.

This workshop convened central stakeholders in Nepal's medicinal plant production network – harvesters, traders, processors, civil society, researchers, and government policy-makers – to discuss and agree on how to move towards more sustainable management of commercial medicinal plants in Nepal, including development of a roadmap. Based on the existing knowledge of the sector (Smith-Hall et al., 2020) six groups of participants were identified: (i) harvesters/producers, (ii) traders and wholesalers, (iii) processors, (iv) government, (v) civil society, and (vi) universities and research. For each group, a list of possible participants were made with point of departure in the same reference, emphasizing that all workshop participants should have experience with one or more aspects of the medicinal plant trade. The gross list of possible participants were contacted; those who could participate on the specified date were invited.

Building on a systematic review of proposed interventions in the literature, including an annotated bibliography of all literature and annual dialogue meetings with key stakeholders in 2016, 2017, and 2018, we identified five foundation stones upon which to build future interventions (e.g. a long tradition of successful community management of natural resources) and grouped existing interventions into five explicitly described pathways to increase sustainability: (i) increase cultivation, (ii) strengthen local management, (iii) support domestic industries, (iv) improve sector governance, and (v) establish regional collaboration.

# Prearrangements

## Workshop arrangements

Several online meetings were held with the organisers (ANSAB, TRAFFIC International, and the University of Copenhagen) to determine the modality of the workshop. The pre-workshop online meetings mainly focused on structuring the workshop to facilitate maximum inputs from the participants.

## Participants

A total of 40 participants attended the workshop, 29 were external, and 11 from the organising team (ANSAB, TRAFFIC International, and the University of Copenhagen). External participants represented: (i) harvesters/producers (e.g. FECOFUN); (ii) traders and wholesalers (e.g. JABAN); (iii) processors (e.g. NEHHPA); (iv) government (e.g. Department of Plant Resources, Department of Forests and Soil Conservation, Department of National Parks and Wildlife Conservation, Forest Research and Training Centre); (v) civil society; and (vi) universities and research (e.g. IOF, KAFCOL, RECAST). See Annex 2 for the list of participants.

The workshop mainly focused on exercises conducted in four groups, with each group having a mix of stakeholders, e.g., policy-makers/regulatory officials, academia/research institutions, harvesters, traders and exporters, processors, and civil societies.

## Training the rapporteurs

For the smooth operation of the workshop, rapporteurs were recruited. A half-day pre-workshop training with the rapporteurs was held on 17th August at ANSAB. The training first informed rapporteurs about the objective and purpose of the workshop. Then, each of the four exercises was described in detail. Rapporteurs were also asked to make notes on any additions or removal of actions or pathways. Afterwards, a mock exercise was conducted with the rapporteurs, which helped them to learn more about the practical aspects of the exercise.

# The workshop

The workshop was held on 24^th^ August 2023 at Hotel Himalaya, Lalitpur. The presentation slides were in English, whereas all the group work was in Nepali. All groups did the same exercises. Written group findings and plenum discussions were in English. The rapporteurs facilitated and wrote down findings and decisions. Participants were divided into four groups (Jatamansi, Kutki, Tejpat, and Timur).

## Exercise 1: Validating the pathways and addition/removal of actions

The organising team identified five pathways from a rigorous literature review, each with three to six actions. The purpose of the first exercise was to check the actions for each pathway. For each pathway, participants discussed whether the list of actions was complete or not? Should actions be added or deleted? Because of time constraints (15 minutes for a pathway), each group exercised four out of five pathways, as given in Table 1. Each group started with different pathways; e.g., the Jatamansi group started with Pathway 1, Kutki started with Pathway 2 and so on (Table 1).

Table 1: Groups and pathways

| Group | Pathway I | Pathway II | Pathway III | Pathway IV | Pathway V |
| --- | --- | --- | --- | --- | --- |
| Jatamansi | **X** | X | X | X |  |
| Kutki |  | **X** | X | X | X |
| Tejpat | X |  | **X** | X | X |
| Timur | X | X |  | **X** | X |

Table 2 provides an overview of the starting point for the first exercise.

Table 2: Pathways and actions identified by the organising team prior to the workshop

| Pathways | I | II | III | IV |  | | V | |  |
| --- | --- | --- | --- | --- | --- | --- | --- | --- | --- |
|  | Increase cultivation | Strengthen local management | Support domestic industries | Improve sector governance |  | | Establish regional collaboration | |  |
|  | Nepal | | | | |  | | Nepal, India, China | |
| Actions | - 1. Develop cultivation techniques for species subject to rapid price increases and/or constant high demand, including farmer bottom-up trials. Prioritise species in the Middle Hills   2. Establish infrastructure for post-harvest handling, such as drying facilities at the local level   3. Disseminate techniques and materials   4. Make tax exemption process for cultivated products easier | - 1. Hand-over high-altitude areas to local institutions   2. Allow local management and harvesting systems and local experimentation   3. Develop species-level optimal harvesting methods   4. Disseminate methods   5. Support conflict resolution | 1. Facilitate third-party certification to access non-domestic markets 2. Increase ease of doing business 3. Increase access to low-cost technology 4. Establish infrastructural support such as for product quality testing 5. Support establishment of e-commerce 6. Simplify export procedures | - 1. Invest where it matters - harvest intensities for vulnerable species, consumer surveys   2. Develop a decentralised system for monitoring trade, generating credible official trade statistics   3. Establish an economic incentive-based approach to trade and conservation   4. Promote price dissemination and transparency |  | | - 1. Dismantle Indian cross-border trade barriers   2. Coordinate species protection measures   3. Facilitate technology transfer | |  |

Tables 3-6 provides the results of the first exercise per group.

#### **Jatamansi group**

Actions were added in pathways I, II and III. The group did not remove or shift any pathways.

Table 3: Exercise of Jatamansi group. Actions in black were left as such, in purple were rephrased, in green were added, in blue were removed, and in dark orange were the justifications.

| Pathways | I | II | III | IV |  |
| --- | --- | --- | --- | --- | --- |
|  | Increase cultivation | Strengthen local management | Support domestic industries | Improve sector governance |  |
|  | Nepal | | | | |
| Actions | - 1. Develop cultivation techniques for species subject to rapid price increases and/or constant high demand, including farmer bottom-up trials. Prioritise species in the Middle Hills, followed by other suitable geographic regions (participants feel Tarai are equally important for cultivation)   2. Establish infrastructure for post-harvest handling, such as drying facilities at the local level   3. Disseminate post harvesting techniques and materials   4. Make tax exemption process for cultivated products easier   5. Enhance good cultivation/collection practice   6. Conduct market survey to identify species in high demand for cultivation   7. Conduct capacity-building activities for labour/harvesters   8. Enhance conservation (in situ/ex-situ) | - 1. Hand-over high-altitude areas to local institutions (leading to conservation of wild medicinal plants)   2. Allow local management and harvesting systems and local experimentation   3. Develop and disseminate species-level optimal harvesting methods (for wild harvested plants)   4. Support conflict resolution   5. Prioritise areas with high-growth species/ Important Plant Areas (This helps for better management of areas)   6. Increase Capacity building for local harvesters and processing facilities/techniques   7. Introduce insurance policies (Insurance exists for agriculture but not for forest based products. Insurance will help to grow this sector) | 1. Facilitate third-party certification to access non-domestic markets 2. Increase ease of doing business 3. Increase access to low-cost technology 4. Establish infrastructural support such as for product quality testing 5. Support establishment of e-commerce 6. Simplify export procedures 7. Enhance/facilitate domestic market and consumption in-country (Domestic market will open up opportunities for exporting final and ready-to-use MAPs (e.g. capsules, pills, oils, rather than exporting dried bulbs, and roots)) 8. R&D to promote domestic market and consumption (e.g. value chain research) | - 1. Invest where it matters - harvest intensities for vulnerable species, consumer surveys   2. Develop a decentralised system for monitoring trade, generating credible official trade statistics   3. Establish an economic incentive-based approach to trade and conservation   4. Promote price dissemination and transparency |  |

#### **Kutki group**

The group suggested changing the name of the third pathway to "Support domestic business" to is to include domestic trade in raw materials, which constitutes a substantial volume and engages a wide range of production network actors viz.: harvesters, traders, and central wholesalers.

Table 4: Exercise of Kutki group. Actions in black were left as such, in purple were rephrased, in green were added, in blue were removed, and in dark orange were the justifications.

| Pathways | II | III | IV |  | V |
| --- | --- | --- | --- | --- | --- |
|  | Strengthen local management | Support domestic business | Improve sector governance |  | Establish regional collaboration |
|  | Nepal | | |  | Nepal, India, China |
| Actions | - 1. Hand over high altitude areas to the local institutions based on sustainable management practices and effective management (sustainable management and management capacity should be considered while handover)   2. Allow local management and harvesting systems and local experimentation   3. Develop species-level sustainable harvesting methods (optimal harvesting is different than sustainable harvesting)   4. Disseminate methods   5. Support conflict resolution   6. Restore and rehabilitate the degraded forest areas (restoration and rehabilitation of the degraded natural habitats is crucial for local management) | 1. Facilitate third-party certification to access non-domestic markets 2. Create an enabling environment for doing business - Tax exemption, Tax procedure, reform physical measures (the enabling environment needs to be defined clearly) 3. Support access to improved technology (pre and post-harvest) with equipment and knowledge (needs to focus on access to improved technologies) 4. Enhance infrastructural support such as for product quality testing 5. Support establishment of e-commerce 6. Simplify export procedures | - 1. Invest where it matters - harvest intensities for vulnerable species, consumer surveys (who invests is an issue, thus better to keep in parking slot)   2. Develop a decentralised system for monitoring trade, generating credible official trade statistics   3. Establish an economic incentive-based approach to trade and conservation (not a governance action)   4. Promote price dissemination and transparency   5. Advocacy on policy reform (strengthening the policy is must for this pathway)   6. Establish conflict and grievance resolution measures |  | - 1. Establish a mechanism for cross-border trade issues between Nepal and India or formation of a loose forum (Establishing is better than dismantling)   2. Coordinate species protection measures   3. Facilitate technology transfer   4. Create an enabling environment for FDI and Technology (equipment and knowledge)   5. Forward and Backward linkages   6. Involve embassies of Nepal in market extension   7. Removing Technical Barriers to Trade (For establishing the regional collaboration the bottlenecks like TBT should be removed or minimized and create an enabling environment for access to regional and international market) |

#### **Tejpat group**

Actions were added to four pathways.

Table 5: Exercise of Tejpat group. Actions in black were left as such, in purple were rephrased, in green were added, in blue were removed, and in dark orange were the justifications.

| Pathways | I | III | IV |  | V |
| --- | --- | --- | --- | --- | --- |
|  | Nepal | | |  | Nepal, India, China |
|  | Increase cultivation | Support domestic industries | Improve sector governance |  | Establish regional collaboration |
| Actions | - 1. Develop cultivation techniques for species subject to rapid price increases and/or constant high demand, including farmer bottom-up trials. Prioritise species in the Middle Hills   2. Establish infrastructure for post-harvest handling, such as drying facilities at the local level   3. Disseminate techniques and materials   4. Make tax exemption process for cultivated products easier   5. Establish high tech nursery for quality plant material (R&D integrated in pathway)   6. Conduct market research for high-value cultivated plant products (R&D integrated in pathway)   7. Target government financial support for cultivation (e.g., Insurance, Grants, Subsidies)   8. Conduct Research and Development for promotion of species-wise cultivation and GACP manual | 1. Facilitate third-party certification to access non-domestic markets 2. Increase ease of doing business 3. Increase access to low-cost indigenous technology 4. Establish infrastructural support such as for product quality testing 5. Support establishment of e-commerce 6. Simplify export procedures 7. Promote product diversification and value addition 8. Target government financial support for industrial development and market 9. Conduct Market access, Link and Branding 10. Lobby to invest at least 1% in R&D | - 1. Invest where it matters - harvest intensities for vulnerable species, consumer surveys   2. Develop a decentralised system for monitoring trade, generating credible official trade statistics   3. Establish an economic incentive-based approach to trade and conservation   4. Promote price dissemination and transparency   5. Train/Recruit expert Human Resources to identify the species.   6. Good governance for monitoring.   7. Promote Contract Farming and Buy Back Guarantee. Public Government Monitoring through MIS, online, mobile Apps, |  | - 1. Dismantle Indian cross-border trade barriers   2. Coordinate species protection measures   3. Facilitate technology transfer   4. Review Policy for Importing Countries (This is still lacking where we do not consider the policies of importing countries)   5. Create an environment for FDI (Hard to expand without the FDI)   6. Review global Policy on MAPs trade and conservation and share among all stakeholders for awareness.   7. Invest in research and development, especially in laboratory facilities |

#### **Timur group**

The group proposed changing the fifth pathway's title "Establish regional collaboration" to "Increased international collaboration: India, China and beyond". Though India and China are the major markets for Nepal’s medicinal plants, more than 50 countries are now importing medicinal plants from Nepal, potential growth markets.

Given below in Table 6 is the exercise 1 conducted by Timur group. Actions were rephrased or added in pathways 1, II, IV, and V.

Table 6: Exercise of Timur group. Actions in black were left as such, in purple were rephrased, in green were added, in blue were removed, and in dark orange were the justifications.

| Pathways | I | II | IV |  | | V | |  |
| --- | --- | --- | --- | --- | --- | --- | --- | --- |
|  | Increase cultivation | Strengthen local management | Improve sector governance |  | | Increased International collaboration | |  |
|  | Nepal | | | |  | | India, China and beyond | |
| Actions | - 1. Develop cultivation techniques for species subject to rapid price increases and/or constant high demand, including farmer bottom-up trials. Prioritise species in the Middle Hills   2. Establish infrastructure for post-harvest handling, such as drying facilities at the local level   3. Disseminate techniques and materials   4. Make tax exemption process for cultivated products easier   5. Promote species with a high content of compounds   6. Research on agro-technology for cultivation and efficacy   7. Enhance conservation and cultivation   8. Domestication of cultivation practices   9. Promote tissue culture of high demanded species (This will give us liberty to cultivate the high yielding varieties) | - 1. Developing management plans and policies of high altitude pasturelands and rangelands   2. Allow local management and harvesting systems and local experimentation   3. Develop species-level optimal harvesting methods   4. Disseminate methods   5. Support conflict resolution   6. Develop a mechanism for quality control or value addition   7. Resource inventory (We do not have authentic data on availability of wild medicinal plants)   8. Implement of sustainable harvesting practices   9. Support renewal and revision of CFUG management plans (Resources cannot be harvested with outdated management plan; many CFUGs have inadequate fund for renewal) | - 1. Invest in educating concerned stakeholders on government rules, regulations and policies   2. Develop a decentralised system for social and ecological monitoring for MAPs value chain   3. Establish an economic incentive-based approach to trade and conservation   4. Promote price dissemination and transparency |  | | - 1. Dismantle Indian cross-border trade barriers   2. Coordinate species protection measures   3. Facilitate technology transfer   4. Establish a common regulatory framework for the commercialisation of MAPs (for countries with similar settings)   5. Government readiness to PRA – Pest Risk Assessment (It will be difficult to export medicinal plants without PRA report in near future)   6. Update and harmonise MAPs in China list (We have lists for India but not for China; and China is one of the major importer of Nepali medicinal plants)   7. Develop a platform to regulate CITES issues | |  |

## Exercise 2: Missing pathways?

The commercial medicinal plant production network is dynamic; new opportunities and constraints continually emerge or disappear. Therefore, there may be pathways towards sustainable management that have not been identified in the literature. Therefore, this exercise aimed to identify and justify any missing pathways. The exercise sought answers to the following questions: (i) What is relevant for sustainable management of commercial medicinal plants in Nepal? (ii) If there is a new pathway, what is the pathway's name, and (iii) what are the associated actions? A total of 30 minutes was allocated for this exercise.

#### **Jatamansi group**

This group added one new pathway "Research and Development" and three more actions. Group members found that without proper research, the medicinal plants sector in Nepal will not be able to grow as it should. Participants also felt that inadequate research is why we cannot reap the benefits from the medicinal plants sector.

| Pathways | VI |
| --- | --- |
|  | Research and Development |
|  | [countries relevant to the pathway] |
| Actions | - 1. Promote species with high alkaloid content   2. Develop quality control mechanisms (e.g., accreditation)   3. Conduct intensive research on the domestic market |

#### **Kutki group**

The Kutki group first identified "Research and Development" as a new pathway but later agreed not to add any pathways, instead integrating the R&D-related actions into other appropriate pathways. The group suggested changing the title of the third pathway "Support domestic industries" to "Support domestic business'. The actions identified for R&D were incorporated into other relevant pathways.

#### **Tejpat group**

This group added "Conservation of medicinal plants" as a new pathway and three actions for this pathway.

| Pathways | VI |
| --- | --- |
|  | Conservation of medicinal plants |
|  | Nepal |
| Actions | - 1. Conservation education for wise and sustainable utilisation   2. Develop promotion and utilisation of medicinal plants   3. Ensure the sustainable harvest of medicinal plants |

#### **Timur group**

The group added "Research and Development" and "Promote Sustainable Management of Medicinal Plants" and associated actions.

| Pathways | VI |
| --- | --- |
|  | Research and Development |
|  | Nepal |
| Actions | - 1. Develop a mechanism to retain the efficacy of medicinal plants - especially for domesticated and cultivated plants   2. Enhance collaboration among research institutions - GoN, academia, NGOs, private sectors   3. Strengthen and develop policy formulation based on action research   4. Laboratory analysis and testing services   5. Promote tissue culture of highly demanded species for mass cultivation   6. Policy and market research |

| Pathways | VII |
| --- | --- |
|  | Promote Sustainable Management of medicinal plants |
|  | Nepal |
| Actions | 7.1 Conduct resource inventory of highly traded (volume and cumulative value) medicinal plants  7.2 Upgrade the list of medicinal plants in forest regulation (addition or removal)  7.3 Promote and implement sustainable harvesting practices  7.4 Support renewal and revision of CFUG management plan  7.5 Strengthen the monitoring and evaluation (for wild harvested species) |

## Exercise 3: Ranking pathways

The pathways build a shared understanding of the theory of change, connecting actions to the overall objective, i.e. establishing sustainable management of commercial medicinal plants in Nepal. However, resources are limited, and not all options can be pursued. Choices are necessary. Hence, it is useful to prioritise pathways. As we strive for consensus across different types of actors, the prioritisation is done in the pre-formed multi-actor groups. The time for this exercise was half an hour.

The participants added three more pathways: "Research and Development" were added by the Jatamansi and Timur groups, the "Conservation" pathway by the Tejpat group, and "Promote Sustainable Management of medicinal plants" by the Timur group.

The new proposed pathways were discussed among all the workshop participants. The participants unanimously agreed to add "Research and Development" actions in the respective existing five pathways. After a series of discussions (within each group, among the groups and with all participants), it was agreed that all existing five pathways and associated actions lead to sustainability and conservation and, hence, that the proposed additional pathways “Conservation of medicinal plants” and “Promote Sustainable Management of medicinal plants” could be integrated into the original five pathways. Thus, no new pathways were added. This led to the below pairwise-ranking of five pathways, with the results per group.

#### **Jatamansi group**

|  | **Pathway** | | | | | **Score** |
| --- | --- | --- | --- | --- | --- | --- |
| **Pathway** | **I** | **II** | **III** | **IV** | **V** |  |
| I. Increase cultivation |  | II | I | IV | I | 2 |
| II. Strengthen local management | II |  | II | II | II | 4 |
| III. Support domestic business | I | II |  | 1V | III | 1 |
| IV. Improve sector governance | IV | II | IV |  | IV | 3 |
| V. Increased international collaboration | I | II | III | IV |  | 0 |

#### **Kutki group**

|  | **Pathway** | | | | | **Score** |
| --- | --- | --- | --- | --- | --- | --- |
| **Pathway** | **I** | **II** | **III** | **IV** | **V** |  |
| I. Increase cultivation |  | II | 1 | 1 | 1 | 3 |
| II. Strengthen local management | II |  | II | II | II | 4 |
| III. Support domestic business | I | II |  | III | III | 2 |
| IV. Improve sector governance | I | II | III |  | IV | 1 |
| V. Increased international collaboration | I | II | III | IV |  | 0 |

#### **Tejpat group**

|  | **Pathway** | | | | | **Score** |
| --- | --- | --- | --- | --- | --- | --- |
| **Pathway** | **I** | **II** | **III** | **IV** | **V** |  |
| I. Increase cultivation |  | I | III | I | I | 3 |
| II. Strengthen local management | I |  | II | IV | IV | 1 |
| III. Support domestic business | III | II |  | IV | V | 1 |
| IV. Improve sector governance | I | IV | IV |  | IV | 3 |
| V. Increased international collaboration | I | IV | V | IV |  | 1 |

#### **Timur group**

|  | **Pathway** | | | | | **Score** |
| --- | --- | --- | --- | --- | --- | --- |
| **Pathway** | **I** | **II** | **III** | **IV** | **V** |  |
| I. Increase cultivation |  | I | III | IV | V | 1 |
| II. Strengthen local management | I |  | II | IV | II | 2 |
| III. Support domestic business | III | II |  | IV | V | 1 |
| IV. Improve sector governance | IV | IV | IV |  | IV | 4 |
| V. Increased international collaboration | V | II | V | IV |  | 2 |

The results were aggregated in the table below. Aggregating the scores from all four groups disclosed that improving sector governance is ranked first jointly with strengthen local management, followed by increase cultivation. This table was then discussed as the basis for Exercise 4.

| Pathway | Jatamansi | Kutki | Tejpat | Timur | Total score | Final rank |
| --- | --- | --- | --- | --- | --- | --- |
| 1. Increase cultivation | 2 | 3 | 3 | 1 | 9 | Second |
| 2. Strengthen local management | 4 | 4 | 1 | 2 | 11 | First |
| 3. Support domestic business | 1 | 2 | 1 | 1 | 5 | Third |
| 4. Improve sector governance | 3 | 1 | 3 | 4 | 11 | First |
| 5. Increased international collaboration | 0 | 0 | 1 | 2 | 3 | Fourth |

## Exercise 4: Ranking actions in the top three prioritised pathways

The pathways consist of a number of discrete actions, some of which are more feasible, e.g. due to costs. Ranking the actions in a pathway will help identify the priority actions. Again, as we strive for consensus across different types of actors, the prioritisation was done in the pre-formed multi-actor groups.

Initially, the organisers planned to pairwise-rank the actions for the prioritised pathways. However, due to the increased number of actions in each pathway, participants were instead asked to absolute rank at least the top three actions in each of the five pathways.

Going back to the results of the first exercise, the four groups independently scrutinised the actions. A few of the proposed actions were rephrased, reworded, or grouped together due to their similarity. Table 7 presents the five pathways and the integrated actions resulting from the work of the four groups.

Participants were then asked to prioritise/rank at least three actions in each pathway. The list of prioritised actions for each pathway was presented, discussed, and agreed in plenum, Table 8.

# Closing remarks

The closing remarks on the workshop were given by representatives of different groups: harvesters, traders, exporters, processors, and government institutions. All participants found the workshop useful. The participants appreciated the structure of the workshop and expressed recommendation that the workshop's outcomes (pathways and actions) should be used as a platform to guide the development of the medicinal plants sector in Nepal.

Table 7: Integrated set of actions for each of the five pathways

| Pathways | I | II | III | IV |  | V |
| --- | --- | --- | --- | --- | --- | --- |
|  | Increase cultivation | Strengthen local management | Support domestic business | Improve sector governance |  | Increased international collaboration |
|  | Nepal | | | |  | Nepal, India, China |
| Actions | - 1. Develop cultivation techniques for species subject to rapid price increases and/or constant high demand, including farmer bottom-up trials. Prioritise species in the Middle Hills and then expand to all geographic regions   2. Establish infrastructure for post-harvest handling, such as drying facilities at sub-local (village) and local (district) level   3. Develop and disseminate post-harvesting techniques and materials   4. Make tax exemption process for cultivated products easier   5. Establish high-tech nurseries for the production of quality plant material   6. Conduct market research and survey (demand and supply) for high-value cultivated plant products   7. Optimised use of government support for cultivation (Grants, Subsidies)   8. Introduce insurance policies for forest products, especially NTFPs and medicinal plants   9. Conduct Research and Development for promotion of species-wise cultivation of valuable plant species (e.g., with high alkaloid content)   10. Develop and enhance species-wise GACP for highly traded products   11. Conduct capacity-building activities for labour/harvesters to ensure the quality of raw materials   12. Research on agro-technology for cultivation and efficacy   13. Enhance *in situ* and *ex-situ* conservation of medicinal plants   14. Promote tissue culture of high demanded species | - 1. Hand over high altitude areas to the local institutions based on sustainable management practices and effective management or Develop management plans and policies for high altitude pasturelands and rangelands   2. Allow local management and harvesting systems and local experimentation   3. Support conflict resolution, especially during wild harvesting   4. Prioritise areas with high-growth species /Important Plant Areas   5. Increase capacity building for local harvesters and processing facilities/techniques   6. Develop mechanisms for quality control and value addition   7. Conduct resource inventory of major medicinal plant species   8. Develop species-level optimal harvesting methods and implement sustainable harvesting practices   9. Support renewal and revision of CFUG management plan   10. Preparation of a sustainable harvesting plan and implementation of sustainable harvesting practices   11. Local experimentation practices (regeneration management, rotational harvesting and restoration)   12. Educate harvesters and traders on conservation and sustainable management of medicinal plants   13. Strengthen monitoring for wild harvested species | 1. Maintain a nationwide record of domestic trade 2. Facilitate third-party certification to access non-domestic markets 3. Create an enabling environment for doing business-Tax exemption, Tax procedure, reform physical measures 4. Support access to improved indigenous technology (pre and post-harvest) with equipment and knowledge 5. Enhance infrastructural support such as for product quality testing 6. Support establishment of e-commerce 7. Simplify export procedures for raw medicinal plants 8. Promote product diversification and value addition 9. Target government financial support for Industrial development and market 10. Conduct Market Access, Link and Branding 11. Allocate 1% of income for R&D 12. Enhance/facilitate domestic market and consumption in-country 13. R&D to promote domestic market and consumption (e.g. value chain research) 14. Laboratory analysis and testing services for products; Product development and diversification 15. Access to improved technology (post-harvest) | - 1. Invest in educating concerned stakeholders on government rules, regulations and policies; also harvest intensities for vulnerable species   2. Develop a decentralised system for social and ecological monitoring of medicinal plants value chain   3. Train/Recruit expert Human Resources to identify the species. Good Governance for monitoring. Promote Contract Farming and Buy Back Guarantee. Public Government Monitoring through MIS, online, mobile Apps,   4. Establish an economic incentive-based approach to trade and conservation   5. Promote price dissemination and transparency   6. Develop medicinal plants Curriculum at the local level   7. Strengthen the government's official sectoral database   8. Strengthen government institutions and infrastructure for efficient sectoral governance   9. Updating the list of MAPs in Forest Regulations   10. Strengthen monitoring and evaluation   11. Implementation of law re-enforcement   12. Advocacy on policy reform |  | - 1. Establish a mechanism for cross-border trade issues between Nepal and India or formation of a loose forum   2. Coordinate species protection measures   3. Facilitate technology transfer   4. Review Policy for Importing Countries   5. Review global Policy on MAPs trade and conservation and share among all stakeholders for awareness.   6. Establish a common regulatory framework for the commercialisation of medicinal plants (for countries with similar settings)   7. Government readiness to PRA – Pest Risk Assessment   8. Update and harmonise MAPs in China list   9. Develop a platform to regulate CITES issues   10. Create an enabling environment for FDI and Technology (equipment and knowledge)   11. Involve embassies of Nepal in market extension   12. Removing or minimizing Technical Barriers to Trade   13. Enhance collaboration among research institutions, academia, GoN, private sectors, NGOs |

Table 8: Final prioritised set of actions for each of the five pathways

| **Pathways** | **I** | **II** | **III** | **IV** | **V** |
| --- | --- | --- | --- | --- | --- |
|  | **Increase cultivation** | **Strengthen local management** | **Support domestic business** | **Improve sector governance** | **Increased international collaboration** |
|  | Nepal | | | | India, China, and beyond |
| Actions | - 1. Conduct market research and survey (demand and supply) for high-value cultivated plant products   2. Optimised use of government support for cultivation (Grants, Subsidies)   3. Conduct Research and Development for promotion of species-wise cultivation of valuable plant species (e.g., species with high alkaloid content)   4. Develop and enhance species-wise GACP for highly traded products   5. Research on agro-technology for cultivation and efficacy | - 1. Hand over high altitude areas to the local institutions based on sustainable management practices and effective management or Develop management plans and policies for high altitude pasturelands and rangelands   2. Support conflict resolution, especially during wild harvesting   3. Increase Capacity building for local harvesters and processing facilities/techniques   4. Support renewal and revision of CFUG management plan   5. Preparation of a sustainable harvesting plan and implementation of sustainable harvesting practices | 1. Create an enabling environment for doing business-Tax exemption, Tax procedure, reform physical measures 2. Support access to improved indigenous technology (pre and post-harvest) with equipment and knowledge 3. Enhance infrastructural support such as for product quality testing 4. Promote product diversification and value addition 5. Target government financial support for Industrial development and market 6. Allocate 1% of income for R&D 7. Enhance/facilitate domestic market and consumption in-country 8. Laboratory analysis and testing services for products; Product development and diversification | - 1. Invest in educating concerned stakeholders on government rules, regulations and policies; also harvest intensities for vulnerable species   2. Develop a decentralised system for social and ecological monitoring of medicinal plants value chain   3. Train/Recruit expert Human Resources to identify the species. Good Governance for monitoring. Promote Contract Farming and Buy Back Guarantee. Public Government Monitoring through MIS, online, mobile Apps,   4. Establish an economic incentive-based approach to trade and conservation   5. Promote price dissemination and transparency   6. Develop medicinal plants Curriculum at the local level   7. Strengthen government institutions and infrastructure for efficient sectoral governance | - 1. Establish a mechanism for cross-border trade issues between Nepal and India or formation of a loose forum   2. Coordinate species protection measures   3. Review Policy for Importing Countries   4. Review global Policy on MAPs trade and conservation and share among all stakeholders for awareness.   5. Government readiness to PRA – Pest Risk Assessment   6. Create an enabling environment for FDI and Technology (equipment and knowledge) |

# Appendix S7 List of pathway action assumptions for the roadmap to sustainable management of commercial medicinal and aromatic plants, fungi, and lichens in Nepal

The comments and references are focused on the case of sustainable management of commercial medicinal plants in Nepal. They are a starting point for examining the specified assumptions and can be further developed with structured reviews, also systematically integrating experiences from beyond the case.

| ***Assumptions*** | ***Comments and references*** |
| --- | --- |
| *Pathway I: Increase cultivation* | |
| Action 1.1 Develop agro-technology for cultivation, including through government support and establishing Good Agricultural Practices | |
| Technology for native species can be developed, disseminated, and successfully used by small-scale farmers | Existing examples of successful cultivation of previously wild-harvested species (Chaudhary et al., 2013; Karki & Chowdhary, 2019; Pyakurel, 2020; Yadav, 2013) indicate that technical problems can be solved, including bottom-up through farmer trials. A model explaining the dynamics of environmental product cultivation pathways (Madsen and Smith-Hall, 2023) is available. Guidelines on good agricultural and collection practices for medicinal plants are also available (WHO, 2003). Socioeconomically, outmigration leads to a lack of labor and land extensification (Childs et al., 2014; Ojha et al., 2022; World Bank, 2023a), making less labor-intensive non-crop alternatives more attractive. Also, wild harvesting is considered an arduous and low-prestige activity (Larsen & Smith, 2004). Infrastructure development also lowers cultivation costs (Pyakurel et al., 2018). |
| Action 1.2 Conduct market surveys and promotion campaigns for high-value cultivated plant products | |
| Demand will persist or increase for cultivated products.  Consumer surveys can be conducted. | The global medicinal plant export market was valued at around 2.6 billion USD in 2016, tripling over the last 15 years, suggesting a relatively inelastic demand outstripping supply (World Bank, 2018). According to GIZ (2017), the global trade in medicinal plants has increased by 3% annually since 2010. Increasing incomes in the main consumption countries of India and China is likely to increase demand (Goraya & Ved, 2017; Hinsley et al., 2020; Pyakurel et al., 2018).  There are no existing consumer surveys for any of the commercial medicinal plants from Nepal (Smith-Hall et al., 2020). The exception is for *Ophiocordyceps sinensis* in China (He et al., 2022) and *Neopicrorhiza scrophulariiflora* in Nepal (Kafle et al., 2018), showing that such surveys can be successfully conducted. |
| *Pathway II: Strengthen local management* | |
| Action 2.1 Hand-over of high-altitude areas to local institutions | |
| Division Forest Offices and Conservation Area Offices actively engaged in increased handover of medicinal plant production habitats to local communities  Increased local control of medicinal plant resources increases productivity (less: habitat destruction, premature harvesting, overharvesting, harvesting of banned species) and incomes. | Most forest areas in the middle hills have been handed over to local communities through the community forestry mechanism (MoFE, 2021). This indicates that the same can be achieved at scale for high-altitude areas such as alpine meadows. The preconditions exist: high-altitude forest and non-forest areas can be legally handed over to local communities (Pyakurel, 2020).  Community forestry has documented positive conservation outcomes (e.g. Hajjar et al., 2021; Luintel et al., 2018; Paudel et al., 2022). Regarding medicinal plants specifically, emerging experiences document positive conservation outcomes from local engagement in medicinal plant monitoring and management (e.g. Chapagain et al., 2021; Poudeyal et al., 2019). |
| Action 2.2 Support development and renewal of local management plans, including implementation of sustainable harvesting practices | |
| The existing regulations for local community management, including standardized approaches to harvesting and five-year management plans, are used to support local systems and experimentation. | Recent advances in understanding how community forestry is implemented in Nepal (Baral et al., 2018; Basnyat et al., 2018, 2019) document the importance of understanding the political ecology of commercial medicinal plant management and trade in Nepal. This is required to develop feasible implementation of actions, e.g. Baral et al. (2018) showed that inventory requirements in community forestry were used mainly to satisfy bureaucratic requirements rather than improve forest management. |
| Action 2.3 Support local conflict resolution, especially during wild harvesting | |
| Local authorities (Division Forest Offices, Park offices and Conservation Area management council / offices) can engage in mediating resource access conflicts and find solutions acceptable to involved parties | Present guidelines for managing high-value products (such as the Yarsagumba Management Directive 2073) are not focused on conflict resolution. Conflicts can be severe, leading to the killing of harvesters (Adhikari, 2017) and the breakdown of traditional livelihood strategies (Pant et al., 2017), and must be explicitly addressed. This will require targeted investments to develop such skills and make them available locally. |
| Action 2.4 Increase capacity for local-level value-addition | |
| Value-addition options exist that can be locally implemented | There are generally no product-level distinctions between qualities when purchasing from producers (Smith-Hall et al., 2020), even though such qualities exist further down the production network (Choudhary et al., 2013). Also, improved communication (Pyakurel et al., 2018) and establishing e-commerce (He, 2023) hold the potential to increase producers’ net margins. |
| *Pathway III: Support domestic businesses* | |
| Action 3.1 Support access to improved technology | |
| Access to improved technology will increase competitiveness and allow access to wider markets | Traditional medicine systems in India and China have been increasingly commercialized in the last two decades (Harilal, 2009; Kala, 2015; Kloos et al., 2020), including mass production and marketing, indicating the existence of relevant improved technology. Developments in Nepal have so far been dominated by smaller enterprises with low access to technology and producing lower-value consumer products for the domestic market (Caporale et al., 2020). |
| Action 3.2 Enhance infrastructure, incl. laboratories for product quality testing | |
| Improved standard infrastructure will enhance market access | Access to high-end consumer markets, e.g. in the EU, requires documented and stringent quality control procedures (Gurung, 2013). In Nepal, the low quality of storage technologies and lack of supportive infrastructure, including the accredited laboratory testing of product quality, has been identified as a barrier to sector development (Caporale et al., 2020). |
| Action 3.3 Support product diversification and value addition through private sector and government investments | |
| Diversification and value addition allow market expansion | This has happened in India and China in the last two decades (Harilal, 2009; Kloos et al., 2020). So far, in Nepal, the private sector has financed the expansion of medicinal plant secondary processing enterprises responding to opportunities and market signals. This must continue and be supported by public policy measures and investments, e.g. updating the legal framework for e-commerce and establishing international payment gateways (World Bank, 2018). |
| Action 3.4 Create an enabling environment for doing business, e.g. simplified tax procedures | |
| There is political will to make starting and operating private businesses simpler | Nepal ranked 94 out of 190 countries for ease of doing business in 2019, up from 105 in 2014 (World Bank, 2020). This constraint is similarly found in the broader agribusiness sector (World Bank, 2018). There are province-level changes that can be pursued, e.g. in terms of simplifying trade processes. |
| Action 3.5 Develop the domestic market and increase consumption | |
| This will lead to industry expansion (more jobs) and value-addition (more value retained in Nepal, higher public revenues) | The number of jobs and value-additions has increased in the past with the expansion of the industry in Nepal (Caporale et al., 2020). In terms of the future market and consumption, the World Bank (2018) identified two potential avenues: (i) lightly processed personal care products for the conscientious consumer, and (ii) Ayurvedic heavily processed, mass-produced products. |
| *Pathway IV: Improve sector governance* | |
| Action 4.1 Disseminate rules and knowledge, incl. harvesting guidelines for vulnerable species and need for Pest Risk Assessment | |
| There is substantial existing knowledge that can positively impact sector governance if disseminated and used | There is much existing ecological and economic knowledge that can be integrated and up-scaled to increase sustainable management and trade (Smith-Hall et al., 2023). Given the many commercial species (Pyakurel et al., 2019), the use of traditional ecological knowledge appears important to not only rely on costly scientific estimation of sustainable harvest rates. There are also existing recommendations on how to approach Pest Risk Management and institutionalized quality control (Gurung, 2013) and how to approach risks more widely (TRAFFIC, 2023a). |
| Action 4.2 Establish an economic incentive-based approach to trade and conservation | |
| It is politically possible to build medicinal plant management and trade around an economic incentive framework and increased local participation | The experiences from community forestry are positive in terms of building the required framework, leading to increased local management of resources and positive conservation outcomes (e.g. MoFE, 2021). An increased understanding of the political ecology of the commercial medicinal plant sector is required to ensure that feasible solutions to the benefit of species and sector stakeholders are identified and implemented. There is evidence of vested interest in commercial medicinal plant trade aimed at controlling resource flows and extracting rents (e.g. Adhikari, 2017; Craig & Glover, 2009; Dovydaitis, 2017; Fold et al., 2023). |
| Action 4.3 Promote price dissemination and transparency | |
| There is a public agency with the funding and capacity to collect and disseminate valid product-level prices timely | Asia Network for Sustainable Agriculture and Bioresources (an NGO) collected and disseminated medicinal plant prices from Jan 2010 to Jan 2020, showing this is possible (ANSAB, 2023). Possible public institutions include the Department of Forests and Soil Conservation and the Department of Plant Resources. Both trader, central and regional wholesaler prices (Olsen and Bhattarai, 2005), e.g. monthly, should be collected and disseminated along with locations as there is evidence of spatial arbitration (Olsen & Helles, 2009). |
| Action 4.4 Develop a decentralized system to collect credible official statistics along the value chain, incl. training of officials | |
| An appropriate institution can systematically collect high-quality trade data that are timely shared and published at aggregated levels | Public data is notoriously inaccurate, not reflecting trade levels and showing that data collection based on permits does not work (e.g. Smith-Hall et al., 2023). Instead, local-level data should be explicitly collected through an annual trader survey by an institution not affiliated with the permit system, such as an NGO. This would provide more accurate annual trade data at the species level. This could then be expanded to include central wholesalers and processors to estimate annual export at the species level. |
| Action 4.5 Develop medicinal plants curricula | |
| Including pathway-related issues in curricula will long-term improve roadmap implementation | Updating curricula can have positive on-the-ground impacts (Arevalo et al., 2014) and support the engagement of actors in achieving changes (Chamberlain and Smith-Hall, 2024). |
| *Pathway V: Increase international collaboration* | |
| Action 5.1 Establish a dialogue mechanism for solving cross-border trade issues | |
| The Government of Nepal can negotiate the removal of trade barriers in India and China | There are many examples of unilaterally imposed trade restrictions, e.g. required permits for exports from Nepal in Uttar Pradesh (Fold et al., 2023). Arguably, trade stakeholders have a common interest in pursuing their objectives cross-border, providing the Government of Nepal an opportunity to enhance sustainable trade in the region. |
| Action 5.2 Coordinate species protection measures across borders | |
| The Government of Nepal can negotiate and promote regional species-level conservation | There are many examples of unilaterally implemented conservation measures, e.g. the ban on commercial harvest of *Nardostachys jatamansi* in India (Chauhan et al., 2021), that may lead to leakage (e.g. shifting harvesting from India to Nepal). Arguably, conservation stakeholders have a common interest in pursuing their objectives cross-border, providing the Government of Nepal an opportunity to enhance conservation in the region. |
| Action 5.3 Review relevant importing countries’ and international policies and share knowledge | |
| International legislation and legislation in importing countries influence exports from Nepal | An example of specific legislation in importing countries is the negative opinion of trade in *N. jatamansi* by the European Union’s Scientific Review Group on Trade in Wild Fauna and Flora (e.g. EU, 2018) that recommended a zero-export quota and led to a Government of Nepal proposal for district-level quotas (DFSC, undated). An example of general legislation are the phytosanitary requirements in connection to plant imports into the EU (EU, 2016) or the requirements following from the Convention on International Trade in Endangered Species, e.g. CITES (2018). An overview of medicinal plant nominal and functional law in China, as well as international regulations, are provided by TRAFFIC (2023b). There appears to be substantial scope for standardized collection and sharing of requirements and procedures to facilitate trade and encourage the establishment of long-term trade relationships in export markets. |
| Action 5.4 Create an enabling environment for foreign direct investment and technology transfer | |
| Foreign direct investments and technology transfer will increase the competitiveness of the processors in Nepal | Both access to technology and financial resources have been identified as constraints to developing medicinal plant processing in Nepal (Caporale et al., 2020). There has been success in attracting foreign direct investment in the broader healthcare sector from Indian healthcare providers (World Bank, 2018). However, the lack of an Intellectual Property Rights Law and weak protection of such rights inhibits investments – firms are less likely to invest where their rights are not enforced (World Bank, 2018). |

# Appendix S8 List of pathway outcome assumptions for the roadmap to sustainable management of commercial medicinal and aromatic plants, fungi, and lichens in Nepal

The comments and references are focused on the case of sustainable management of commercial medicinal plants in Nepal. They are a starting point for examining the specified assumptions and can be further developed with structured reviews, also systematically integrating experiences from beyond the case.

| ***Assumptions*** | ***Comments and references*** |
| --- | --- |
| *Pathway I: Increase cultivation* | |
| Outcome 1.7 Less pressure on wild populations | |
| Cultivation will decrease the pressure on wild-harvested plant populations | From a conservation point of view, the development of cultivation techniques should focus on species where increasing prices and resource scarcity do not induce cultivation, such as *N. jatamansi* and *Paris polyphylla* (Madsen and Smith-Hall, 2023). For such species, attention should be paid to contextual (e.g. infrastructure), mediating (e.g. cultivation technology), and harvester (e.g. opportunity cost of labor) factors (Madsen and Smith-Hall, 2023) to facilitate positive cultivation outcomes.  In economic terms, cultivation should lead to less wild harvesting; there is evidence that cultivation tends to be accompanied by lower prices (Madsen & Smith-Hall, 2023). While claims of the conservation benefit of cultivation are widespread in the literature, there is a dearth of studies providing empirical evidence, particularly for plants. The available studies indicate that species maintained in cultivation only through wild-collected seeds or artificial propagation were likely to reduce wild harvesting. However, attention needs to be paid to plant laundering, the passing of cultivated plants as wild-harvested (Liu et al., 2019). |
| Outcome 1.8 Stable supply of higher quantities of high-quality materials | |
| Cultivation allows regular harvests of high-quality materials in high quantities | There may be consumer preferences for wild-harvested products that could lead to lower prices for cultivated products if such distinctions are made in the market (e.g. Li et al., 2019; Liu et al., 2014) though consumer behavior is likely to be complex, e.g. as some consumers will take issues like legality and sustainability into consideration (Hinsley and Sas-Rolfes, 2020). The introduction of cultivation also tends to reduce prices (Madsen and Smith-Hall, 2023). For some species, there may be challenges in terms of producing secondary metabolites during cultivation, but cultivation has also been shown to enable improved purity and quality, consistency and bioactivity (Mofokeng et al., 2022). |
| Outcome 1.9 Higher producer income | |
| Cultivation leads to higher producer income | Even if cultivation tends to reduce per-unit prices (Madsen and Smith-Hall, 2023), the quantity produced per unit area could increase producer income. Cultivation is subject to the usual market price fluctuations. There are examples of fluctuations making cultivation unprofitable, e.g. in the case of *Swertia chirayita* (Cunningham et al., 2018), emphasizing the importance of value-addition or flexibility to shift between medicinal plant crops. Evidence shows that medicinal plant cultivation tends to be done by more well-off households (Madsen and Smith-Hall, 2023), which could increase local income inequality. |
| *Pathway II: Strengthen local management* | |
| Outcome 2.10 Less premature harvesting and less overharvesting in the wild | |
| Increased local responsibility and rights lead to improved conservation outcomes | Positive conservation outcomes in community forestry in Nepal are well-documented (e.g. Luintel et al., 2018; Paudel et al., 2022), even to the extent of widespread under-harvesting (Meilby et al., 2014). This is also generally supported by work on decentralized natural resource governance (Ostrom, 2009). |
| Outcome 2.11 Better resource management, monitoring, and control | |
| Increased local responsibility and rights lead to improved management, monitoring, and control | See comments to 2.10. |
| Outcome 2.12 Improved local-level conflict resolution | |
| Giving local communities higher influence on the management of their resources will lead to better conflict resolution | There appear to be two types of conflicts in relation to wild-harvesting: (i) between interdependent communities, and (ii) with outsiders unrelated to the local community. An example of the former is denying access to *O. sinensis* production areas to would-be harvesters from communities controlling access to winter grazing for upland communities that are then, in return, denied access, resulting in lose-lose outcomes (e.g. Pant et al., 2017). An example of the latter is the denial of access to harvesters from non-neighboring communities, conflicts that can be severe and lead to deaths (Adhikari, 2017). Presumably, increased local management would incentivize solving the first type of conflict. The second type might need a fee system (at the local or national level). |
| Outcome 2.13 Long-term protection of harvester incomes | |
| Improved resource management will maintain or increase future harvester incomes | The positive conservation outcomes in community forestry in Nepal (e.g. Luintel et al., 2018; Paudel et al., 2022) indicate the ability of local management systems to maintain or increase resources. However, realizing harvester incomes from commercial medicinal plants would require eliminating income generation constraints in community forestry (Meilby et al., 2014; Paudel et al., 2022). |
| Outcome 2.14 Higher provincial-level income | |
| Improved resource management will lead to more trade that the local government can tax | The constitution allows provincial-level taxation of commercial medicinal plant products, a right already exercised, e.g. through local taxes on medicinal plant trade (Pyakurel & Smith-Hall, 2023). |
| *Pathway III: Support domestic businesses* | |
| Outcome 3.11 Replacing raw material export with domestic processing | |
| Improved technology, infrastructure, value-addition, and business environment will increase the amount of domestic processing | The domestic processing industry has expanded from less than a score of processors to over two hundred in the last decades (Caporale et al., 2020). The same pull factors, such as rising income in Nepal, appear set to continue, facilitating industry expansion and additional secondary processing, further reinforced by the proposed actions. |
| Outcome 3.12 More competitive secondary processing industry | |
| Improved technology, infrastructure, value-addition, and business environment will increase the competitiveness of the domestic processing industry | The established success of secondary processing industries competing on the domestic market with imports of Indian Ayurvedic medicine suggests competitiveness in this segment (Caporale et al., 2020; World Bank, 2018). Moving from the dominating commodity business model, controlled by regional wholesalers in India (Olsen & Helles, 2009), would take further advantage of the closeness to the resource base. |
| Outcome 3.13 Job creation, higher public revenues | |
| An expanding industry will create new jobs and increase public revenues | The industry is dominated by small enterprises with an average of 6.4±9.8 permanent employees (Caporale et al., 2020), while more than 300,000 households are involved in production (Olsen, 2005). Nepal has arguably achieved an upgrading trajectory from commodities to limited manufacturing from 1990 to 2015 (World Bank, 2020), supporting the possibility of job creation in the medicinal plant industry and achieving middle-income status (World Bank, 2017). The ability to realize higher public revenues would gain from shifting taxation away from the border and improving the implementation of fiscal federalism (World Bank, 2023b). |
| *Pathway IV: Improve sector governance* | |
| Outcome 4.11 More sustainable wild harvesting | |
| Making knowledge available and providing incentives for conservation and trade will increase sustainable wild harvesting | Medicinal plant harvesters act on knowledge, such as new cultivation technologies (e.g. Choudhary et al., 2013) and prices (e.g. Pyakurel et al., 2018). Combined with positive experiences from decentralized forest management in Nepal (e.g. Luintel et al., 2018), it indicates the possibility of implementing sustainable wild harvesting guidelines (e.g. Smith-Hall et al., 2023) for increased sustainable wild harvesting. |
| Outcome 4.12 Fewer options for rent-seeking | |
| An incentive-based approach will diminish the opportunities for rent-seeking | Rent-seeking occurs in connection to issuing permits and checking these as products move through the production network (e.g. Adhikari, 2017; Olsen & Helles, 2009). Replacing these options with incentive-based local resource management, and avoiding the associated rent-seeking traps found in community forestry (e.g. Baral et al., 2018; Basnyat et al., 2019), would diminish rent-seeking. |
| Outcome 4.13 Higher harvester net margins | |
| Less rent-seeking and improved price transparency will increase harvester net margins | The composition of costs and net margins are known (e.g. Fold et al., 2023; Olsen & Helles, 2009). Increased price transparency, e.g., obtained by harvesters using mobile phones, increases harvester bargaining power and hence net incomes (Pyakurel et al., 2018). |
| Outcome 4.14 More transparent production network | |
| More transparent prices and improved statistics will increase production network transparency | Knowledge of Indian demand and prices led to a passive oligopsony of central wholesalers in Nepal (Olsen, 1998) that was challenged by traders increasing their bargaining power due to increased access to infrastructure (phones, roads) and proximity to harvesters (Pyakurel et al., 2018). Open prices and data on volume would reinforce a more competitive market. |
| Outcome 4.15 Higher qualified non-trade stakeholders | |
| Training and education will increase non-trade actor qualifications | There is ample applied science that can be integrated into university programs, such as toolkits for sustainable harvesting of low-altitude tree products (e.g. ANSAB, 2022; Lamichhane & Karna, 2010) or high-altitude perennials (e.g. Larsen, 2005). To achieve positive outcomes from training requires careful selection, design, and follow-up (ADBI, 2007; Subedi, 2008). |
| *Pathway V: Increase international collaboration* | |
| Outcome 5.9 Better international species protection | |
| Cross-border collaboration is possible and will have positive biodiversity conservation outcomes | There are existing cross-border conservation initiatives, such as the Kathmandu-based South Asia Wildlife Enforcement Network (SAWEN, 2022) and the India-Nepal collaboration on tiger conservation (IUCN, 2023). These demonstrate experience with cross-border biodiversity conservation, and SAWEN provides a formalized platform for further discussions and initiatives. China is not a SAWEN member and would require a separate dialogue initiative. |
| Outcome 5.10 Increased market integration | |
| As the main market for medicinal plants is regional and economically important, stakeholders are willing to find and implement solutions that increase market integration | There is ample evidence of the regional nature of the medicinal plant market, with Nepal supplying raw materials to India and China (e.g. Olsen, 2005; Pyakurel et al., 2018). However, individual actors such as the State Government of Uttar Pradesh are pursuing interventions (Fold et al., 2023) not benefiting the medicinal plant industries in India (Goraya & Ved, 2017). SAWEN is focused on curbing illegal trade (SAWEN, 2022) and has no mandate to dismantle legal trade barriers, indicating that another institutional solution must be selected. |
| Outcome 5.11 More competitive export-oriented secondary processing industry | |
| Dismantling trade barriers, more knowledge of policies impacting exports, and more investment and technology transfer will make the processing industry more competitive | These factors have been identified as constraints to developing Nepal's medicinal plant secondary processing industry (Caporale et al., 2020). This approach is also part of wider initiatives to promote economic growth in the country (World Bank, 2017). |

# Appendix S9 Positive feedback loops (reinforcing mechanisms) between roadmap pathways

Below are examples of positive feedback loops between roadmap pathways. The emphasis is on identifying the main mechanisms rather than providing an exhaustive overview. The feedback mechanisms identify bundles of mutually reinforcing actions – packages of actions are likely to be more successful than individual actions.

Note that implementing only selected actions in pathways can lead to negative feedback loops. For instance, if actions 3.1-3.5 in Pathway III Supporting domestic businesses is done without introducing and disseminating cultivation techniques (action 1.1) and increasing the hand-over of local resources (2.1) and supporting their management (2.2, 2.3) this may lead to increased wild-harvesting and less sustainable trade. Also note that the actions in Pathway IV Improve sector governance can be implemented without negative feedback loops, except for 4.2 (Establish an economic incentive-based approach to trade and conservation) that could have negative conservation outcomes if not complemented with resource base actions.

| ***Feedback loops***^1^ | ***Description*** |
| --- | --- |
| 3.1-3.4 and 1.1 | Supporting the development of more competitive medicinal plant processing industries (Pathway III) will increase the demand for more stable and predictable supplies, and hence the incentives to develop cultivation techniques and increase cultivation (Pathway I) |
| 3.1-3.4 and 4.2-4.3 and 2.1-2.4 | Supporting the development of more competitive medicinal plant processing industries (pathway III) and increasing harvester net margins (Pathway IV) will increase the harvesting of vulnerable species unless mediated by incentives to ensure longer-term household incomes (Pathway II) |
| 4.2-4.3 and 5.1 and 2.1-2.4 | Increased market efficiency – through more transparent pricing and an official economic incentive-based approach to medicinal plant management (Pathway IV) and dismantling of cross-border trade barriers (Pathway V) – will increase local community commitment to sustained medicinal plant resource management (Pathway II) |
| 2.1-2.3 and 1.1 and 5.1 | Increased local management of medicinal plant resources and better conflict resolution (Pathway II), increased cultivation (Pathway I), and fewer trader barriers (Pathway V) will increase provincial income |
| 1.2 and 3.3 and 3.5 | Conducting market surveys for high-value cultivated products (Pathway I) will inform the development of product diversification and the domestic market (Pathway III) |
| 1.1 and 2.1-2.3 and 4.2 and 5.2 | More cultivation (Pathway I), more local management of resources (Pathway II), an incentive-based approach to conservation (Pathway IV), and cross-border species protection (Pathway V) will decrease pressure on wild-harvested species of conservation concern |

^1^ Numbering refers to actions in Table 1 in the article *A roadmap to sustainable management of commercial medicinal and aromatic plants, fungi, and lichens in Nepal*.

# References

ADBI. (2007). Workforce development in Nepal. Asian Development Bank Institute, Tokyo. 77pp.

Adhikari, K. (2017). Ethnobotany, commercialization and climate change: consequences of the exploitation of yarsagumba in Nepal. European Bulletin of Himalayan Research, 49, 35-58.

ANSAB. (2022). Toolkit on sustainable harvesting of non-timber forest products (NTFPs). Asia Network for Sustainable Agriculture and Bioresources, Kathmandu. 35pp.

ANSAB. (2023). NTFPs price list. Asia Network for Sustainable Agriculture and Bioresources, Kathmandu, https://ansab.org.np/sub/ntfps-price-list. Accessed 12 Nov 2023.

Arevalo, J., Pitkänen, S., & Kirongo, B. (2014). Developing forestry curricula: experiences from a Kenyan-Finnish project. International Forestry Review, 16 (1), 78–86.

Baral, S., Meilby, H., Chettri, B. B. K., Basnyat, B., Rayamajhi, S., & Awale, S. (2018). Politics of getting the numbers right: Community forest inventory of Nepal. Forest Policy and Economics, 91, 19-26.

Basnyat, B., Treue, T., & Pokharel, R. K. (2019). Bureaucratic recentralisation of Nepal's community forestry sector. International Forestry Review, 21(4), 401-415.

Basnyat, B., Treue, T., Pokharel, R. K., Lamsal, L.N., & Rayamajhi, S. (2018). Legal-sounding bureaucratic re-centralisation of community forestry in Nepal. Forest Policy and Economics, 91, 5-18.

Caporale, F., Mateo-Martín, J., Usman, F., & Smith-Hall, C. (2020). Plant-based sustainable development – the expansion and anatomy of the medicinal plant secondary processing sector in Nepal. Sustainability, 12(14), 5575.

Chamberlain, J., & Smith-Hall, C. (2024). Harnessing the full potential of a global forest-based bioeconomy through non-timber products: Beyond logs, biotechnology, and high-income countries. Forest Policy and Economics, 158, 103105.

Chapagain, D. J., Meilby, H., Baniya, C. B., Budha-Magar, S., & Ghimire, S.K. (2021). Illegal harvesting and livestock grazing threaten the endangered orchid Dactylorhiza hatagirea (D. Don) Soó in Nepalese Himalaya. Ecology and Evolution, 11(11), 6672-6687.

Chauhan, H. K., Oli, S., Bisht, A. K., Meredith, C., & Leaman, D. (2021). Review of the biology, uses and conservation of the critically endangered endemic Himalayan species Nardostachys jatamansi (Caprifoliaceae). Biodiversity and Conservation, 30, 3315–3333.

Childs, G., Craig, S., Beall, C. M., & Basnyat, B. (2014). Depopulating the Himalayan highlands: Education and outmigration from ethnically Tibetan communities of Nepal. Mountain Research and Development, 34(2), 85–94.

Choudhary, D., Kala, S., Todaria, N., Dasgupta, S., & Kollmair, M. (2013). Marketing of Bay Leaf in Nepal and Northern India: Lessons for Improving Terms of Participation of Small Farmers in Markets. Small-Scale Forestry 12(2), 289–305.

CITES. (2018). Non-timber forest products: CITES implementation for medicinal plant species. Document PC24 Inf. 7. Geneva, Switzerland.

Craig, S. R. & Glover, D. M. (2009). Conservation, Cultivation, and Commodification of Medicinal Plants in the Greater Himalayan-Tibetan Plateau. Asian Medicine, 5, 219-242.

Cunningham, A. B., Brinckmann, J. A., Schippmann, U., & Pyakurel, D. (2018). Production from both wild harvest and cultivation: The cross-border Swertia chirayita (Gentianaceae) trade. Journal of Ethnopharmacology, 225, 42-52.

Das, A. K., Oli, B. N., Smith-Hall, C., Rayamajhi, S., Ghimire, S., & Dhakal, S.P. (Eds.) (2018). Wild Harvests, Governance, and Livelihoods in Asia: Proceedings from the International Conference, TGG-N Project, Kathmandu.

DFSC. (Undated). National Quota Fixation for Jatamansi (Nardostachys jatamansi DC) Ensuring Sustainable Management and Conservation in Nepal. Department of Forests and Soil Conservation, Kathmandu.

Dovydaitis, E. (2017). Political Ecology of Medicinal Plant Use in Rural Nepal: Globalization, Environmental Degradation, and Cultural Transformation. Thesis, University of Central Florida, Orlando.

EU. (2016). Regulation (EU) 2016/2031 of the European Parliament of the Council of 26 October 2016 on protective measures against pests of plants. European Parliament, Brussels.

EU. (2018). Short summary of conclusions. The 85^th^ Meeting of the Scientific Review Group on Trade in Wild Fauna and Flora, Brussels 3^rd^ December 2018.

Fold, N., Pyakurel, D., Pouliot, M., & Smith-Hall, C. (2023). Global production networks and medicinal plants: upstream actors in Nepal. Geographical Journal, 189, 455–468.

GIZ. (2017). MAPs and essential oils from Nepal: market analysis and market entry strategies in five selected markets. Deutsche Gesellschaft für Internationale Zusammenarbeit, Bonn, Germany. 159 pp.

GoN. (2007). Plant Protection Act 2064. Government of Nepal, Kathmandu, Nepal.

GoN. (2010). Plant Protection Rules 2066. Government of Nepal, Kathmandu, Nepal.

GoN. (2011). Industrial Policy 2067. Government of Nepal, Kathmandu, Nepal.

GoN. (2011). Nepal Gazette (60-38-5) 2067. Government of Nepal, Kathmandu, Nepal.

GoN. (2012). NTFPs Resource Inventory Guideline 2069. Government of Nepal, Ministry of Forests and Soil Conservation, Department of Forests, Kathmandu, Nepal.

GoN. (2014). Nepal National Biodiversity Strategy and Action Plan 2014-2020. Government of Nepal, Ministry of Forests and Soil Conservation, Kathmandu, Nepal.

GoN. (2015). Nepal Gazette (65-26-3) 2072. Government of Nepal, Kathmandu, Nepal.

GoN. (2015). Nepal REDD+ Strategy Part 1: Operational Strategy. Government of Nepal, Ministry of Forests and Soil Conservation, REDD Implementation Centre, Kathmandu, Nepal.

GoN. (2016). Forestry Sector strategy 2016-2025. Government of Nepal, Ministry of Forests and Soil Conservation, Kathmandu, Nepal.

GoN. (2017). An act to regulate and control international trade in endangered wild fauna and flora. Government of Nepal, Ministry of Forests and Soil Conservation, Kathmandu, Nepal

GoN. (2017). Forest Investment Program. Government of Nepal, Ministry of Forests and Soil Conservation, Kathmandu, Nepal.

GoN. (2018). Nepal Gazette (68-34-3) 2075. Government of Nepal, Kathmandu, Nepal.

GoN. (2019). Forest Act 2076. Government of Nepal, Ministry of Forests and Environment, Kathmandu, Nepal.

GoN. (2019). Grant allocation procedure for development of medicinal plants - 2075 (2019). Government of Nepal, Ministry of Forests and Environment, Department of Plant Resources, Kathmandu, Nepal.

GoN. (2019). Grant allocation procedure for promotion and development of forest based enterprises 2075. Government of Nepal, Ministry of Forests and Environment, Department of Forests and Soil Conservation, Kathmandu, Nepal.

GoN. (2019). National Forest Policy 2075. Government of Nepal, Ministry of Forests and Environment, Kathmandu, Nepal.

GoN. (2020). The Fifteenth Plan FY 2019/20 – 2023/24. Government of Nepal, National Planning Commission, Kathmandu, Nepal.

GoN. (2022). Forest Regulation 2079. Government of Nepal, Ministry of Forests and Environment, Kathmandu, Nepal.

GoN. (2023). National Adaptation Plan 2080. Government of Nepal, Ministry of Forests and Environment, Kathmandu, Nepal.

Goraya, G. S., & Ved, D. K. (2017). Medicinal Plants in India: An Assessment of their Demand and Supply. National Medicinal Plants Board, Ministry of AYUSH, Government of India, New Delhi.

Gurung, K. (2013). Study on Quality Issues of Medicinal and Aromatic Plants (MAPs) Sector in Nepal. The Physikalisch-Technische Bundesanstalt, Braunschweig.

Hajjar, R., Oldekop, J. A., Cronkleton, P., Newton, P., Russell, A. J. M., & Zhou, W. (2021). A global analysis of the social and environmental outcomes of community forests. Nature Sustainability, 4, 216-224.

Harilal, M. S. (2009). Commercialising traditional medicine: Ayurvedic manufacturing in Kerala. Economic and Political Weekly, 16, 44–51.

He, J. (2023). Commercial fungi, indigenous communities, and the bioeconomy transition in Southwest China. In: Smith-Hall, C., Chamberlain, J.L. (Eds.), The bioeconomy and non-timber forest products. Routledge, New York, pp. 43-56.

He, J., Smith-Hall, C., Zhou, W, Zhou, W., Wang, Y., & Fan, B. (2022). Uncovering caterpillar fungus (Ophiocordyceps sinensis) consumption patterns and linking them to conservation interventions. Conservation Science and Practice, 4(8), e12759.

Hinsley, A., & Sas-Rolfes, M. ‘t. (2020). Wild assumptions? Questioning simplistic narratives about consumer preferences for wildlife products. People and Nature, 2(4), 972-979.

Hinsley, A., Milner-Gulland, E. J., Cooney, R., Timoshyna, A., Ruan, X., & Lee, T. M. (2019). Building sustainability into the Belt and Road Initiative’s Traditional Chinese Medicine trade. Nature Sustainability, 3, 96-100.

HMGN (2004) Nepal National Action Programme on Land Degradation and Desertification. His Majesty’s Government of Nepal, Ministry of Population and Environment, Kathmandu, Nepal

HMGN. (1972). Plant Protection Act. His Majesty’s Government of Nepal, Kathmandu, Nepal.

HMGN. (1973). National Parks and Wildlife Conservation Act. His Majesty’s Government of Nepal, Ministry of Forests and Environment, Kathmandu, Nepal.

HMGN. (1976). National Forest Policy. His Majesty’s Government of Nepal, Ministry of Forests and Environment, Kathmandu, Nepal.

HMGN. (1988). Master Plan for the Forestry Sector Nepal. His Majesty’s Government of Nepal, Ministry of Forests and Environment, Kathmandu, Nepal.

HMGN. (1988). National Conservation Strategy. His Majesty’s Government of Nepal, Kathmandu, Nepal.

HMGN. (1992). Trade and Transit Agreement. His Majesty’s Government of Nepal, Kathmandu, Nepal.

HMGN. (1993). Forest Rules. His Majesty’s Government of Nepal, Ministry of Forests and Environment, Kathmandu, Nepal.

HMGN. (1993). National Environmental Impact Assessment Guidelines. His Majesty’s Government of Nepal, Kathmandu, Nepal.

HMGN. (1993). Nepal Environmental Policy and Action Plan. His Majesty’s Government of Nepal, Environment Protection Council, Kathmandu, Nepal.

HMGN. (1995). Forest Regulation. His Majesty’s Government of Nepal, Ministry of Forests and Environment, Kathmandu, Nepal.

HMGN. (1996). Conservation Area Management Rules 2053. His Majesty’s Government of Nepal, Ministry of Forests and Soil Conservation, Kathmandu, Nepal

HMGN. (1996a). The Environment Protection Act. His Majesty’s Government of Nepal. Kathmandu, Nepal

HMGN. (2000). Forestry Sector Policy. His Majesty’s Government of Nepal, Ministry of Forests and Soil Conservation, Kathmandu, Nepal.

HMGN. (2000). Local Self-Governance Regulation. His Majesty’s Government of Nepal, Kathmandu, Nepal

HMGN. (2001). Nepal Gazette (51-36-3) 2058. His Majesty’s Government of Nepal, Kathmandu, Nepal.

HMGN. (2002). Nepal Biodiversity Strategy. His Majesty’s Government of Nepal, Ministry of Forests and Soil Conservation, Kathmandu, Nepal.

HMGN. (2004). Herbs and NTFPs Development Policy 2061. His Majesty’s Government of Nepal, Ministry of Forests and Soil Conservation, Kathmandu, Nepal.

HMGN. (2005). Guidelines for promotion and development of NTFP-based enterprises. His Majesty’s Government of Nepal, Ministry of Forests and Soil Conservation, Kathmandu, Nepal.

HMGN. (2006). Nepal Biodiversity strategy implementation plan. His Majesty’s Government of Nepal, Ministry of Forests and Soil Conservation, Kathmandu, Nepal.

IUCN. (2023). Integrated tiger habitat conservation programme. IUCH, Gland.

Kafle, G., Sharma, I. B., Siwakoti, M., & Shrestha, A. K. (2018). Demand, end-uses, and conservation of alpine medicinal plant Neopicrorhiza scrophulariiflora (Pennell) D.Y. Hong in Central Himalaya. Evidence-Based Complementary and Alternative Medicine, 6024263.

Kala, C. P. (2015). Medicinal and aromatic plants: Boon for enterprise development. Journal of Applied Research in Medicinal and Aromatic Plants, 2, 134–139.

Karki, M. B., & Chowdhary, C. L. (2019). Non-timber forest products (NTFP) and agro-forestry subsectors: potential for growth and contribution in agriculture development. In Thapa, G., Kumar, A., & Joshi, P. K. (Eds.) Agricultural Transformation in Nepal, pp. 385-419. Springer: Singapore.

Kloos, S., Madhavan, H., Tidwell, T., Blaikie, C., & Cuomu, M. (2020). The transnational Sowa Rigpa industry in Asia: New perspectives on an emerging economy. Social Science and Medicine, 245, 112617.

Lamichhane, D., & Karna, N. (2010). Harvesting methods of Cinnamomum tamala leaves in private land: a case study from Udayapur district, Nepal. Banko Janakari, 19(2), 20–24.

Larsen, H. O. (2005). Impact of replanting on regeneration of the medicinal plant Nardostachys grandiflora DC. (Valerianaceae). Economic Botany, 59(3), 213-220.

Larsen, H.O., & Smith, P.D. (2004). Stakeholder perspectives on commercial medicinal plant collection in Nepal. Mountain Research and Development, 24, 141–148.

Li, X., Liu, Q., Li, W., Li, Q., Qian, Z., Liu, X., & Dong, C. (2019). A breakthrough in the artificial cultivation of Chinese cordyceps on a large-scale and its impact on science, the economy, and industry. Critical Reviews in Biotechnology, 39(2), 181–191.

Liu, H., Gale, S. W., Cheuk, M. L., & Fischer, G. A. (2019). Conservation impacts of commercial cultivation of endangered and overharvested plants. Conservation Biology, 33(2), 288–299.

Liu, H., Luo, Y. B., Heinen, J., Bhat, M., & Liu, Z. J. (2014). Eat your orchid and have it too: A potentially new conservation formula for Chinese epiphytic medicinal orchids. Biodiversity and Conservation, 23(5), 1215–1228.

Luintel, H., Bluffstone, R. A., & Scheller, R. M. (2018). The effects of the Nepal community forestry program on biodiversity conservation and carbon storage. PLoS ONE, 13(6), e0199526.

Madsen, S. T., & Smith-Hall, C. (2023). Wild harvesting or cultivation of commercial environmental products: A theoretical model and its application to medicinal plants. Ecological Economics, 205, 107701.

Meilby, H., Smith-Hall, C., Byg, A., Larsen, H. O., Nielsen, Ø. J., Puri, L., & Rayamajhi, S. (2014). Are forest incomes sustainable? Firewood and timber extraction and forest productivity in community managed forests in Nepal. World Development, 64, S113-S124.

MoFE. (2021). Current status of community based forest management (CBFM) in Nepal 2020. Kathmandu, Ministry of Forests and Environment.

Mofokeng, M. M., Du Plooy, C. P., Araya, H. T., Amoo, S.O., Mokgehle, S.N., Pofu, K.M., & Mashela, P. W. (2022). Medicinal plant cultivation for sustainable use and commercialisation of high-value crops. South African Journal of Science, 118(7/8), 12190.

Ojha, R. B., Atreya, K., Kristiansen, P., Devkota, D., & Wilson, B. (2022). A systematic review and gap analysis of drivers, impacts, and restoration options for abandoned croplands in Nepal. Land Use Policy, 120, 106237.

Olsen, C. S. (1998). The trade in medicinal and aromatic plants from Central Nepal to Northern India. Economic Botany, 52, 279–292.

Olsen, C. S. (2005). Valuation of commercial Central Himalayan medicinal plants. Ambio, 34: 607–610.

Olsen, C. S., & Bhattarai, N. K. (2005). A typology of economic agents in Himalayan plant trade. Mountain Research and Development, 25(1), 37-43.

Olsen, C. S., & Helles, F. (2009). Market efficiency and benefit distribution in medicinal plant markets: empirical evidence from South Asia. International Journal of Biodiversity Science and Management, 5(2), 53-62.

Ostrom, E. (2009). A general framework for analyzing sustainability of social-ecological systems. Science, 325(5939), 419–422.

Pant, B., Rai, R. K., Wallrapp, C., Ghate, R., Shrestha, U. B., & Ram, A. (2017). Horizontal integration of multiple institutions: solution for yarsagumba related conflicts in the Himalayan Region of Nepal? International Journal of the Commons, 11(1), 464-486.

Paudel, G., Carr, J., & Munro, P. G. 2022. Community forestry in Nepal: a critical review. International Forestry Review, 24(1), 43-58.

Poudeyal, M. R., Meilby, H., Shrestha, B. B., & Ghimire, S. K. (2019). Harvest effects on density and biomass of Neopicrorhiza scrophulariiflora vary along environmental gradients in the Nepalese Himalayas. Ecology and Evolution, 9(13), 7726-7740.

Pyakurel, D. (2020). Trade and conservation of medicinal plants, fungi, and lichens in Nepal: An application of global production network theory. PhD Dissertation, Agriculture and Forestry University, Rampur, Nepal.

Pyakurel, D., Bhattarai Sharma, I., & Smith-Hall, C. (2018). Patterns of change: The dynamics of medicinal plant trade in far-western Nepal. Journal of Ethnopharmacology, 224, 323–334.

Pyakurel, D., & Smith-Hall, C. (2023). Creating, enhancing, and capturing environmental product values – the case of commercial medicinal, spice, and food plants from lowland Nepal. Manuscript.

Pyakurel, D., Smith-Hall, C., Bhattarai-Sharma, I., & Ghimire, S. K. (2019). Trade and conservation of Nepalese medicinal plants, fungi, and lichens. Economic Botany, 73(4), 505-521.

Sas-Rolfes, M. ‘t, Challender, D. W. S., Hinsley, A., Veríssimo, D., & Milner-Gulland, M. J. (2019). Illegal wildlife trade: scale, processes, and governance. Annual Review of Environment and Resources, 44, 201-228.

SAWEN. (2022). Strategic Plan 2022-2026. South Asia Wildlife Enforcement Network, Kathmandu. 14pp.

Smith-Hall, C., Chapagain, A., Das, A. K., Ghimire, S. K., Pyakurel, D., Treue, T., & Pouliot, M. (2020). Trade and conservation of medicinal and aromatic plants – an annotated bibliography for Nepal. Sopan Press, Kathmandu.

Smith-Hall, C., Pouliot, M., Pyakurel, D., Fold, N., Chapagain, A., Ghimire, S., Meilby, H., Kmoch, L., Chapagain, D. J., Das, A., Jun, H., Nepal, K., Poudeyal, M. R., Kafle, G., & Larsen, H. O. (2018). Data collection instruments and procedures for investigating national-level trade in medicinal and aromatic plants. IFRO Documentation 2018/2. University of Copenhagen, Department of Food and Resource Economics, Copenhagen.

Smith-Hall, C., Pyakurel, D., Meilby, H., Pouliot, M., Ghimire, P., Ghimire, S., Madsen, S.T., Paneru, Y.R., Subedi, B., Timoshyna, A., & Treue, T. (2023). The sustainability of trade in wild plants – a data-integration approach tested on critically endangered Nardostachys jatamansi. PNAS Nexus, 2, 1-9.

Subedi, B. S. (2008). Transfer of training: improving the effectiveness of employee training in Nepal. Journal of Education and Research, 1(1), 51-61.

TRAFFIC. (2023a). Guidelines for Risk Assessment in the Utilisation of Medicinal Fauna and Flora. TRAFFIC, Cambridge. 43pp.

TRAFFIC. (2023b). Legal and sustainable wild species trade. TRAFFIC, Cambridge, 65pp.

WHO. (2003). WHO guidelines on good agricultural and collection practices (GACP) for medicinal plants. World Health Organization, Geneva. 72pp.

World Bank. (2017). Climbing higher: toward a middle-income Nepal. Washington DC: The World Bank. 54pp.

World Bank. (2018). Strategic segmentation analysis: Nepal. Medicinal and aromatic plants. Washington DC: The World Bank. 61pp.

World Bank. (2020). Nepal Development Update - July 2020. Washington DC: The World Bank.

World Bank. (2023a). Remittances remain resilient but are slowing. Migration and Development Brief 33. Washington DC: The World Bank.

World Bank (2023b). Nepal Development Update – October 2023. Washington DC: The World Bank. 48pp.

Yadav, B. K. V. (2013). Marketing trend and channels of Asparagus racemosus in Nepal: A study from Sarlahi district. World Journal of Agricultural Sciences, 1(4), 148-157.
